# Reconstructed Lost Native American Populations from Eastern Brazil Are Shaped by Differential Jê/Tupi Ancestry

**DOI:** 10.1093/gbe/evz161

**Published:** 2019-07-22

**Authors:** Alex Mas-Sandoval, Lara R Arauna, Mateus H Gouveia, Mauricio L Barreto, Bernardo L Horta, Maria Fernanda Lima-Costa, Alexandre C Pereira, Francisco M Salzano, Tábita Hünemeier, Eduardo Tarazona-Santos, Maria Cátira Bortolini, David Comas

**Affiliations:** 1 Departamento de Genética, Instituto de Biociências, Universidade Federal do Rio Grande do Sul, Porto Alegre, Rio Grande do Sul, Brazil; 2 Departament de Ciències Experimentals i de la Salut, Institute of Evolutionary Biology (CSIC-UPF), Universitat Pompeu Fabra, Barcelona, Spain; 3 Departamento de Biologia Geral, Instituto de Ciências Biológicas, Universidade Federal de Minas Gerais, Belo Horizonte, Brazil; 4 Center for Research on Genomics and Global Health, National Institutes of Health, Bethesda, Maryland; 5 Instituto de Saúde Coletiva, Universidade Federal da Bahia, Salvador, Bahia, Brazil; 6 Center for Data and Knowledge Integration for Health, Institute Gonçalo Muniz, Fundação Oswaldo Cruz, Salvador, Bahia, Brazil; 7 Programa de Pós-Graduação em Epidemiologia, Universidade Federal de Pelotas, Rio Grande do Sul, Brazil; 8 Instituto de Pesquisa Rene Rachou, Fundação Oswaldo Cruz, Belo Horizonte, Minas Gerais, Brazil; 9 Instituto do Coração, Universidade de São Paulo, São Paulo, Brazil; 10 Department of Genetics and Evolutionary Biology, Biosciences Institute, University of São Paulo, São Paulo, Brazil

**Keywords:** human genome diversity, Native American groups, Brazil

## Abstract

After the colonization of the Americas by Europeans and the consequent Trans-Atlantic Slave Trade, most Native American populations in eastern Brazil disappeared or went through an admixture process that configured a population composed of three main genetic components: the European, the sub-Saharan African, and the Native American. The study of the Native American genetic history is challenged by the lack of availability of genome-wide samples from Native American populations, the technical difficulties to develop ancient DNA studies, and the low proportions of the Native American component in the admixed Brazilian populations (on average 7%). We analyzed genome-wide data of 5,825 individuals from three locations of eastern Brazil: Salvador (North-East), Bambui (South-East), and Pelotas (South) and we reconstructed populations that emulate the Native American groups that were living in the 16th century around the sampling locations. This genetic reconstruction was performed after local ancestry analysis of the admixed Brazilian populations, through the rearrangement of the Native American haplotypes into reconstructed individuals with full Native American ancestry (51 reconstructed individuals in Salvador, 45 in Bambui, and 197 in Pelotas). We compared the reconstructed populations with nonadmixed Native American populations from other regions of Brazil through haplotype-based methods. Our results reveal a population structure shaped by the dichotomy of Tupi-/Jê-speaking ancestry related groups. We also show evidence of a decrease of the diversity of nonadmixed Native American groups after the European contact, in contrast with the reconstructed populations, suggesting a reservoir of the Native American genetic diversity within the admixed Brazilian population.

## Introduction

Genetic evidence shows that the Native peoples of South America trace their origins to an ancestral population that populated North America from Beringia around 15,000 years ago and reached South America in a few hundred years (Bonatto and Salzano 1997; Hey et al. 2005; Tamm et al. 2007; Ray et al. 2010; Reich et al. 2012; Raghavan et al. 2014, 2015; Skoglund et al. 2015; Llamas et al. 2016; Moreno-Mayar, Potter, et al. 2018). A second migration wave of the same ancestral population followed, originating in Mesoamerica and moving once again toward South America (Moreno-Mayar, Vinner, et al. 2018; Posth et al. 2018). It has been estimated that around 25 million Native Americans were living in South America in the late 15th at the time of the beginning of the colonization of the continent by the Europeans, with estimates varying from 4.2 to 48.8 million people (Kroeber 1939; Dobyns 1966; Smith 1979; Thornton 1990; Adhikari et al. 2017). European historical records describe a population scenario of Brazil during the 16th century in which Tupi populations from the Tupi-Guarani linguistic family were living along the coast, whereas non-Tupi populations named generically *Tapuia* (mostly Macro-Jê-speaking populations) inhabited the hinterlands. However, this coastal Tupi *continuum* was broken by *Tapuia* in the mouth of the Paraíba River, in the Southern Bahia, and in the Maranhão areas (Soares de Souza 1879; Métraux 1927; Carneiro da Cunha 1998). It is claimed that these non-Tupi populations occupied a wider coastal extension before Tupi populations expelled them out of the coastal regions (Cardim 1925; Métraux 1927; Carneiro da Cunha 1998).

Various European migration waves populated South America since the conquest, from first Portuguese and Castilian settlers to a last migration pulse to the Southern Cone in the 19th and 20th centuries (Adhikari et al. 2017). From the 16th to the 19th centuries, European colonizers brought, from sub-Saharan Africa to the Americas, more than 9 million enslaved people (∼4 million in Brazil) to work in labor-intensive plantations (Voyages Database 2016; Adhikari et al. 2017).

Consequently, most of the ∼200 million people who live today in Brazil belong to urban admixed Brazilian populations that are the result of a series of admixture events of three main continental genetic components: Native American, European, and sub-Saharan African. These three ancestral components are admixed in diverse proportions in the different areas of the country. The Native American component is usually found in the lowest proportion and was mostly admixed with the other components soon after their arrival to the continent (Kehdy et al. 2015). Only 0.8 million people in Brazil are self-declared Native Americans, most of them living in indigenous lands in the North and West of Brazil, in the basins of the rivers Amazonas, Paraguay, and Paraná (supplementary fig. S1, Supplementary Material online) (IBGE 2010). Most speak one language from the four largest linguistic stocks, in Brazil: Macro-Jê, which includes Jê family; Carib; Arawak; and Tupi, which includes Tupi-Guarani family (that in turn comprises Guarani, Tupi, and Northern branches, among others) (Campbell and Grondona 2012).

Genetic studies on populations from the American continent have focused on the description of admixture processes occurred during the last 500 years (Bryc et al. 2010; Johnson et al. 2011; Wall et al. 2011; Kidd et al. 2012; Moreno-Estrada et al. 2013; Martin et al. 2017; Fortes-Lima et al. 2018). A number of these studies have also been focused on the analyses of the substructure of each of the main genetic components of admixed American populations, which has allowed to disentangle previous demographic and admixture processes. Most of the methods used for these studies had the advantage of analyzing populations with a substantial proportion of the admixed component (∼30%), which can be identified through local ancestry analysis, and subsequently masked, allowing to focus only on the target components (Brisbin et al. 2012; Gravel et al. 2013; Maples et al. 2013; Moreno-Estrada et al. 2013; Homburger et al. 2015; Kehdy et al. 2015; Montinaro et al. 2015; Chacón-duque et al. 2018). In parallel, during the last years, haplotype-based methods have improved the power to characterize the structure of human populations with complex demographic histories (Hellenthal et al. 2014; Montinaro et al. 2015; van Dorp et al. 2015; Patin et al. 2017).

Here, we use genome-wide data of present urban admixed Brazilians and a combination of local ancestry and haplotype-based methods to reconstruct virtual individuals with full Native American ancestry and analyze their genetic origins. Urban Brazilians usually exhibit low amounts of the Native American component (7% on average) (Kehdy et al. 2015), making the study of their original gene pool challenging. To overcome this limitation and shed light on the Native American history before the arrival of the Europeans, we reconstructed individuals that emulate the ancestral Native American populations that admixed with European and/or sub-Saharan African groups, resulting in the current Brazilian population. With this purpose, the Native American ancestral fragments from the admixed populations were extracted through a local ancestry analysis and reorganized to build the reconstructed individuals. This process was done without breaking or overlapping the fragments to keep the haplotypic structure within the Native American fragments and allow a haplotype-based methods approach.

In conclusion, we present a new approach that allowed a high-resolution study of the population substructure and the genetic history of the Native American ancestral populations of three current Brazilian urban admixed populations. We analyzed, traced, and compared the Native American component of the Brazilian Atlantic Coast (North-East and South) and the Brazilian Plateau, an area where most of Native American populations vanished or admixed to become urban admixed populations. A similar approach could be applied to other admixed populations, even those with genetic components in extreme low frequency, therefore expanding the boundaries of the study of extinct populations beyond the limitations given by the availability of ancient DNA. Moreover, having started from current individuals, we focus on the study of populations that, by definition, were the ancestral of current Brazilians.

## Materials and Methods

### Data Samples and Quality Control

**Fig. 1.**
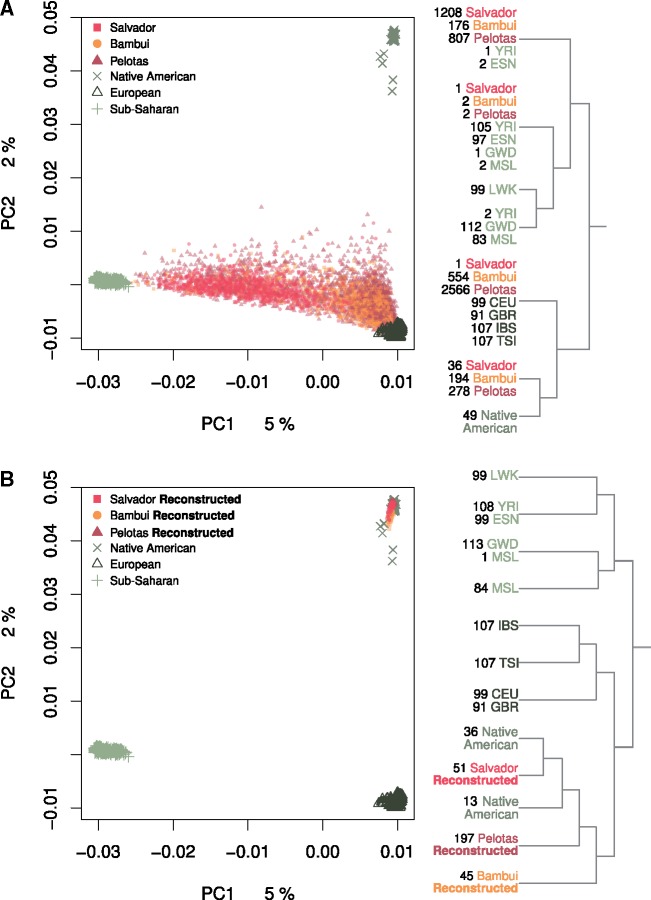
—PCA with sub-Saharan African, European, and Native American individuals with the admixed Brazilians (*A*); and the reconstructed Native American individuals (*B*) from Bambui, Pelotas, and Salvador. On the right of each PCA, FineStructure dendrograms of the same data set are shown. Labels from branches represent the consensus clusters with the number of individuals per population through three seeds simplified after joining sister branches (supplementary figs. S9–S11 and S19–S21 and supplementary tables S3 and S4, Supplementary Material online).

Data set A includes genome-wide data from admixed population-based cohorts from the Brazilian EPIGEN initiative. The samples are from Salvador (*n* = 1,246), Bambui (*n* = 926), and Pelotas (*n* = 3,653) cities genotyped by the Illumina HumanOmni2.5–8v1 array (Kehdy et al. 2015), merged with phase III 1000 Genomes Project individuals: sub-Saharan Africans (YRI, LWK, MSL, ESN, and GWD), Europeans (CEU, GBR, TSI, and IBS), and admixed Americans (CLM, MXL, PEL, and PUR). This data set also includes the Brazilian nonadmixed Native American samples from Skoglund et al. (2015), which includes Apalai (*n* = 4) and Arara (*n* = 4) from Carib linguistic family and Xavante (*n* = 11) from Jê linguistic family. From the Tupi linguistic stock, the data set includes six populations: Guarani Kaiowá (*n* = 10) and Guarani Ñandevá (*n* = 7) from Guarani branch within Tupi-Guarani linguistic family; Urubu-Kaapor (*n* = 3) from Northern branch within Tupi-Guarani linguistic family; Karitiana (*n* = 5) from Mondé linguistic family; and Surui (*n* = 4) and Zoro (*n* = 1), from the Arikem family (Campbell and Grondona 2012). Most of these nonadmixed Native American groups have mainly hunter-gatherer/forager lifestyles, because their habits of life and diet remain, or remained until recently, similar to those before the contact with non-Native Americans. Individuals with evidence of European or sub-Saharan African admixture in a Principal Component analysis (PCA) and with Native American ancestry below 0.99 in an ADMIXTURE analysis were removed and not included in the Data set B (fig. 1 and supplementary figs. S2 and S3, Supplementary Material online). Data set B comprises the same populations of Data set A, but the Brazilian admixed individuals from Bambui, Pelotas, and Salvador are replaced by the reconstructed Native American individuals of those three locations (supplementary tables S1–S3, Supplementary Material online).

Single-nucleotide polymorphisms (SNPs) missing in more than 10% of the individuals and individuals with more than 10% of missing SNPs were excluded. Those SNPs that failed Hardy–Weinberg test at 0.05 significance threshold were also excluded. The kinship coefficients for each possible pair of individuals within a population were computed using the method implemented in the Relatedness Estimation in Admixed Populations software as described in Kehdy et al. (2015). Following this, a maximized data set without pairs of individuals closer than second-degree relatives (Relatedness Estimation in Admixed Populations kinship coefficient >0.10) was finally selected. SNP pruning to keep only markers in linkage equilibrium was applied before PCA, ADMIXTURE, f3, and f4 analysis, using a pairwise linkage disequilibrium maximum threshold of 0.5, a window size of 50, and a shift step of 5, after which 131,271 SNPs were left. PLINK 1.07 was used in all filters except the kinship analysis.

Data were phased with SHAPEIT (Delaneau et al. 2011; O’Connell et al. 2014) with a population-averaged genetic map from the HapMap phase II and the 1000 Genomes data set phase III as a reference panel. The SNPs that do not align with the reference panel were removed.

### Estimation of the Local Ancestry Proportions

Local ancestry was performed with a data set composed of 1,845,872 shared SNPs between the Illumina HumanOmni2.5–8v1 array and phase III 1000 Genomes Project individuals. The data set comprised the analyzed populations from Kehdy et al. (2015), which included the admixed individuals from Salvador, Bambui, and Pelotas together with reference Native American populations Shimaa (23 individuals) and Ashaninka (52 individuals). Other Native American samples (Quechuas, Ashaninkas, Shimaas, and Aymara; Matsiguengas, Queros, Uros, and Moches) (Harris et al. 2018) were included in the data set in order to have a wider haplotype spectrum for Native Americans. Reference populations from sub-Saharan Africa included 31 samples from Botswana (Crawford et al. 2017), 83 samples from Ghana (from National Cancer Institute Survey of Prostate Cancer in Accra, Gouveia et al. 2019), and 73 samples from Gambia (from 1000 Genomes phase III [Gibbs et al. 2015]). These three reference ancestral populations were used to split the chromosomes by local ancestry in fragments of each of the three ancestries: Native American, European, and sub-Saharan African. Phased chromosomes with SHAPEIT (Delaneau et al. 2011; O’Connell et al. 2014), as detailed in Kehdy et al. (2015), were used as input for RFMix v.1.5.4 Pophased (Maples et al. 2013)*.* The number of generations since the admixture event (parameter -*G*) was fixed at 20 (∼500 years) and the number of trees to generate per random forest (parameter -*t*) in 500. Window lengths (parameter -*w*) were set to 0.2 cM.

### Genetic Reconstruction of Lost Native American Populations

For each of the three admixed Brazilian samples (Salvador, Bambui, and Pelotas), local ancestry Native American fragments were rearranged without breaking nor overlapping fragments, using each fragment only once, to build chromosomes with full Native American ancestry that configured the reconstructed Native American individuals (see supplementary figs. S4–S8, Supplementary Material online).

For all the analyzed individuals by local ancestry, only the windows assigned to Native American ancestry with a posterior probability higher than 0.8 were kept, discarding all windows with European or sub-Saharan African ancestry. Consecutive Native American ancestry windows were concatenated in a single fragment. The fragments were sorted, for each autosome, by their start base pair position, from lower to higher, and sorted randomly in case two or more fragments started at the same base pair position. Then, each *rearranged chromosome* of the future *reconstructed individual* was reconstructed by picking up fragments from the fragments list sorted by the first position, from the beginning to the end of the chromosome. The only condition was that the subsequent fragment could not overlap the previous, which means that the start position of the next fragment had to be higher than the end position of the previous one (supplementary fig. S4, Supplementary Material online). Unless the subsequent fragment started at the immediately following base pair after the precedent fragment, a gap was left between the fragments, which was considered as a missing fragment. Rearranged full Native American ancestry chromosomes tended to have longer gaps between fragments, and therefore more missing fragments, as the rearrangement process progressed since the Native American fragments pool decreased. Therefore, in order to select the rearranged chromosomes with higher percentage of nonmissing fragments for each autosome, we set a minimum threshold of 95% of base pairs of the chromosome covered by the Native American fragments (supplementary figs. S4–S7, Supplementary Material online).

One hundred iterations of this reconstruction jigsaw puzzle process were performed for each autosome. For each iteration, we randomly sorted each set of fragments starting in the same position, which made each iteration unique. Therefore, after 100 iterations, 100 rearrangement processes were made per each autosome and population. Because of the established minimum threshold fixed previously of 95% of base pairs of a rearranged chromosome being covered by the fragments, there was a given number of rearranged chromosomes above the threshold in each iteration, per each population. Thus, we selected the best iteration as the one which could get more rearranged chromosomes above the threshold, for each population and each autosome from 1 to 22 (supplementary fig. S5 and supplementary table S4, Supplementary Material online). In order to obtain reconstructed diploid individuals with 22 pairs of chromosomes, the autosome with the lowest number of rearranged chromosomes in each population set the number of reconstructed chromosomes in this population. The number of reconstructed individuals was the half of this value, as two random rearranged chromosomes were paired to build a reconstructed diploid individual. Finally, reconstructed populations were rephased together with reference Native Americans to build Data set B, to analyze the structure of Native American populations.

### PCA, ADMIXTURE, f3, and f4

*PCA* *s* were computed with the SmartPCA program from the EIGENSTRAT stratification correction software found in EIGENSOFT 4.2 package (Patterson et al. 2006). ADMIXTURE (Alexander et al. 2009) was run for *k* = 2 to *k* = 9 and three iterations in each data set. f3 and f4 were computed with qpDstat and qp3Pop commands from Admixtools 3.0 (Patterson et al. 2012).

### Haplotype-Based Methods

*FineStructure 2.1.0* and *ChromoPainter* (Lawson et al. 2012) were used to analyze the genetic structure of Salvador, Bambui, and Pelotas individuals. ChromoPainter (*fs cp*) was run in each chromosome with the following flags: *-in*, *-iM*, and *-i 15*. Thus, the output for each receptor individual was iterated 15 times to find the best *n* and *M* values (related to effective population size and mutation rate, respectively) and the mean value for all the individuals per each autosome was computed. Once the parameters were established, ChromoPainter was run again to compute the squared coancestry matrix. Finally, all autosomal chromosomes were summed to obtain the genome-wide squared coancestry matrix, in which all samples are individually considered as recipients and donors (not allowing selfcopying). Before running *FineStructure*, *fs combine* was run in order to compute the parameter *c*, needed for *FineStructure*. *FineStructure* (*fs fs*) was run in two steps: mcmc and tree computation with three random seeds set.

This process was run with two sets of populations: Data set A and Data set B in order to compare reconstructed Native American individuals with their original admixed individuals from each population (Bambui, Pelotas, and Salvador). The parameters *n* and *M* for Data set A were the same as the ones computed for the Data set B because the computational limitations in running ChromoPainter with a large data set. We set clusters from the fineSTRUCTURE results of Data set B for subsequent analyses. We set the consensus clusters at the dendrogram height of 3, as the lowest height that allowed us to analyze the Guarani individuals as a single cluster and not as two mixed clusters of Guarani Ñandeva and Guarani Kaiowá populations.

ChromoPainter v2 was used to obtain a nonsquared coancestry matrix, where a restricted set of populations can play as haplotype donor populations. We run ChromoPainter in this way with the Data set B, where the reconstructed individuals were the recipient individuals and the nonadmixed Native Americans are the possible donors. The *n* and *M* parameters were computed again for each chromosome from the average values of the individuals used in this run. The distributions of the total chunklength received by the recipient populations for a given donor were compared between them. The significance mean difference was tested by Wilcoxon test. Differences accounting for a Bonferroni multiple test corrected *P* value lower than 0.005 were considered significant.

### Effective Population Size and Genetic Diversity

Effective population size (Ne) was computed with IBDNe through intrapopulation IBD values of 4-cM windows, which were computed by IBDseq (Browning and Browning 2015). The values for the log(Ne) curves were filtered by a threshold of a 95% CI narrower than 2.5. We have also computed IBD through Refined IBD (Browning and Browning 2013) and merge IBD as described in Browning et al. (2018) and then IBDseq (Browning and Browning 2015) to compute the effective population size curves. Genetic diversity has been computed per SNP position within each population through vcftools(1) with –site-pi flag.

## Results

### Genetic Reconstruction of Lost Native American Samples and Population Structure

The genetic structure of the admixed Brazilian populations is driven by the admixture proportions of Native American, European, and sub-Saharan African components. A PCA shows most individuals from Bambui, Pelotas, and Salvador spread between the sub-Saharan African and European individuals, and to a lesser extent, toward the Native American individuals (fig. 1*A*), in agreement with a process of extensive admixture in present Brazilian populations (Kehdy et al. 2015). Evidences of admixture in the present Brazilian samples can be also found in the analysis of their haplotype structure. The FineStructure dendrogram (fig. 1*A* and supplementary figs. S9–S13 and supplementary table S5, Supplementary Material online) obtained from the same individuals using the ChromoPainter coancestry matrix (supplementary fig. S12, Supplementary Material online) shows the individuals from Bambui, Pelotas, and Salvador clustered in mixed groups of similar admixture proportions from Native American, European, and sub-Saharan African ancestries. Some of these groups cluster together with sub-Saharan African or European clusters, but not with Native Americans, as none of the admixed Brazilian individuals have predominant Native American ancestry (fig. 1*A* and supplementary figs. S9–S13, Supplementary Material online). As previously shown (Kehdy et al. 2015), individuals from Salvador present, on average, higher amounts of sub-Saharan African ancestry than individuals from Pelotas and Bambui, although there is a high variability in the admixture proportions within each population. Native American ancestry is found at very low proportions in most of the analyzed individuals (supplementary fig. S14, Supplementary Material online).

In order to analyze and compare the substructure of the Native American component between the individuals from Bambui, Pelotas, and Salvador, we reconstructed, for each population, Native American individuals with rearranged chromosomes made of the Native American ancestry haplotypes of the admixed Brazilian individuals (extracted after RFMix [Maples et al. 2013] local ancestry analysis, see Materials and Methods and supplementary fig. S4, Supplementary Material online).

Despite the low proportion of the Native American component in admixed Brazilians (∼7% on average), we were able to reconstruct 45 diploid individuals for Bambui, 197 for Pelotas, and 51 for Salvador with full Native American ancestry. These reconstructed individuals do not show evidence of putative European or sub-Saharan African ancestries neither in and ADMIXTURE analysis (supplementary fig. S3, Supplementary Material online) nor in an f4 test of the form f4 (reconstructed Native American, Native American; European, sub-Saharan African) (supplementary figs. S15–S18, Supplementary Material online).

After the rearrangement of the chromosomes, the reconstructed Native American individuals cluster with other nonadmixed Native Americans and present a genetic structure correlated with continental geography. A PCA (fig. 1*B*) shows the reconstructed Native American individuals from Bambui, Pelotas, and Salvador grouped with current nonadmixed Native American individuals. The FineStructure results (fig. 1*B* and supplementary figs. S19–S23 and supplementary table S6, Supplementary Material online) obtained with the reconstructed individuals using the ChromoPainter coancestry matrix (supplementary fig. S22, Supplementary Material online) also show the reconstructed Native American individuals from Bambui, Pelotas, and Salvador within or next to reference nonadmixed Native Americans. PCA performed only with Native American samples shows that reconstructed individuals fit in the PCA space of the Native American diversity (supplementary figs. S24 and S25, Supplementary Material online). However, population specific genetic drift pulls each of the different principal components. Genetic drift also can affect other allele frequency-based analysis. Outgroup f3 values in the form of f3 (Reconstructed Individual, Native American; sub-Saharan African) do not allow enough resolution to discuss the genetic relationship between Native American populations (supplementary figs. S26 and S27, Supplementary Material online); for a given outgroup, per each reconstructed population, the Native American populations do not differ in the f3 values.

**Fig. 2.**
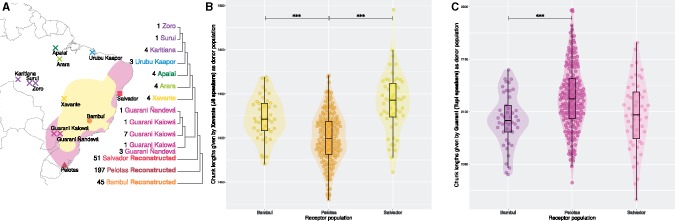
—(*A*) Geographical location of samples within Brazil current borders. Colored coastal regions according to Tupian-speaking populations (in pink) and non-Tupian- (mostly Jê) speaking populations (in yellow), at the time of European arrival (16th century) according to historical records (Soares de Souza 1879; Cardim 1925; Métraux 1927). FineStructure dendrogram of the sampled (crosses) and reconstructed Native American populations is shown. Dendrogram and geographical locations are colored according to the clusters from FineStructure: Tupi_Madeira (purple), Urubu_Kaapor (blue), Apalai (dark green), Arara (light green), Xavante (yellow), Guarani (pink), Salvador (light red), Pelotas (dark red), and Bambui (orange). (*B*, *C*) Differential ancestry between Bambui, Pelotas, and Salvador reconstructed populations as the total length of haplotypes shared with Native Americans playing as donor populations. Only the two donor clusters for which reconstructed individuals present significant differences between them are plotted: Xavante (*B*) and Guarani (*C*). Supplementary figures S33–S35 and supplementary tables S6–S8, Supplementary Material online, show additional comparisons.

Therefore, in order to recover the population structure hidden in the reconstructed Native American individuals and overcome the limitations of the allele frequency-based methods, we use more-sensitive haplotype-based methods. The FineStructure dendrogram built with Native American samples (fig. 2*A* and supplementary figs. S28–S32, Supplementary Material online) reveals the substructure of the Native American individuals, which cluster according to their population label, including the reconstructed Native Americans from Bambui, Pelotas, and Salvador. The dendrogram presents consistent clusters through different seeds: Tupi-Madeira (which includes the Tupi-speaking populations from the Madeira river basin: Karitiana, Surui and Zoro), Urubu-Kaapor, Apalai, Arara, Xavante, Guarani (which includes both Guarani Kaiowá and Guarani Ñandevá populations), Salvador, Pelotas, and Bambui. Interestingly, different seeds of FineStructure (supplementary figs. S28–S30, Supplementary Material online) show Urubu-Kaapor cluster related both to Tupi-Madeira and Apalai clusters, and according to ChromoPainter coancestry matrix and PCA computed from this coancestry matrix (supplementary figs. S31 and S32, Supplementary Material online), Urubu-Kaapor cluster shares haplotypes with both populations. This suggests admixture of the Urubu-Kaapor population (Tupi-speaking from the Northern branch of the Tupi-Guarani family) with Apalai, a Carib-speaking group, or a close population. The six clusters of current nonadmixed Native American populations were used in the subsequent analyses as a set of donor populations in ChromoPainter to explore the differential ancestry between the reconstructed populations from Bambui, Pelotas, and Salvador.

### Differential Native American Ancestry in the Reconstructed Individuals

Haplotype-based methods, like ChromoPainter, can mitigate and partially overcome the effect of genetic drift (van Dorp et al. 2015; Lawson et al. 2018) and allow us to see differential ancestry patterns between populations due to either differential admixture history or different origins. We assumed that some but limited population movements during the colonization process may have occurred. Therefore, we based our scheme on analyzing the differential ancestry of Native American ancestral populations between the three reconstructed populations to reveal asymmetric genetic histories, instead of looking for the total proportions of each ancestry in each reconstructed population. Native American reconstructed populations from Bambui, Pelotas, and Salvador differ in the total length of haplotypes they receive from Native American populations that play as donor populations (supplementary fig. S33, Supplementary Material online). The reconstructed Native American populations show significant differences among them in the total length of haplotypes they receive from the Xavante (Jê speakers) and Guarani (Tupi speakers from the Tupi-Guarani family) clusters (fig. 2 and supplementary tables S7–S10, Supplementary Material online). Both reconstructed Native Americans from Bambui (Brazilian Central Plateau) and Salvador (Northeast Coast) receive more haplotypes from Xavante (Jê speakers) than the reconstructed Native Americans from Pelotas (South Coast). However, no significant differences are observed between Bambui and Salvador (fig. 2*B*). Additionally, reconstructed Native Americans from Pelotas share more haplotypes with Guarani (Tupi speakers) than the reconstructed Native Americans from Bambui, whereas no significant differences are observed between Salvador and Bambui or between Salvador and Pelotas (fig. 2*C*).

In the ChromoPainter analyses, each recipient individual is an independent run, which enables the comparisons between recipient populations as described above. However, the genome of a recipient individual has a given total length, and then the increase in the total length of the haplotypes shared with a donor population (i.e., Xavante) can produce the decrease of the total length of the haplotypes shared with another donor population (i.e., Guarani). To discard artifacts, we thus repeated the previous analyses with all Native American clusters as donor populations excluding the Guarani and, in a parallel, we did the same analyses excluding the Xavante. This allowed us to see if the signal detected from one of the two populations was artifactual and caused by the true signal of the other. In the first case, when excluding Guarani of the analysis, the signal from Xavante persists and the significant differences observed in the previous analyses are still significant. To compensate the absence of Guarani as a donor, a new significant difference appears from another Tupi population: Tupi-Madeira gives more haplotypes to Pelotas than to Salvador (supplementary fig. S34 and supplementary table S8, Supplementary Material online). However, when excluding Xavante of the analysis, no significant differences are observed and the Guarani difference between Pelotas and Salvador is no longer significant (supplementary fig. S35 and supplementary table S9, Supplementary Material online). This result points to a dual ancestry Jê/Tupi in the reconstructed individuals, where the strongest signal is a nonhomogeneous Jê ancestry between the reconstructed Native American populations.

### Estimated Effective Population Sizes in the Reconstructed Individuals

**Fig. 3.**
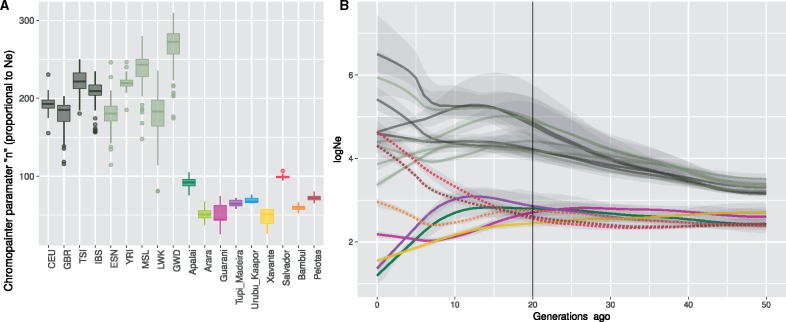
—Effective population sizes (Ne). (*A*) *n* parameter of ChromoPainter after 15 iterations of the Expectation Maximization algorithm. (*B*) Log(Ne) obtained from IBD fragments with IBDseq and IBDNe from present to 50 generations ago filtered by a 95% confidence interval range of 2.5. Dashed and solid lines correspond to the reconstructed and reference populations, respectively. Colors are the same as in (*A*).

The reconstructed individuals allowed us to estimate the effective population size of the putative ancestral Native American populations. Genetically reconstructed Native American populations present effective population sizes similar to the other nonadmixed Native Americans (fig. 3 and supplementary figs. S36 and S37, Supplementary Material online). Both the *n* parameter from ChromoPainter (fig. 3*A*), related to effective population size, and the effective population size computed by IBDNe (fig. 3*B*) show low values for reconstructed Native American populations, which overlap with the values estimated for the present Native American populations (similar results are found using Refined IBD, supplementary figs. S38–S40, Supplementary Material online). Both analyses also show clear differences between Native American and non-Native American populations. Within the reconstructed Native American populations, Salvador shows the highest effective population size, followed by Pelotas, whereas Bambui presents the lowest effective population size.

The availability of the reconstructed Native American samples allowed us to estimate both the genetic diversity and the changes of the effective population size through time. Interestingly, reconstructed Native Americans have similar but significantly higher genetic diversity values than the rest of current Native Americans (supplementary tables S11 and S12, Supplementary Material online). When analyzing the evolution of the effective population size in the last 50 generations through IBDNe analysis, Native American populations, except for the reconstructed samples, went through a dramatic fall of their effective population sizes after the contact with European conquerors/colonizers (fig. 3*B*). The vertical line in figure 3*B* corresponds approximately to 1,500 CE, the year of the arrival of the Portuguese navigator Pedro Álvares Cabral and his commanders in what today is Brazil’s coastline. Guarani and Xavante populations show a decline of effective population size coincident with the arrival of the Europeans, whereas in the Tupi-Madeira and Apalai populations also show a decline in effective population size, but with a starting point set around ten generations after, in accordance to a later European contact of these populations (supplementary table S13, Supplementary Material online). Before 1500 CE, reconstructed Native American populations present similar effective population sizes to the current nonadmixed Native American populations. However, after 1500 CE, they do not show a decline in their effective population sizes and, in contrast, an increase of the effective population size during the last generations is observed. Bambui population shows an initial slight decline in population size that recovers around seven generations ago. Guarani also stop the population size fall around this date. Admixed American populations (CLM, PUR, MXL, and PEL) show effective population size curves that fluctuate between the estimations of the European and African populations and those of Native American populations (supplementary fig. S37, Supplementary Material online). Interestingly PEL (Peruvians from Lima, Peru) show a pattern more similar to the reconstructed populations, starting with values similar to Native Americans and growing after 1500 CE.

## Discussion

The reconstruction of the Native American component from Native American ancestry haplotypes of admixed urban Brazilian populations genetically recovers ancient Native American populations that inhabited these areas centuries ago. Most of the populations that lived in nowadays Brazilian south-eastern coast and the Brazilian Central Plateau experienced either a population contraction or admixed to urban populations short after the arrival of the Europeans, in 1500 CE (Kehdy et al. 2015). The study of reconstructed Native American populations from North-East, South-East, and South of Brazil shed light on the pre-Columbian genetic history and the population structure of this subcontinental region. The genetic reconstruction of Native American populations that no longer exist as nonadmixed populations opens the possibility to recover lost chapters of South American history and to reveal, at least genetically, who were the people who used to live in this land before the arrival of Europeans.

Several genetic studies have described a Native American genetic scenario where language and genetics structure do not often correlate, with low genetic diversity compared with non-Native American populations, and high heterogeneity between populations (Amorim et al. 2013; Ramallo et al. 2013). This scenario has been associated with the presence of extensive genetic drift after a series of bottlenecks and population splits after the peopling of the continent, resulting in low effective population size of these Native American groups (Hey et al. 2005; Gravel et al. 2013; Fagundes et al. 2018). Allele frequency-based methods, such as clustering methods (for instance, ADMIXTURE), PCAs, and outgroup f3–f4 statistics are not able to refine genetic relationships between populations when these groups suffered from extensive genetic drift. The use of haplotype-based methods, which are less affected by genetic drift (van Dorp et al. 2015; Lawson et al. 2018), reveals a finer resolution in the structure of Native American populations where population groups have higher affinity with linguistic classifications. In our analysis, different Tupi-speaking populations from the right margin tributaries of the Amazonas are genetically related; and they are also related to Urubu-Kaapor, a Tupi-speaking population from the Northern branch of the Tupi-Guarani family from Maranhão state, but with its origins in the basins of the rivers Xingu and Tocantins (de Almeida and Neves 2015). Guarani populations (Tupi-speaking populations from a southern Tupi-Guarani branch), Carib- and Jê-speaking populations configure linguistic population groups that also cluster genetically. In this general scenario where the main linguistic groups correlate with the main genetic components, we observe a population structure shaped by the dichotomy Tupi versus Jê ancestry in the Brazilian Plateau and the South-Eastern Coast, through the reconstructed populations (fig. 2 and supplementary figs. S33–S35 and supplementary tables S7–S9, Supplementary Material online).

Historical records describe a landscape where the population distribution at the arrival of the Europeans was divided between the Brazilian Central Plateau and the coast (Soares de Souza 1879; Cardim 1925). On one side, the hinterlands were mainly populated by non-Tupian populations named *Tapuia* by Tupi populations and European sources, where we find mainly Macro-Jê speaking populations like Botocudo/Aimoré or Pataxó in the hilly areas nearby the coast, and Jê speaking populations like Xavante or Xerente more inland (Métraux 1927; Steward 1948; Carneiro da Cunha 1998). On the other side, both the East coastline strip on one hand and the basins of the rivers Paraná, Paraguay, and Uruguay and the South coast on the other hand were mainly populated by Tupi-speaking populations from the Tupi-Guarani family, like Tupinambá and Guarani, respectively (Soares de Souza 1879; Cardim 1925; Métraux 1927; Carneiro da Cunha 1998). However, the exact borders of these territories are not clear, and Macro-Jê populations could have broken the Tupi coastline continuum and inhabited some coastal regions (Soares de Souza 1879). According to the historical and linguistic records, Bambui, located in the Brazilian Central Plateau, is in a geographical area thought to be occupied mainly by Jê or other non-Tupi populations; whereas Pelotas, in the South coast, and Salvador, in the East coast, are in a region where more Tupi ancestry is expected (Steward 1948; Campbell and Grondona 2012).

The differential ancestry of the Tupi (Guaraní) and Jê (Xavante) components shapes the structure of the three reconstructed populations. Our results show higher Jê ancestry in Bambui and lower in Pelotas, compensated with, respectively, lower and higher Tupi ancestry, particularly Guaraní. Unexpectedly, the reconstructed Native Americans from Salvador have more Jê ancestry than the reconstructed Native Americans from Pelotas, but they do not show significant differences in the amount of Tupi ancestry. We hypothesize that the observed higher Jê ancestry in Salvador could be a wider signal related to Macro-Jê populations, which were populations closer to Salvador. This reinforces the idea of Salvador as a crossroad of both components and challenges the thought of a long continuum of a large coastal population of Tupi ancestry. Alternatively, a Macro-Jê Native American ancestral component could have been introduced from neighboring hinterland areas during the configuration of the admixed population of Salvador. However, the original approximate Bayesian computation (ABC) approach performed with the present samples showed that the Native American component was mostly introduced in the admixed populations of Bambui, Pelotas, and Salvador soon after the arrival of the Europeans to the Brazilian coast (Kehdy et al. 2015) suggesting that recent migrations might have had limited impact in the Native American composition of admixed urban groups. This ABC approach was based on the observed and simulated lengths distributions of chromosome segments of continuous specific ancestry (CSSA) for different admixture dynamics for each ancestral population at three migration pulses (early, intermediate and recent).

The effective population sizes of the reconstructed Native American populations are similar to other Native American groups and lower than non-Native American groups. Very recently, ancestry specific estimations for effective population sizes through IBDNe have been successfully analyzed in admixed American populations comparing ancestry specific effective population size evolution along time within the same population through IBD, which allows a fine evaluation of the effective population size in recent generations (Browning and Browning 2015; Browning et al. 2018). In this sense, Browning et al. (2018) alert about estimating effective population sizes on the ancestral continental genetic components based on local ancestry in American admixed populations after the admixture process. In addition, caution should be taken when comparing population sizes of reconstructed populations using IBDNe because these reconstructed groups do not represent biological entities and might be affected by the reconstruction process. Nonetheless, the estimation of the effective population sizes through IBDNe in the reconstructed individuals might reveal some aspects of the demography of the Native American groups before the admixture.

Here, we have contrasted the effective population size of the reconstructed Native American populations with other Native American populations. We observe that they show a similar behavior to other Native American populations in pre-Columbian times (until 1500 CE). At this point Native American populations, except the reconstructed groups, show a decline in their effective population size. This abrupt fall in the population size might be associated with the demographic impact suffered by Native American groups after the arrival of the Europeans, either as direct causatives or as a source for a shift of the pathogenic environment to which Native American populations had adapted. Amazonian populations experience this fall some generations after than southern populations, coincident with their later contact time with European populations (fig. 3*B* and supplementary table S13, Supplementary Material online).

Interestingly, Guarani populations are an exception within Native Americans and stop their population size decline 12 generations ago. We also observe mixed genetic clusters from Guarani Kaiowá and Ñandevá in the FineStructure analysis, indicating a possible more recent admixture process of these groups. Both observations could be a signal of the demographic effect caused by the expulsion of the Jesuits and the end of the Guarani Jesuit reductions at the 17th century, where other Guarani peoples had been forced to the integrate to the European culture and to adopt the catholic faith. This major historical event caused the relocation of the Guarani people, to their actual neighbor locations in Paraguay, Northern Argentina, and Northern, Southeastern, and Midwestern states of Brazil (Ferreira Thomaz de Almeida and Mura 2003).

In contrast, the reconstructed Native American populations do not show signals of effective population size decrease and they even experienced an increase after this period. These results could suggest that admixed Brazilians might have acted as a reservoir of the diversity present before 1500 CE in each of the geographical locations analyzed. However, there are several factors that may have affected the effective population size estimates of the reconstructed populations after the date of the admixture. Regarding the methodology, the main possible source of bias relies on the fact that the amount of individuals in identity by descent in a certain genomic window could eventually increase as an artifact of splitting the window region from a single real individual to various fragments that end up in different reconstructed individuals (see also kinship analyses in supplementary table S14, Supplementary Material online). Beyond possible methodological sources of bias, diverse events in the genetic history of the ancestral Native American populations could lead to the observed results. First, the recent increase of the effective population size may be the result of recent population growth of the Native American ancestral component within the growing admixed populations. Recent studies have analyzed through IBDNe how recent population growths after strong bottlenecks have affected the admixed American populations (Browning et al. 2018; Mooney et al. 2018). Browning et al. depicted how the effective population size of the split ancestral components mirror the increase of the effective population size of the whole genomes of the admixed population after the admixture event. Mooney et al. showed that the high values of runs of homozygosity (ROH) observed in admixed American populations cannot only be explained by a population size bottleneck but also by a consanguineous non-random mating pattern. They also stated that IBDNe estimates may not be as reliable when applied to small sample sizes. Alternatively, the observed increase of the effective population size could also be due to the admixture of different sources of Native American ancestral populations surrounding the sample location at the time of the emergence of the admixed population. Therefore, although ancient population size estimations present higher confidence, caution should be taken when interpreting the effective population sizes of the reconstructed populations after the colonization process started.

Looking for other evidences of the eventual genetic reservoir of the Native American diversity in the admixed populations, we found that the genetic diversity is slightly but significantly higher in all reconstructed populations than in all Native American populations (supplementary tables S11 and S12, Supplementary Material online). In contrast with the IBDNe estimates, the allele based genetic diversity is not affected by possible methodological bias sources that may arise from the reconstruction of individuals. However, they are affected by an eventual not enough representative sampling of the reference Native American populations. These results are consistent with higher Native American effective populations size estimates obtained from mtDNA from admixed populations than from current Native American populations (Tavares et al. 2019) or higher variability in ancient DNA than in current samples (Llamas et al. 2016) pointing to a high reduction of the Native American diversity that could have partially be saved in admixed populations.

In conclusion, this study sheds light on the history and genetics of Native Americans of Brazil before European colonization, after which the Native American populations either admixed with European and sub-Saharan African populations or experienced a strong decrease of their genetic diversity, as our results show. We have been able to reconstruct the ancient Native American populations inhabiting Eastern Brazil before the arrival of the Europeans, starting from the Native American component of the current admixed Brazilian populations (on average the 7%), in a region where most Native American populations no longer exist as nonadmixed populations. The genetic structure of the reconstructed Native American populations reflects a dichotomic ancestry related to Tupi- and Jê-speaking populations and suggests the contact between these Native American groups in the east coast of Brazil in precolonial times. In addition, we track the decay of the effective population size of Native American populations due to the advance of the colonization process. Similarly, we find weak but significant signals that point to the existence of a genetic reservoir of Native American diversity in the admixed populations of Brazil, although our contribution to corroborate this hypothesis is limited. This approach opens the door for the study of other ancestral populations that have experienced a similar population reduction without requiring the availability of ancient DNA. Further studies would clarify the demographic fluctuations of the populations described in this study, expand the population structure characterization detailed here, and contribute to the knowledge of the demographic history of South America before 1500 CE.

## Supplementary Material

Supplementary data are available at *Genome Biology and Evolution* online.

## Supplementary Material

evz161_Supplementary_Data
