# Supplementary material for: Reconstructed Lost Native American Populations from Eastern Brazil Are Shaped by Differential Jê/Tupi Ancestry
Source: Genome Biol Evol. 2019 Jul 22;11(9):2593–604. doi: 10.1093/gbe/evz161 (PMC6756188; doi:10.1093/gbe/evz161)
Supplement: evz161_Supplementary_Data [file evz161_supplementary_data.pdf]

Supplementary Figures

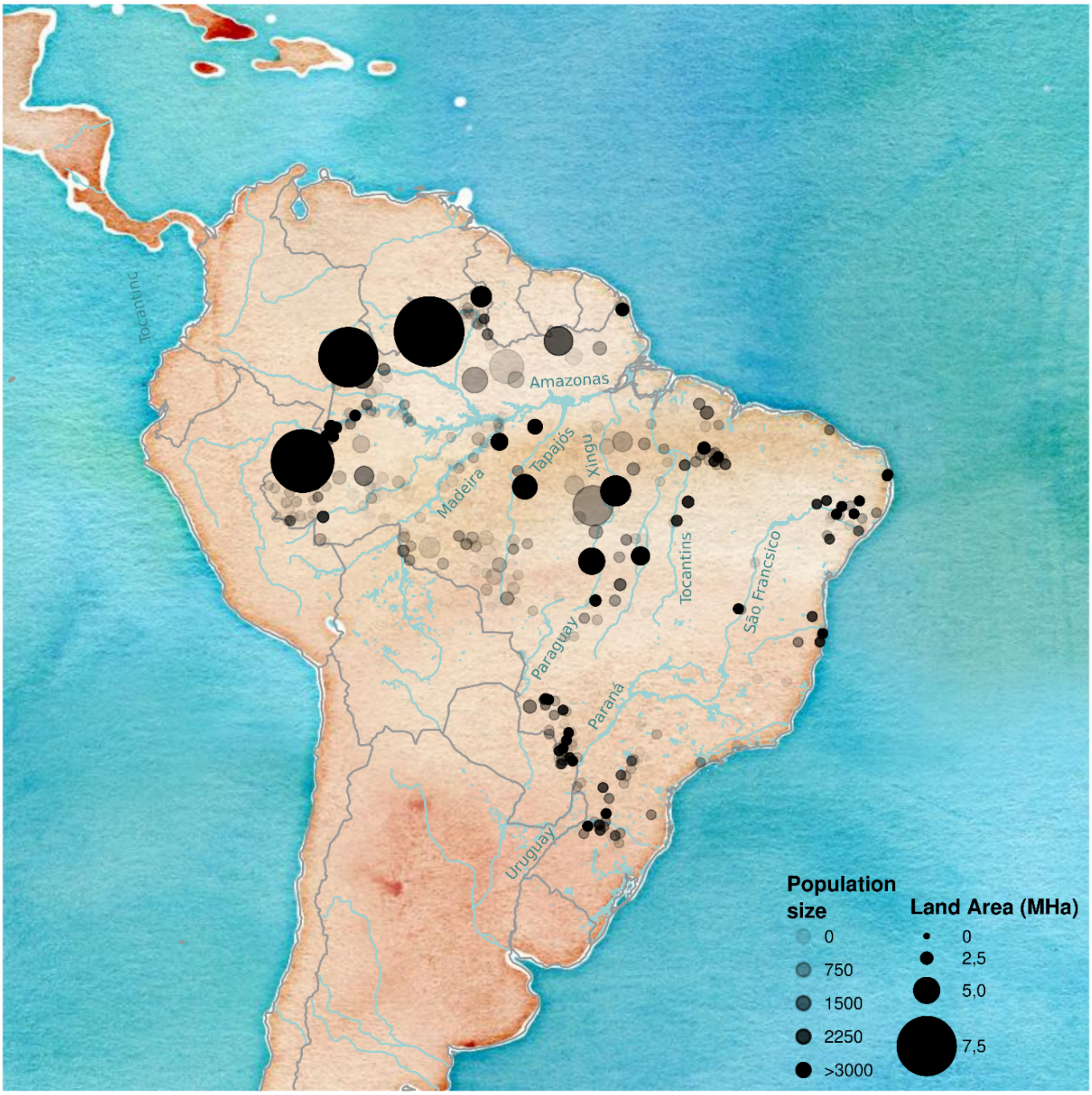

Figure S1. Native American lands location, area and population size data. Source: FUNAI and Censo Demográfico 2010 (IBGE). Land area is proportional to the size and the population size inversely proportional to the transparency of each point.

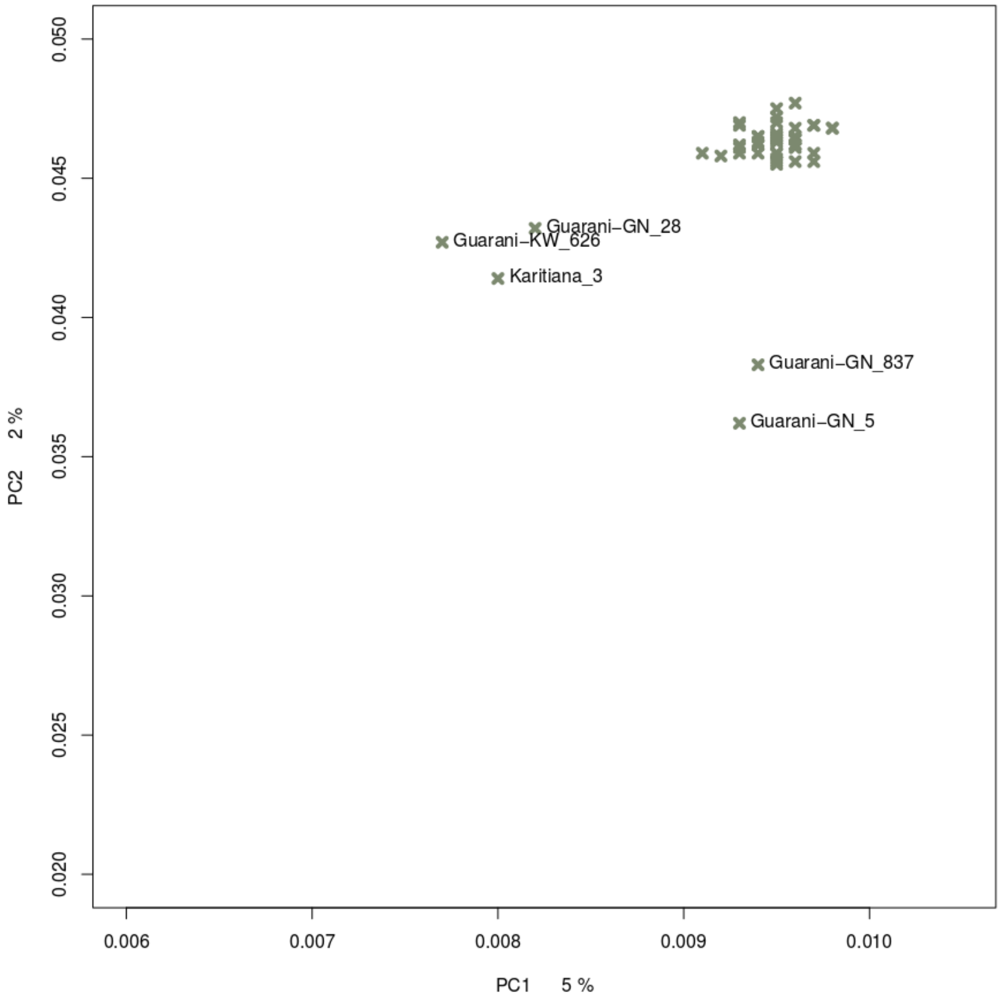

Figure S2. **Removed individuals.** Zoom of the PCA of the Figure1A. The samples labelled are the individuals removed because they show a clear shift towards sub-Saharan African and European individuals, suggesting some non-Native American admixture

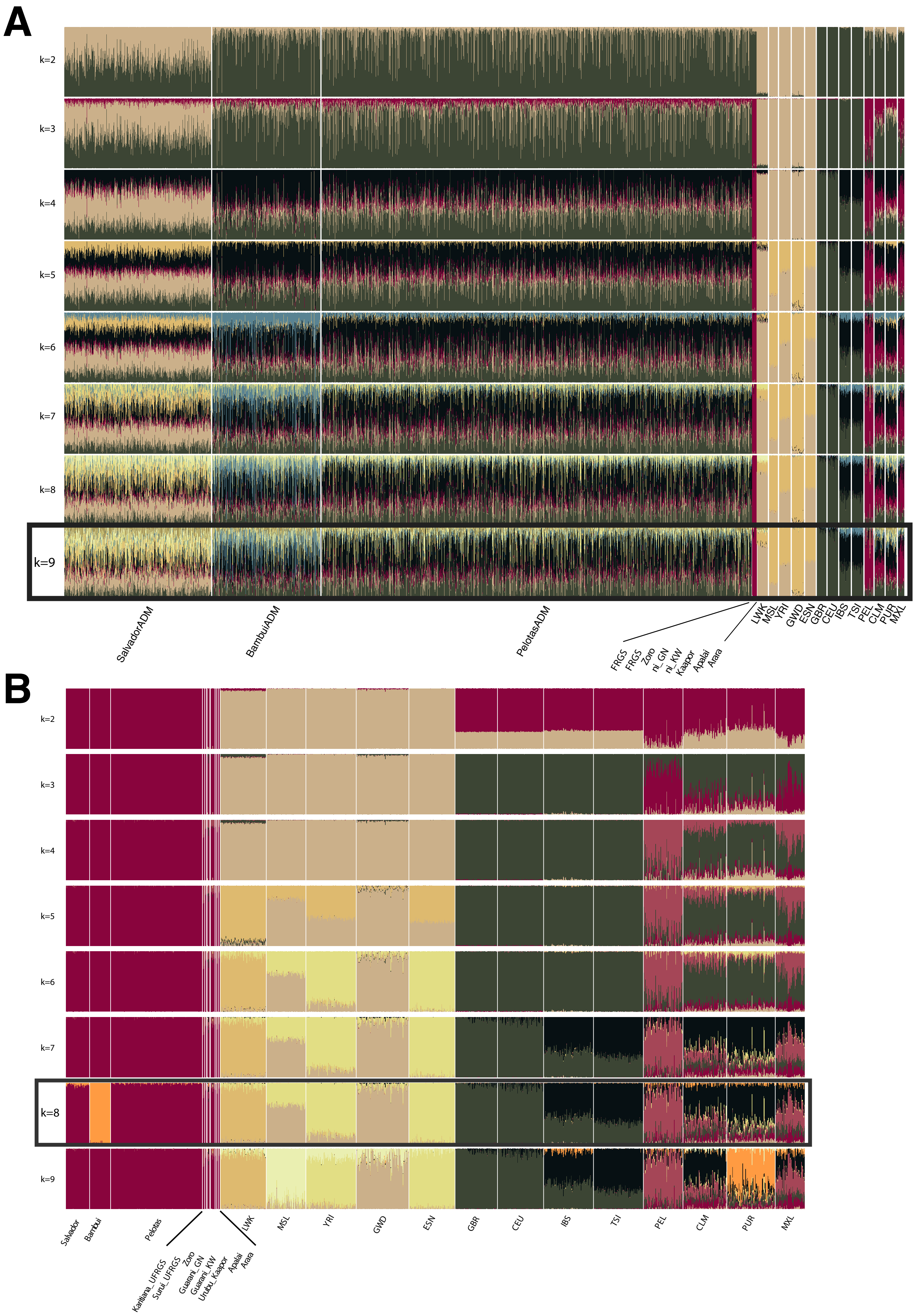

Figure S3. ADMIXTURE. ADMIXTURE plot for k=3 to k =9 before (A) and after (B) the reconstruction. The the k with lowest cross validation error is highlighted.

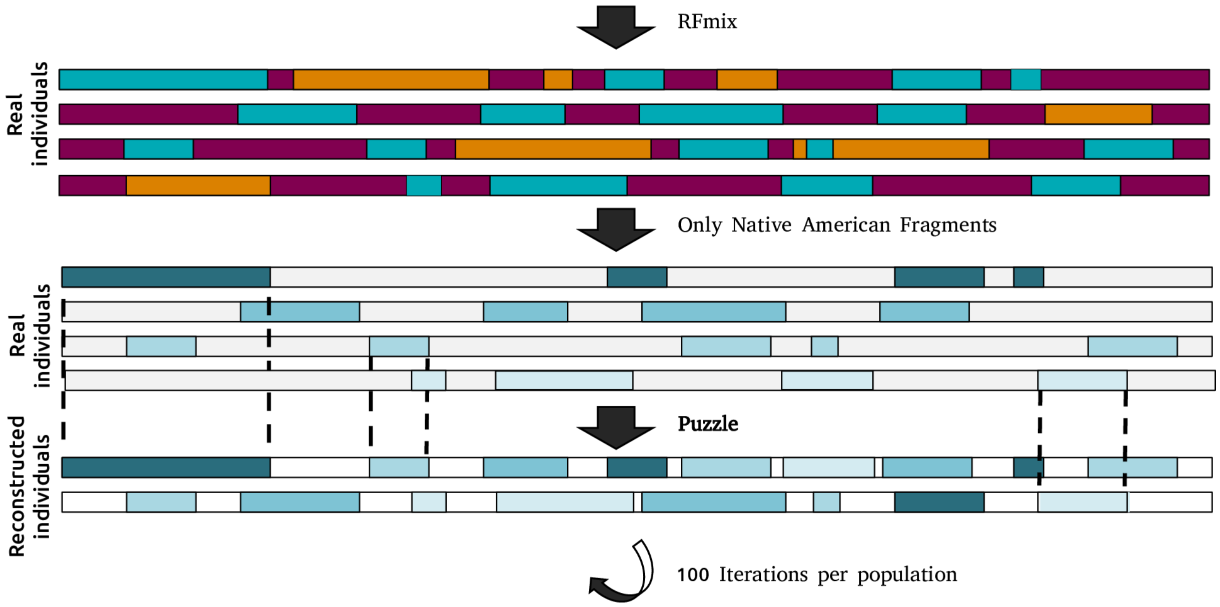

Figure S4. Reconstruction of Native American individuals. The diagram shows the local ancestry analysis and the jigsaw puzzle made with the Native American haplotypes. In order to reconstruct a chromosome, haplotypes are selected from the pool of Native American ancestry fragments within the population. The fragments to reconstruct a chromosome are chosen in order to minimize the gaps between fragments without overlapping them, using each fragment only once.

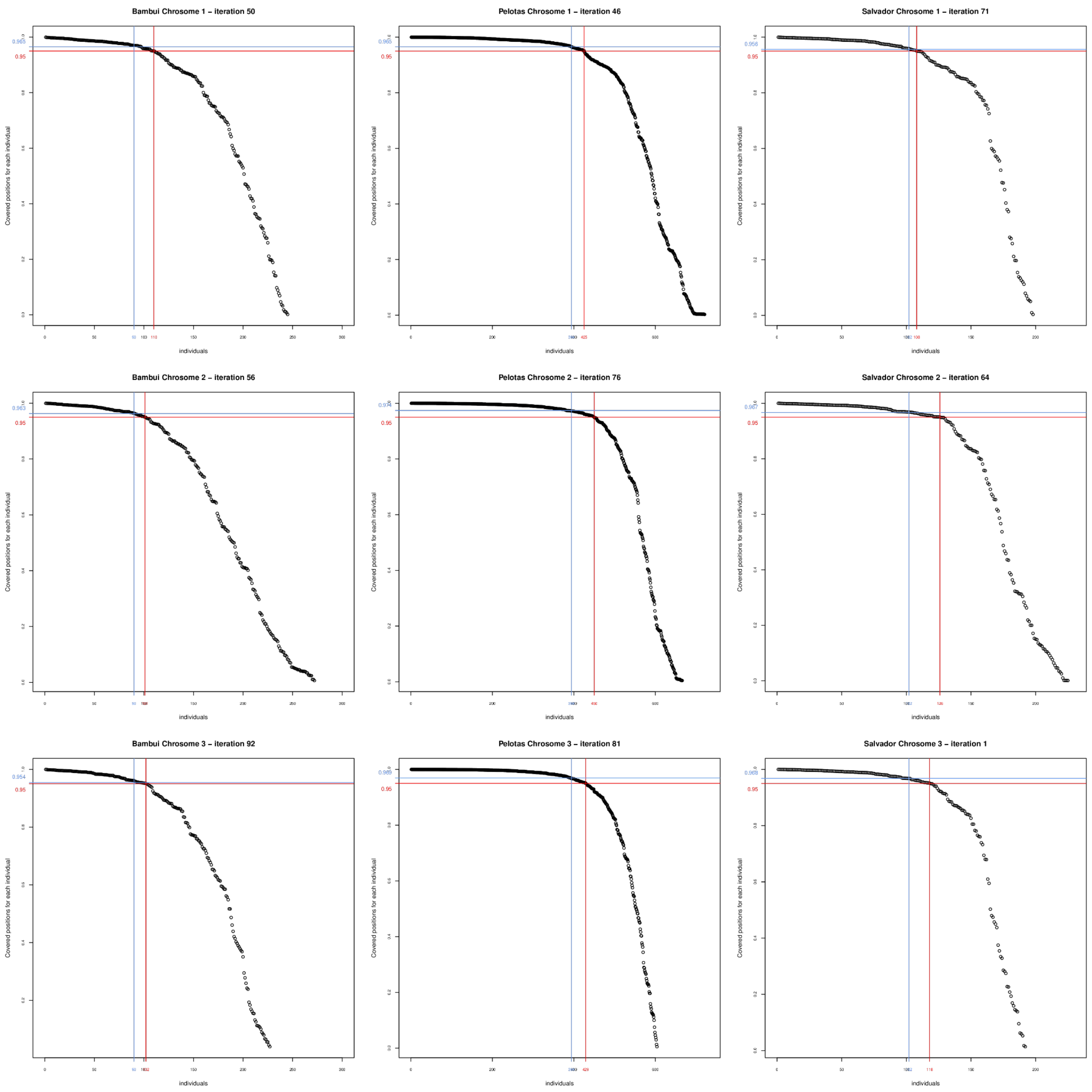
Bambui Chrosome 1 – iteration 50
Pelotas Chrosome 1 – iteration 46
Salvador Chrosome 1 – iteration 71
Bambui Chrosome 2 – iteration 56
Pelotas Chrosome 2 – iteration 76
Salvador Chrosome 2 – iteration 64
Bambui Chrosome 3 – iteration 92
Pelotas Chrosome 3 – iteration 81
Salvador Chrosome 3 – iteration 1
Covered positions for each individual
individuals
0.95

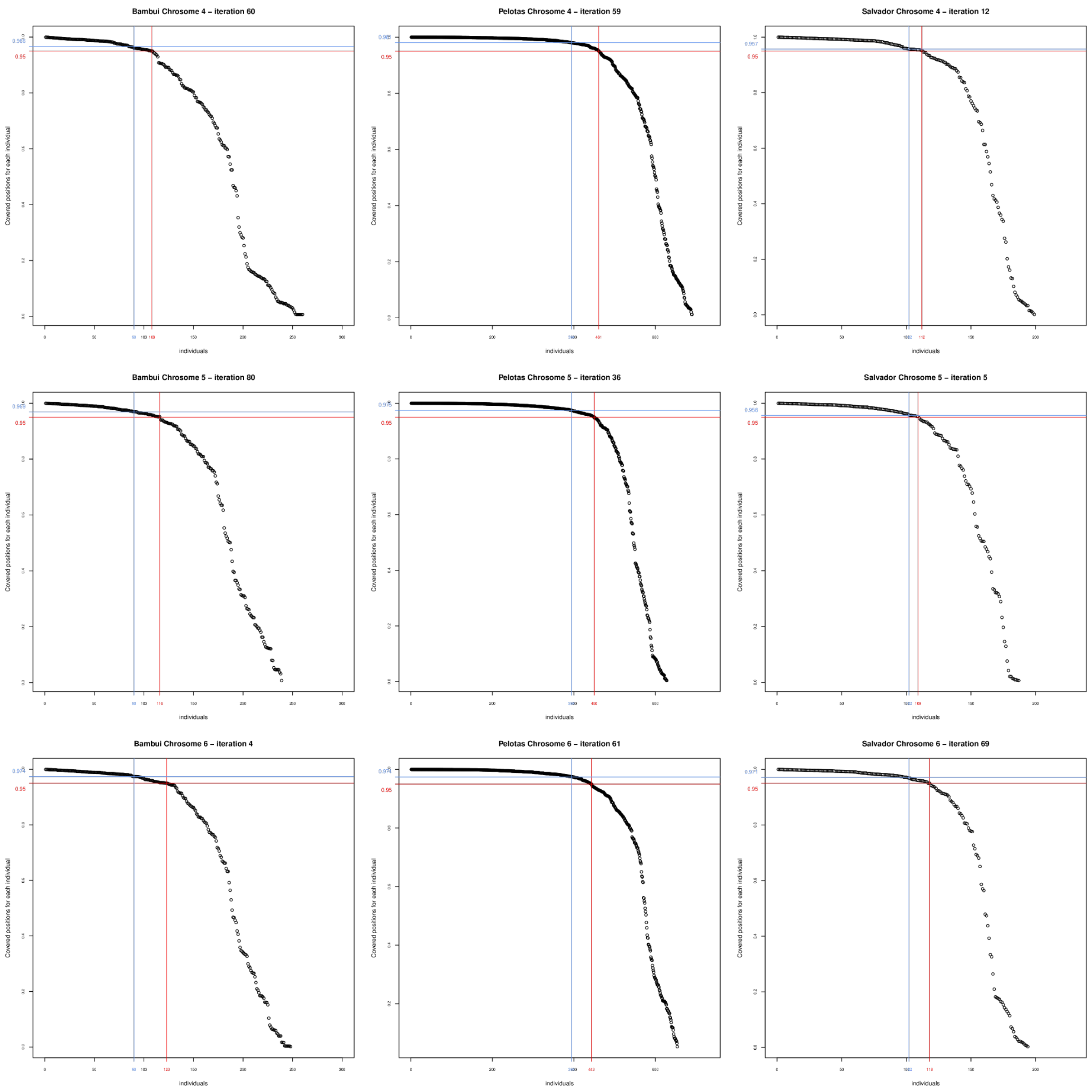
Bambui Chrosome 4 – iteration 60
Pelotas Chrosome 4 – iteration 59
Salvador Chrosome 4 – iteration 12
Bambui Chrosome 5 – iteration 80
Pelotas Chrosome 5 – iteration 36
Salvador Chrosome 5 – iteration 5
Bambui Chrosome 6 – iteration 4
Pelotas Chrosome 6 – iteration 61
Salvador Chrosome 6 – iteration 69
Covered positions for each individual
individuals
0.95

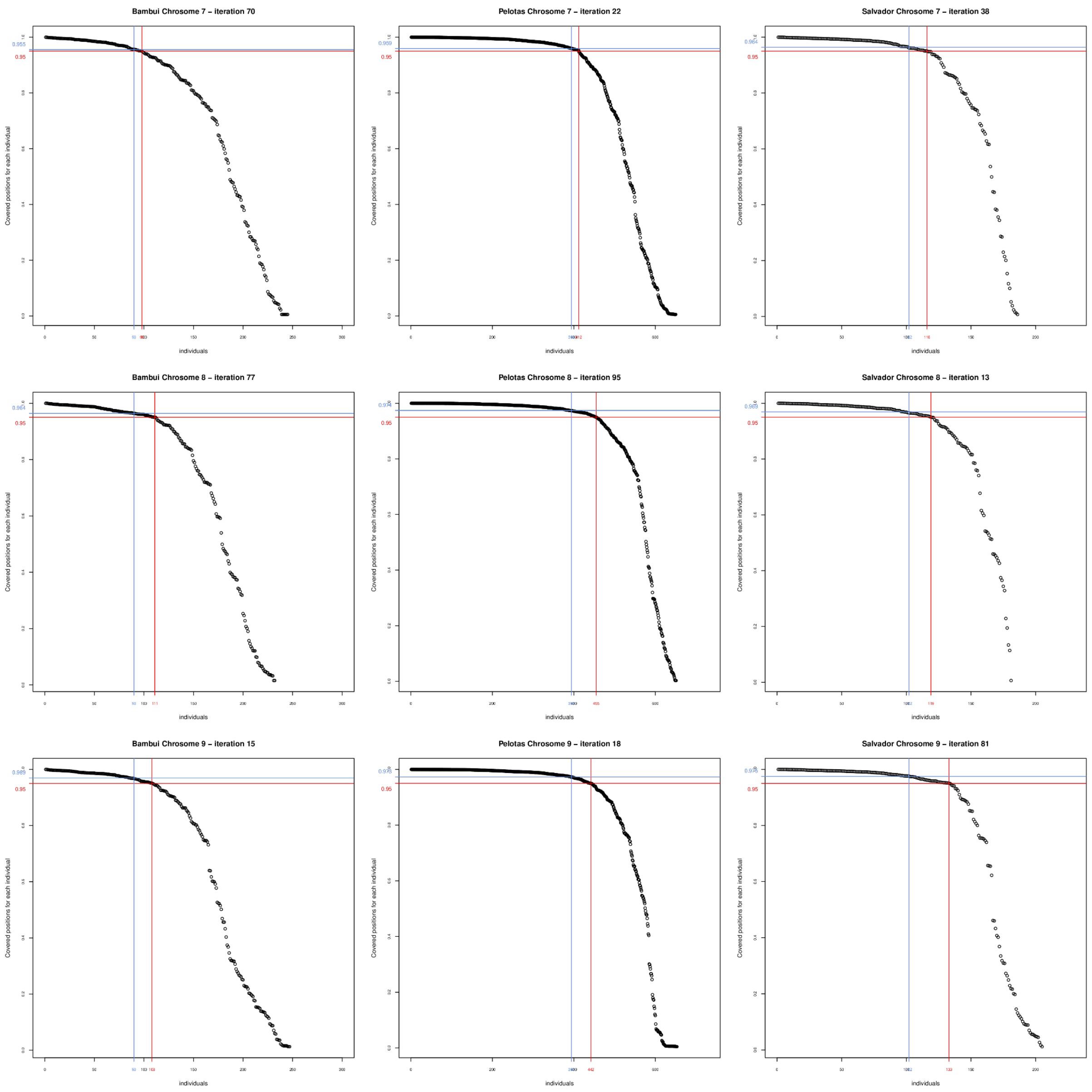
Bambui Chrosome 7 – iteration 70
Pelotas Chrosome 7 – iteration 22
Salvador Chrosome 7 – iteration 38
Bambui Chrosome 8 – iteration 77
Pelotas Chrosome 8 – iteration 95
Salvador Chrosome 8 – iteration 13
Bambui Chrosome 9 – iteration 15
Pelotas Chrosome 9 – iteration 18
Salvador Chrosome 9 – iteration 81
Covered positions for each individual
individuals
0.95

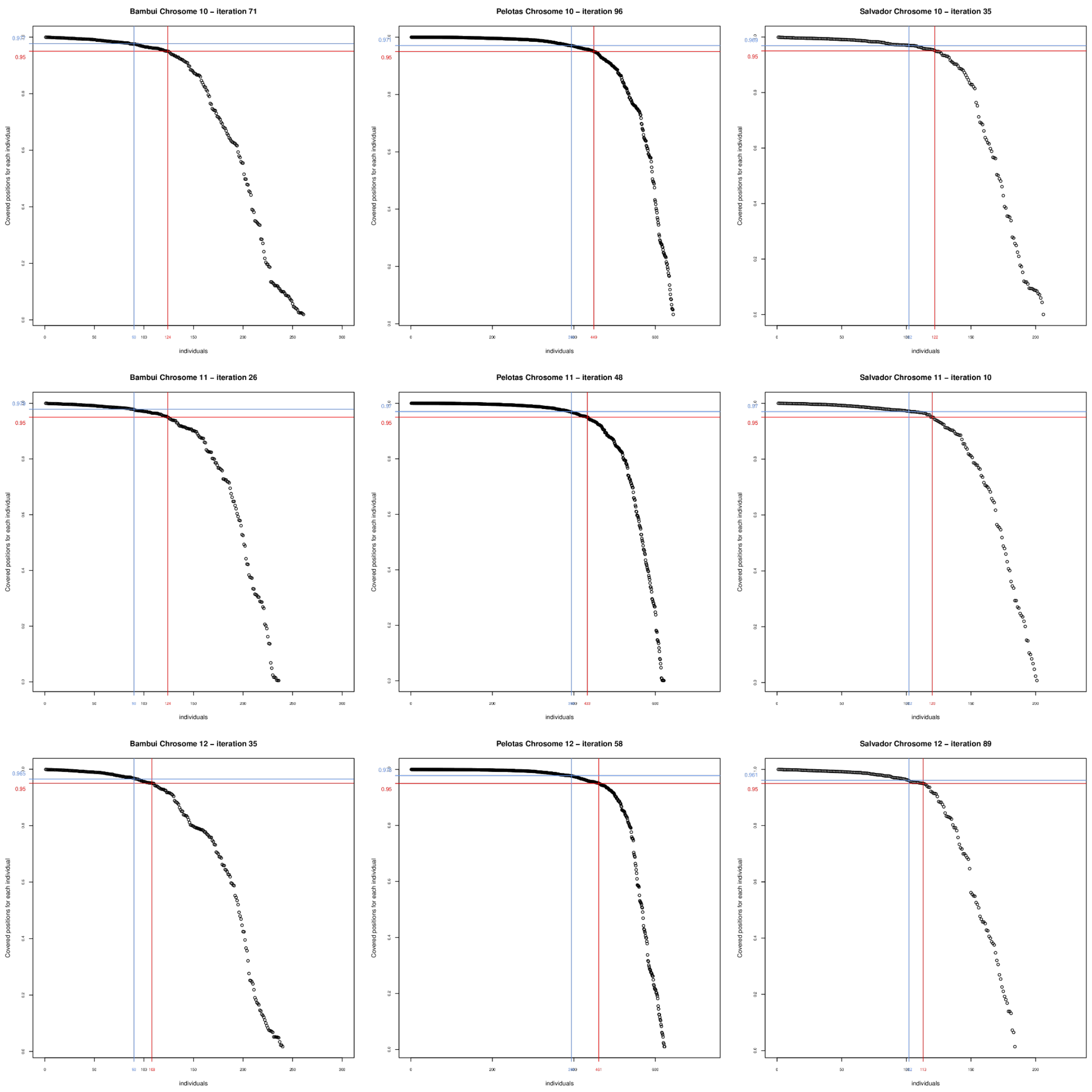

Bambui Chrosome 10 – iteration 71
Pelotas Chrosome 10 – iteration 96
Salvador Chrosome 10 – iteration 35
Bambui Chrosome 11 – iteration 26
Pelotas Chrosome 11 – iteration 48
Salvador Chrosome 11 – iteration 10
Bambui Chrosome 12 – iteration 35
Pelotas Chrosome 12 – iteration 58
Salvador Chrosome 12 – iteration 89
Covered positions for each individual
individuals
0.95

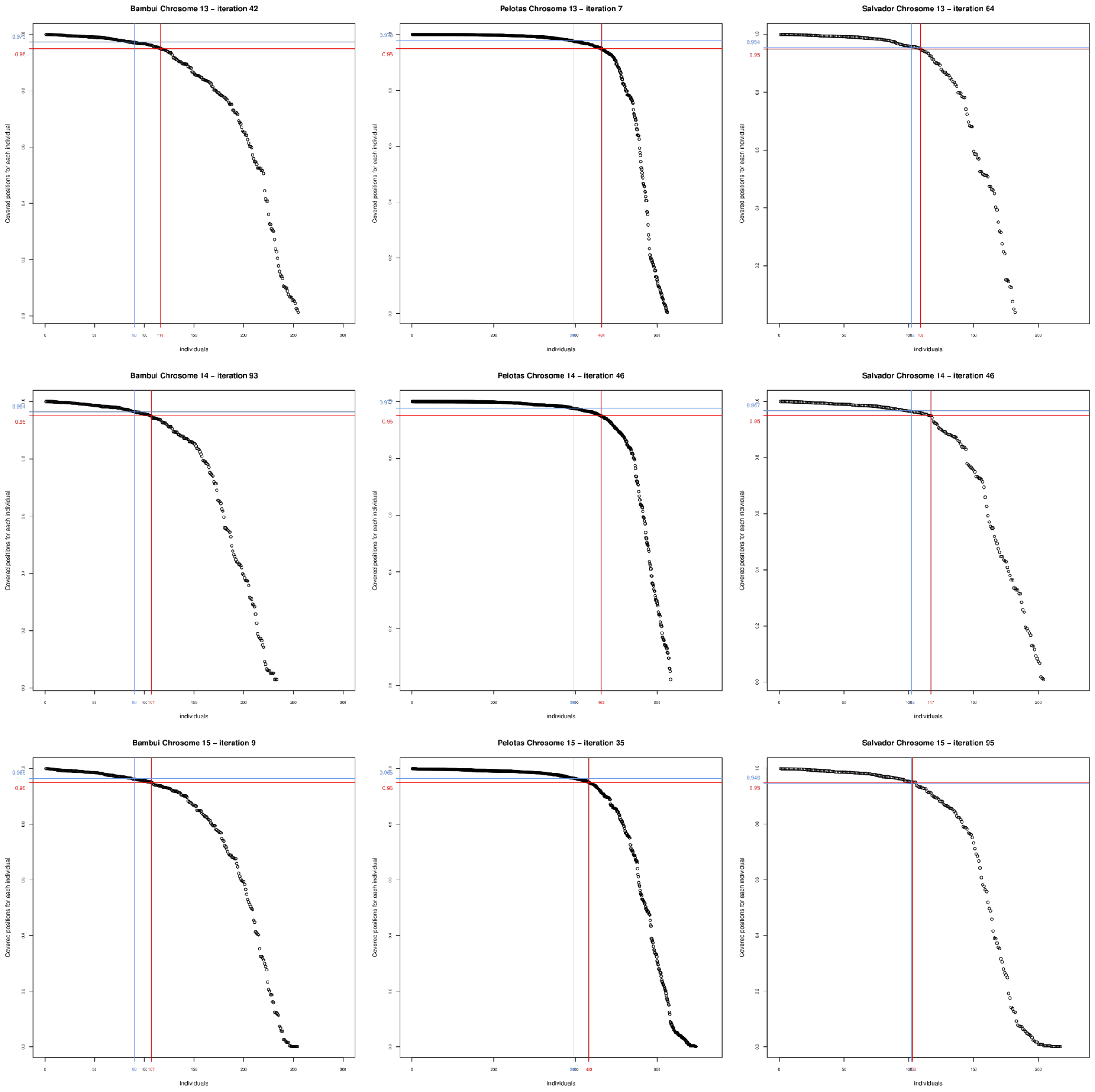
Bambui Chrosome 13 – iteration 42
Pelotas Chrosome 13 – iteration 7
Salvador Chrosome 13 – iteration 64
Bambui Chrosome 14 – iteration 93
Pelotas Chrosome 14 – iteration 46
Salvador Chrosome 14 – iteration 46
Bambui Chrosome 15 – iteration 9
Pelotas Chrosome 15 – iteration 35
Salvador Chrosome 15 – iteration 95
Covered positions for each individual
individuals
0.95

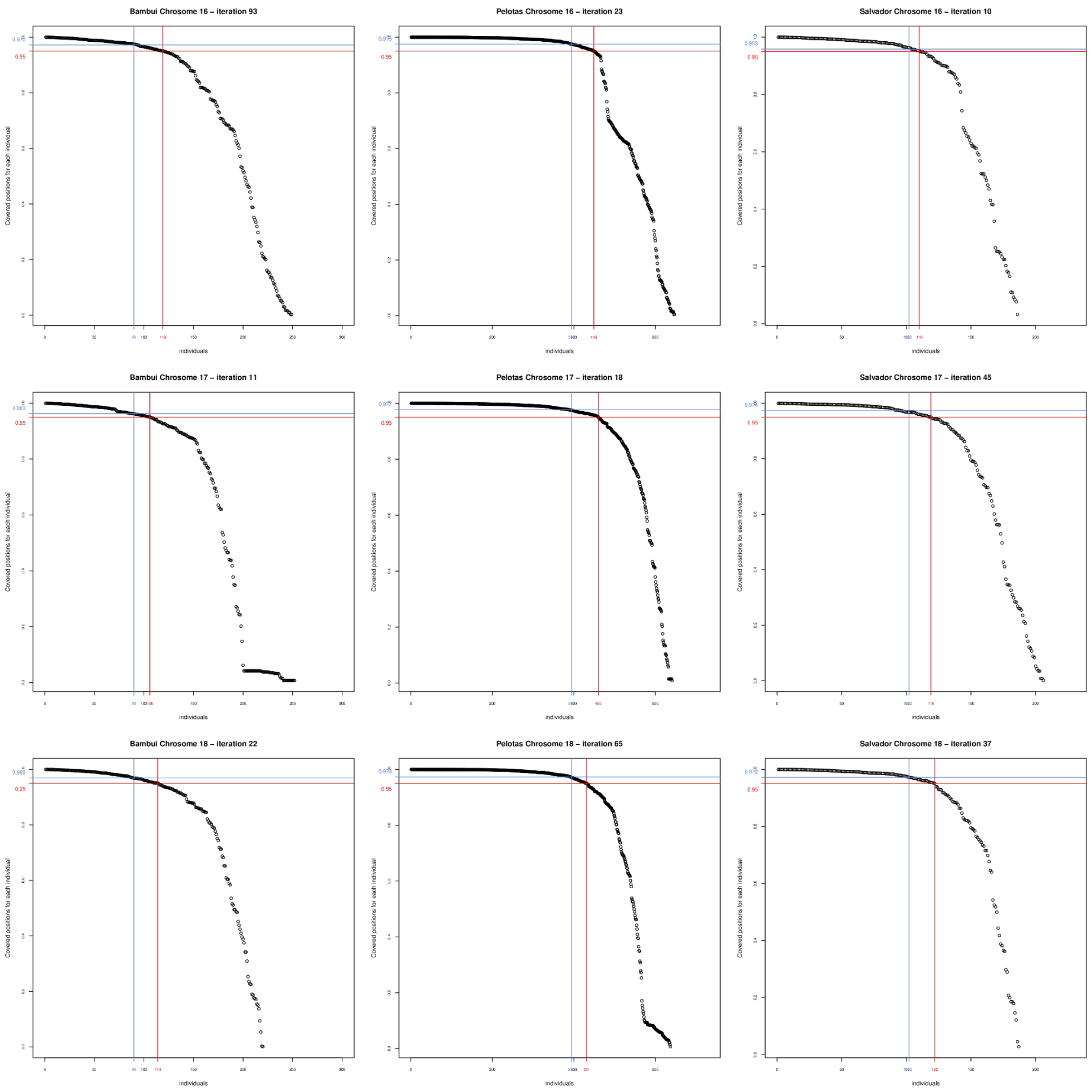

Bambui Chrosome 16 – iteration 93
Pelotas Chrosome 16 – iteration 23
Salvador Chrosome 16 – iteration 10
Bambui Chrosome 17 – iteration 11
Pelotas Chrosome 17 – iteration 18
Salvador Chrosome 17 – iteration 45
Bambui Chrosome 18 – iteration 22
Pelotas Chrosome 18 – iteration 65
Salvador Chrosome 18 – iteration 37
Covered positions for each individual
individuals
0.95

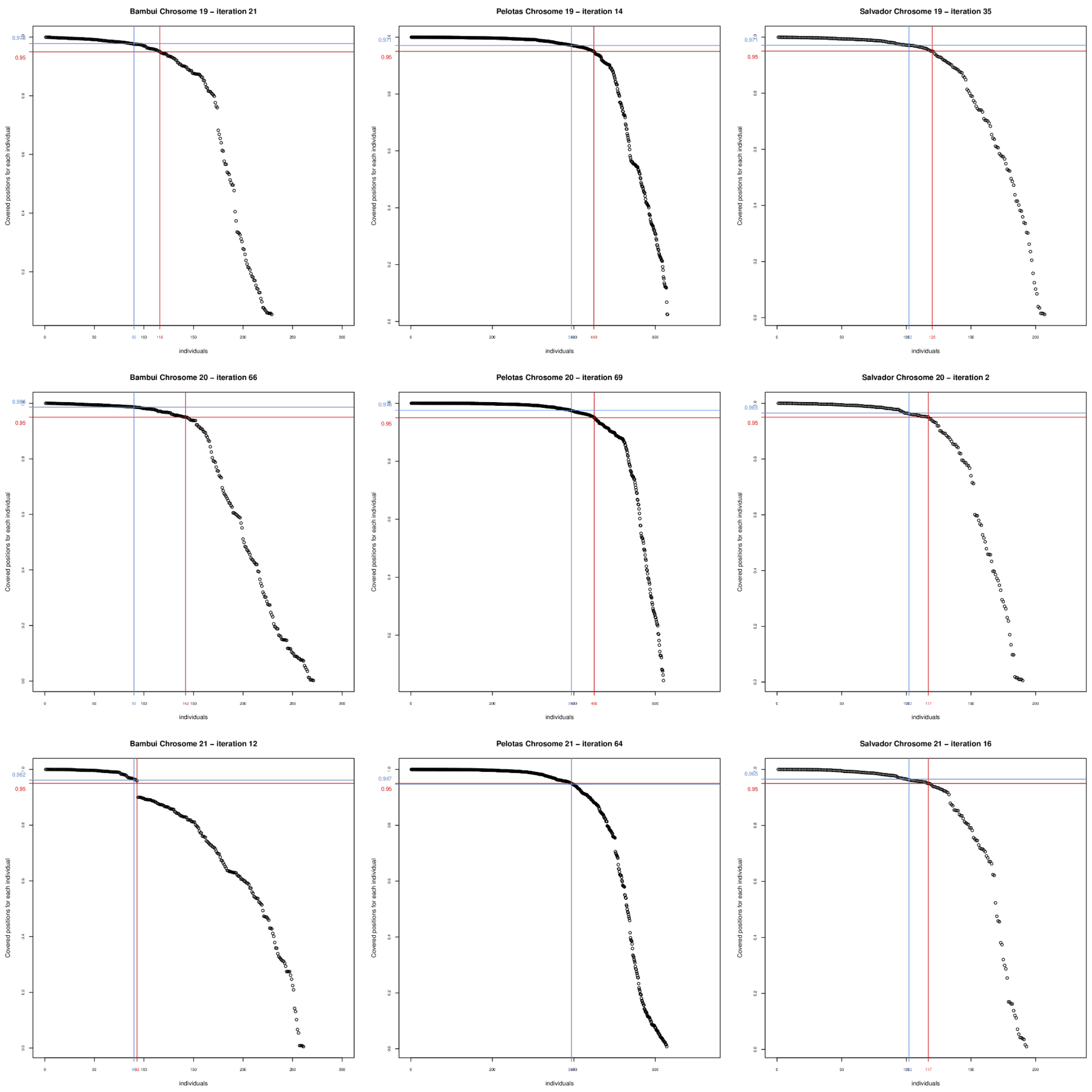
Bambui Chrosome 19 – iteration 21
Pelotas Chrosome 19 – iteration 14
Salvador Chrosome 19 – iteration 35
Bambui Chrosome 20 – iteration 66
Pelotas Chrosome 20 – iteration 69
Salvador Chrosome 20 – iteration 2
Bambui Chrosome 21 – iteration 12
Pelotas Chrosome 21 – iteration 64
Salvador Chrosome 21 – iteration 16
Covered positions for each individual
individuals
0.95

Figure S5. Each plot shows the best iteration for the reconstruction of chromosomes for each autosome and each population. Plot columns represent the three populations (Bambui, Pelotas and Salvador) and plot rows represent the 22 autosomes. The x axis of each plot shows the amount of reconstructed chromosomes, whereas the y axis shows the percentage of the base pairs of the reconstructed chromosome covered by Native American fragments. The reconstructed chromosomes are sorted from the one with the highest amount of SNPs covered by the Native American fragments, at left, to the one with the fewest, at right. The horizontal red line shows the 95% threshold and the corresponding vertical red line points the amount of reconstructed chromosomes beyond this threshold. The vertical blue line corresponds to the autosome in this population that has the fewest amount of reconstructed chromosomes beyond the 95% threshold. The goal of the analysis is to build diploid individuals with 22 pairs of chromosomes. Thus, the total number of reconstructed individuals in a population is determined by the autosome with less reconstructed chromosomes. The horizontal blue line shows the minimum percentage of covered positions in the reconstructed chromosomes after setting the number of reconstructed chromosomes.

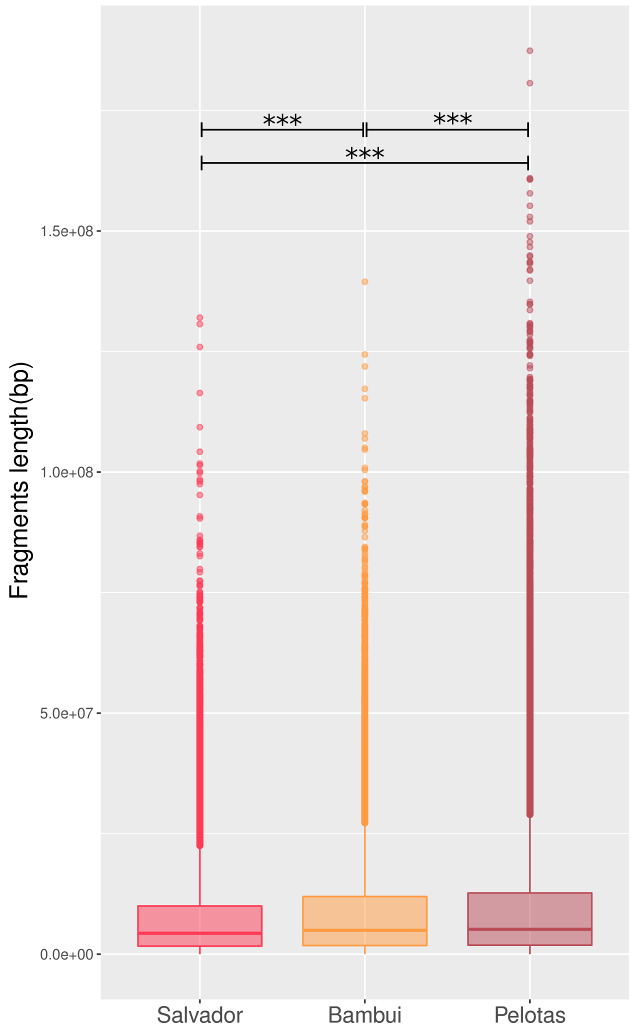

Figure S6. **Fragments lengths** per population. Comparison of the Native American ancestry fragments lengths between the three populations. Differences are significant (p-value <0.005) after Wilcoxon test and Bonferroni multiple test correction.

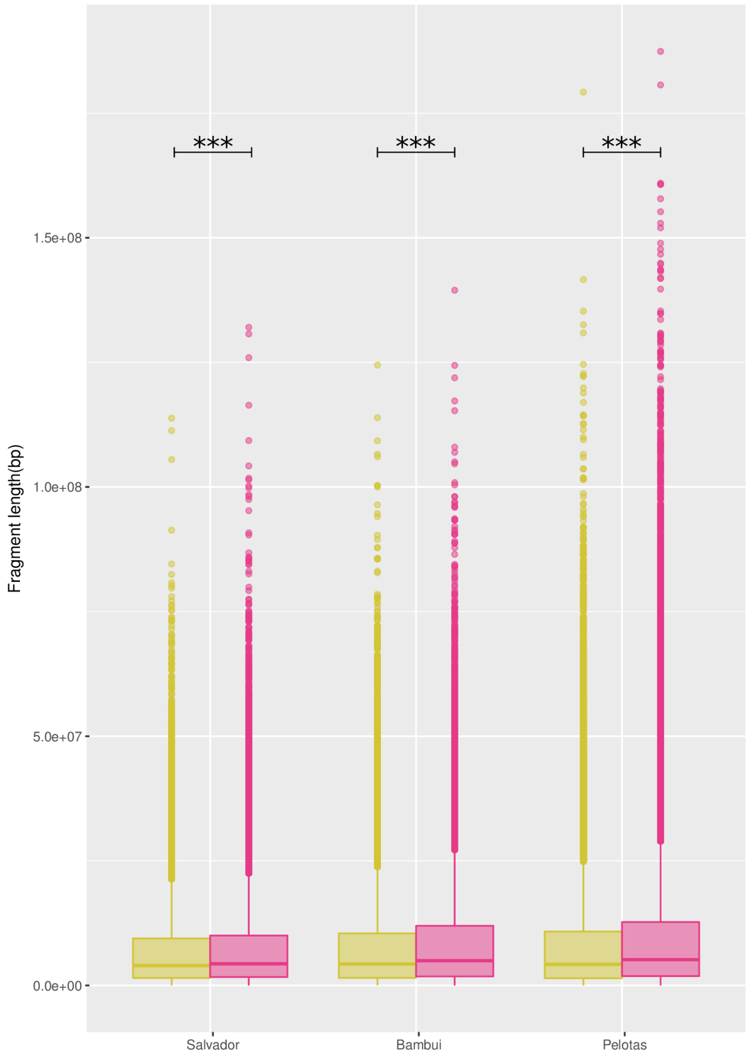

Figure S7. **Fragments lengths.** Fragments included in the final reconstruction (in pink) set tend to be larger than fragments that remain in the reconstructed chromosomes that are discarded since do not pass the reconstruction gaps missing positions thresholds (in yellow).

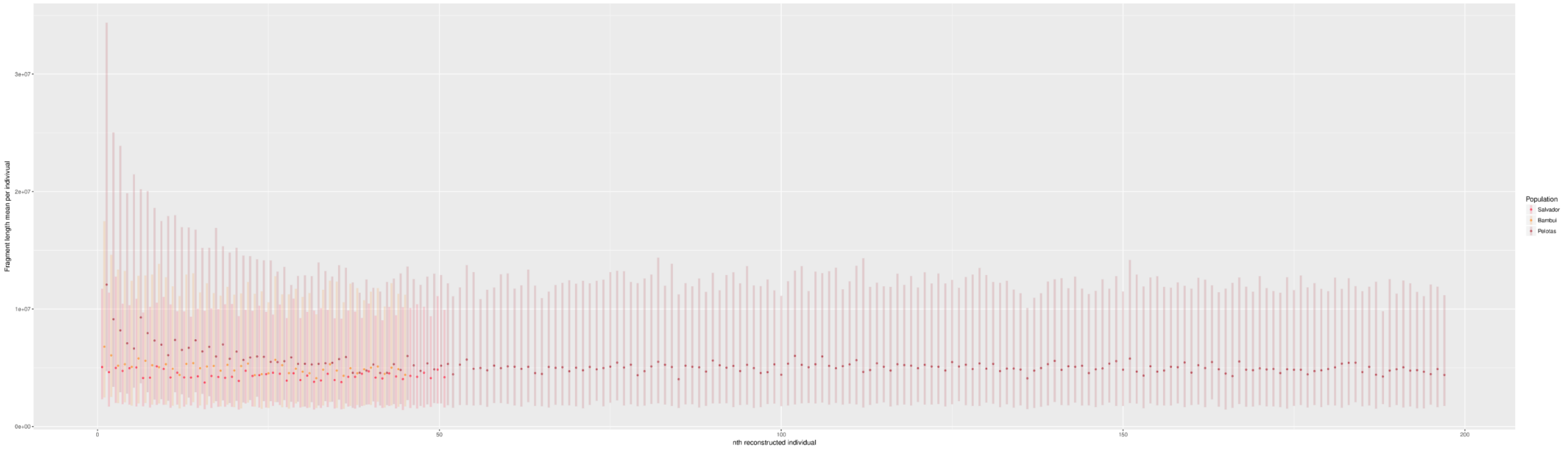

Figure S8. **Fragments lengths.** Distribution of fragment lengths per reconstructed individual, on the y axis. The individuals are sorted from higher to lower amount of genome base pairs covered by the fragments, on the x axis. The first reconstructed individuals tend to have longer fragments and larger length variability compared to the last reconstructed individual.

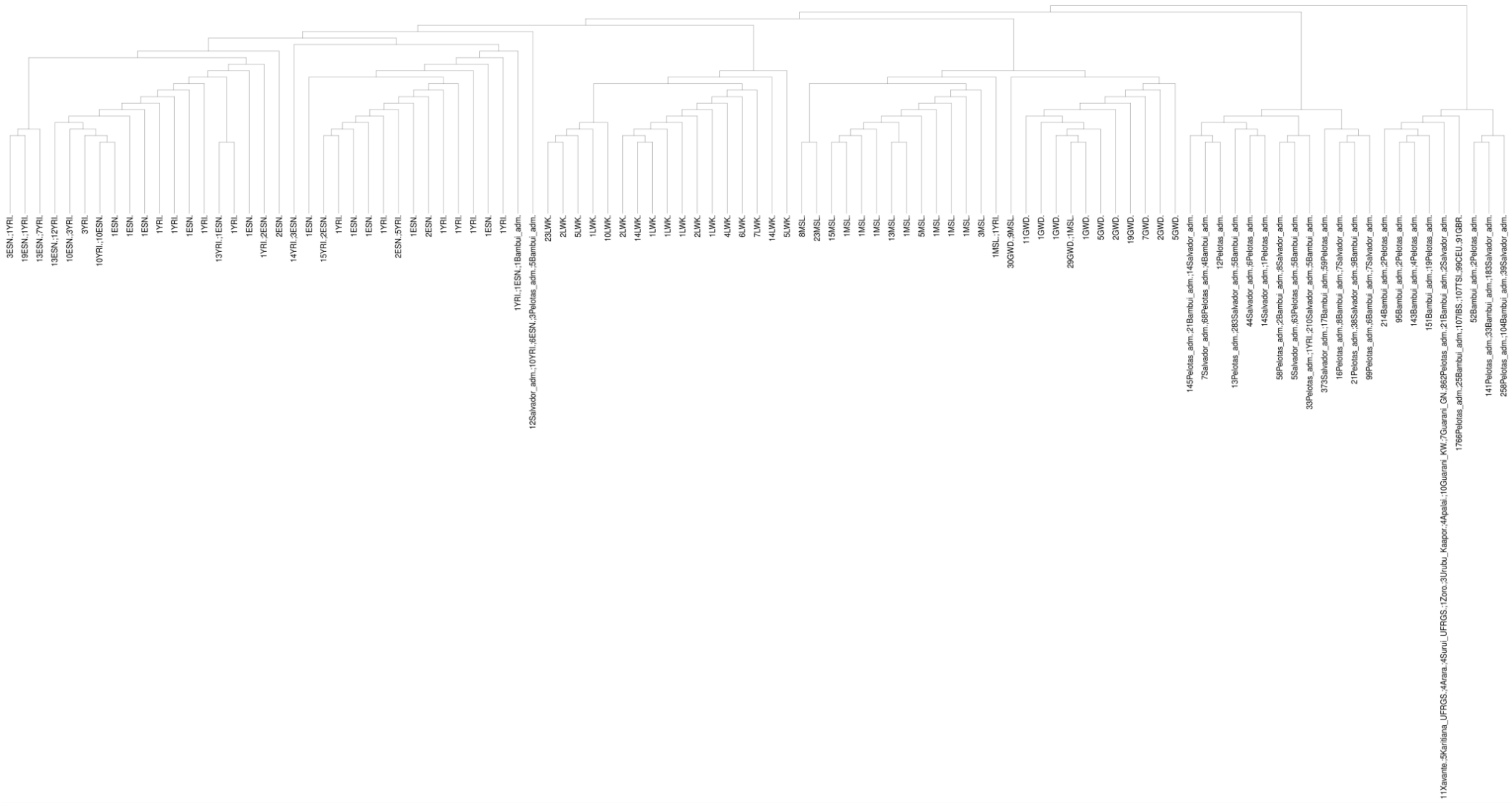

Figure S9. **Finestructure tree of Dataset A. Seed 1.** The branches are defined at height 11 of the dendrogram

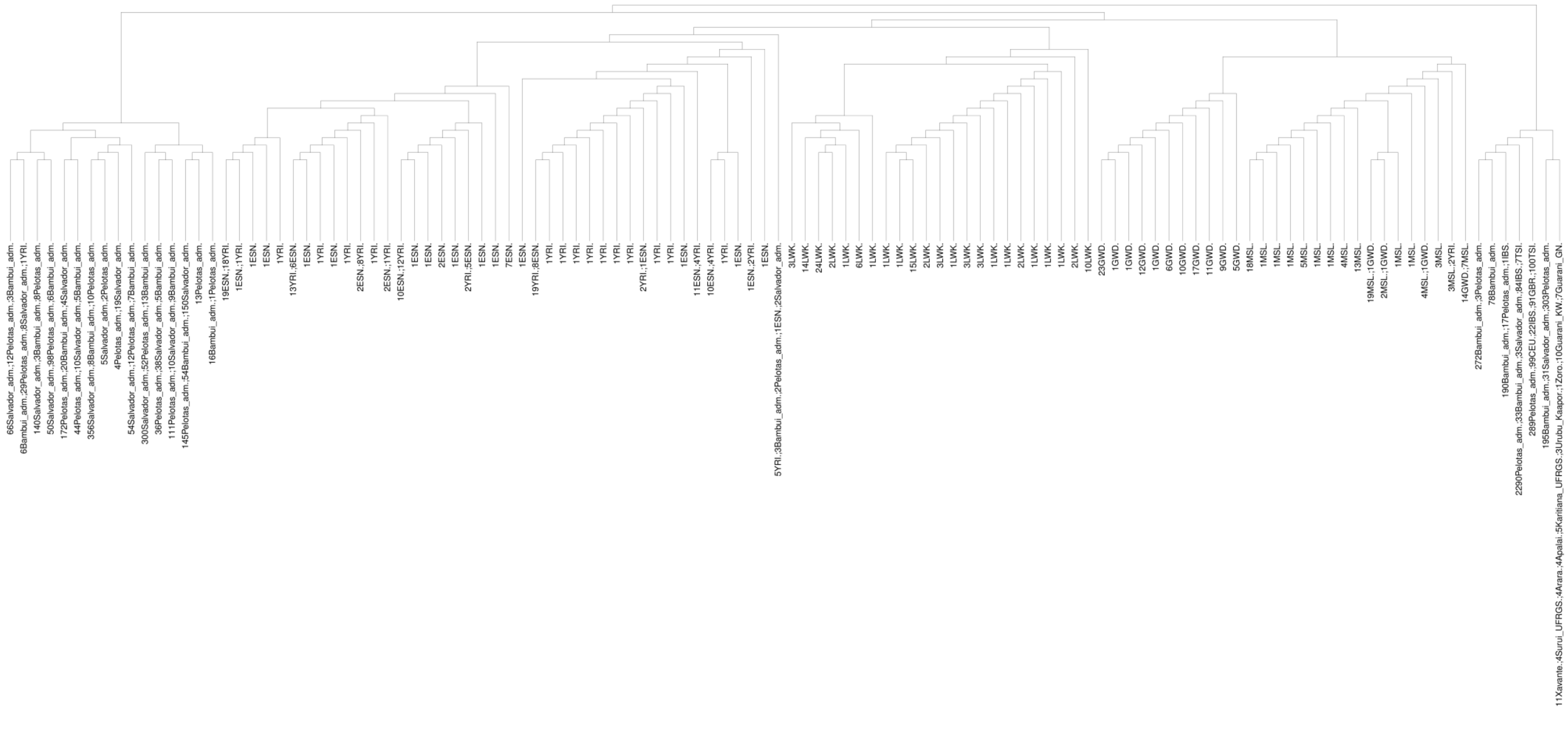

Figure S10. Finestructure tree of Dataset A. Seed 100. The branches are defined at height 11 of the dendrogram

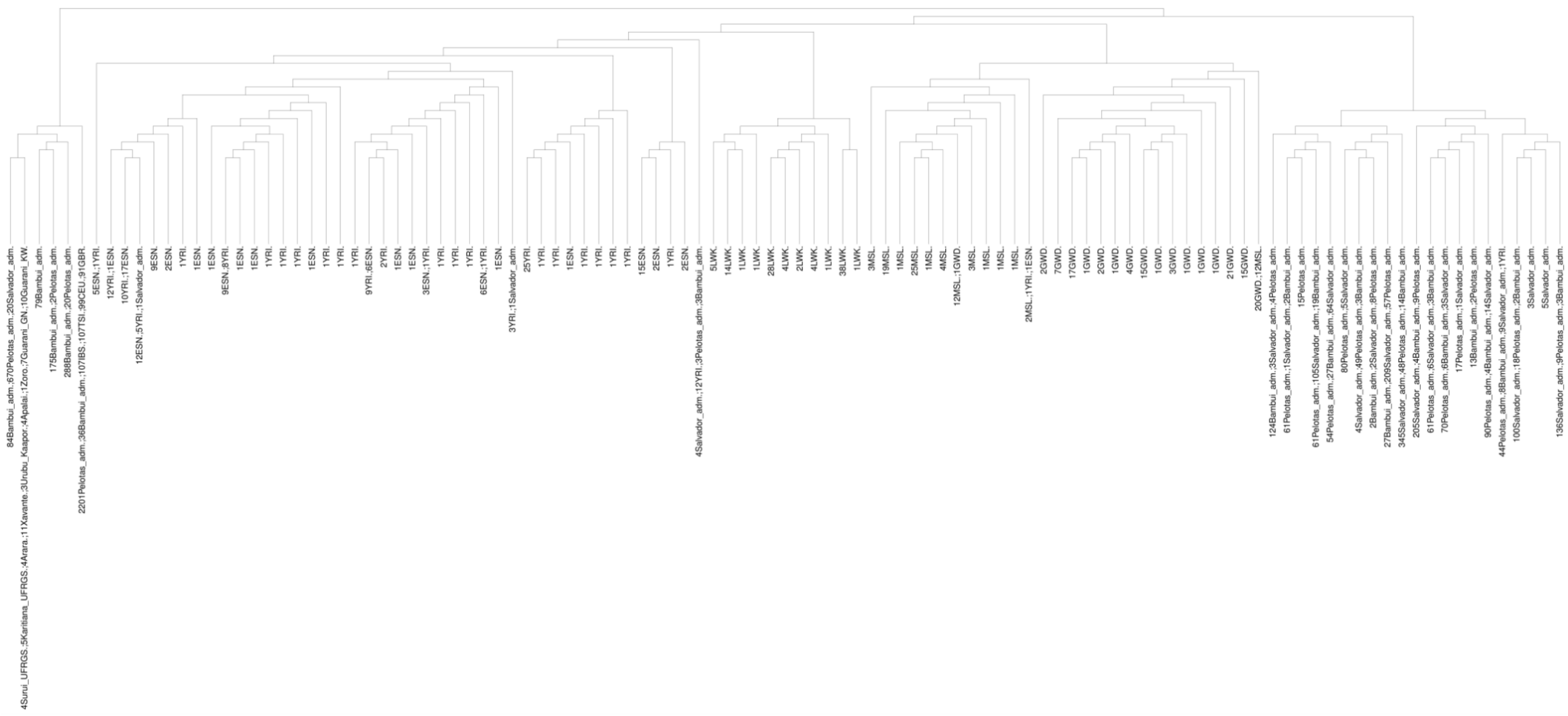

Figure S11. Finestructure tree of Dataset A. Seed 300. The branches are defined at height 11 of the dendrogram

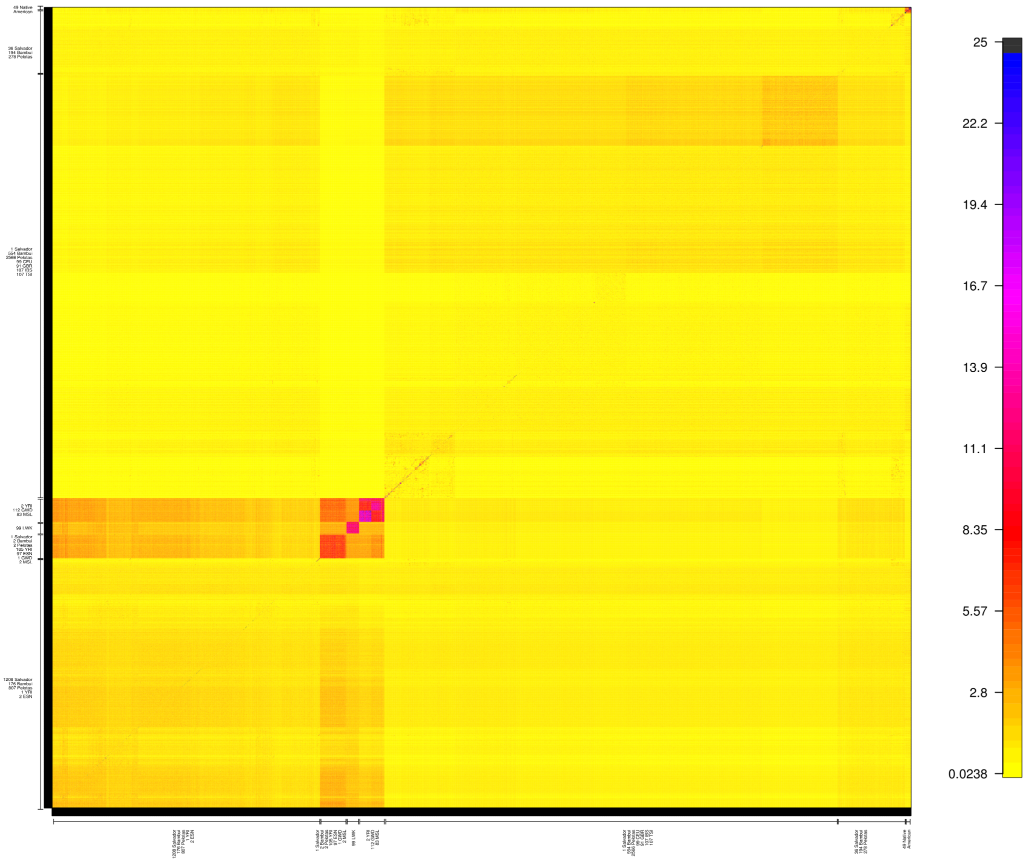

Figure S12. **Chromopainter matrix of Dataset A.** Seed 100. Total chunklengths given by donor populations, as rows, to the recipient populations, as columns.

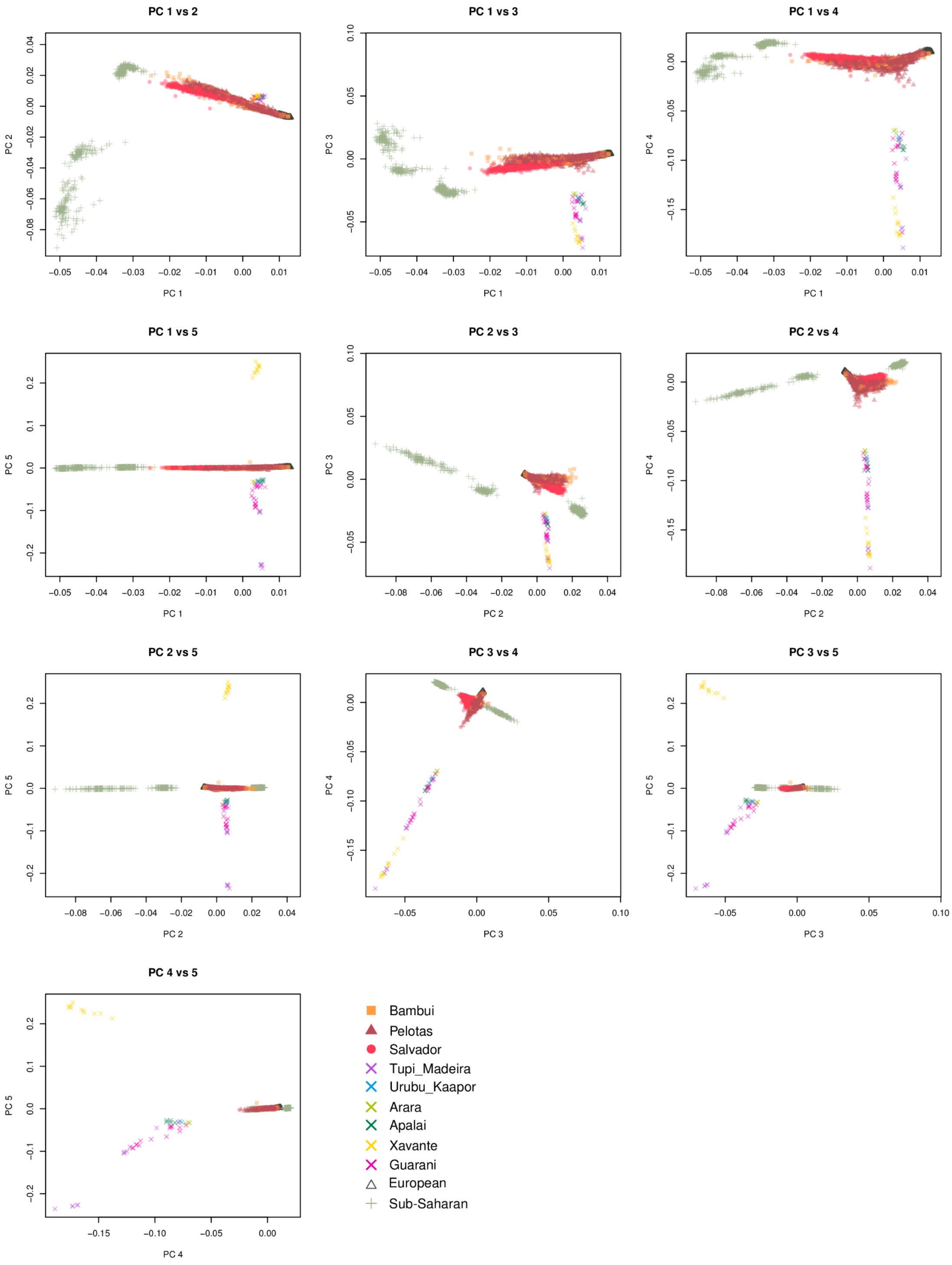

Figure S13. **Principal Component Analysis** based on the Chromopainter chunkcounts coancestry matrix of Dataset A of seed 100

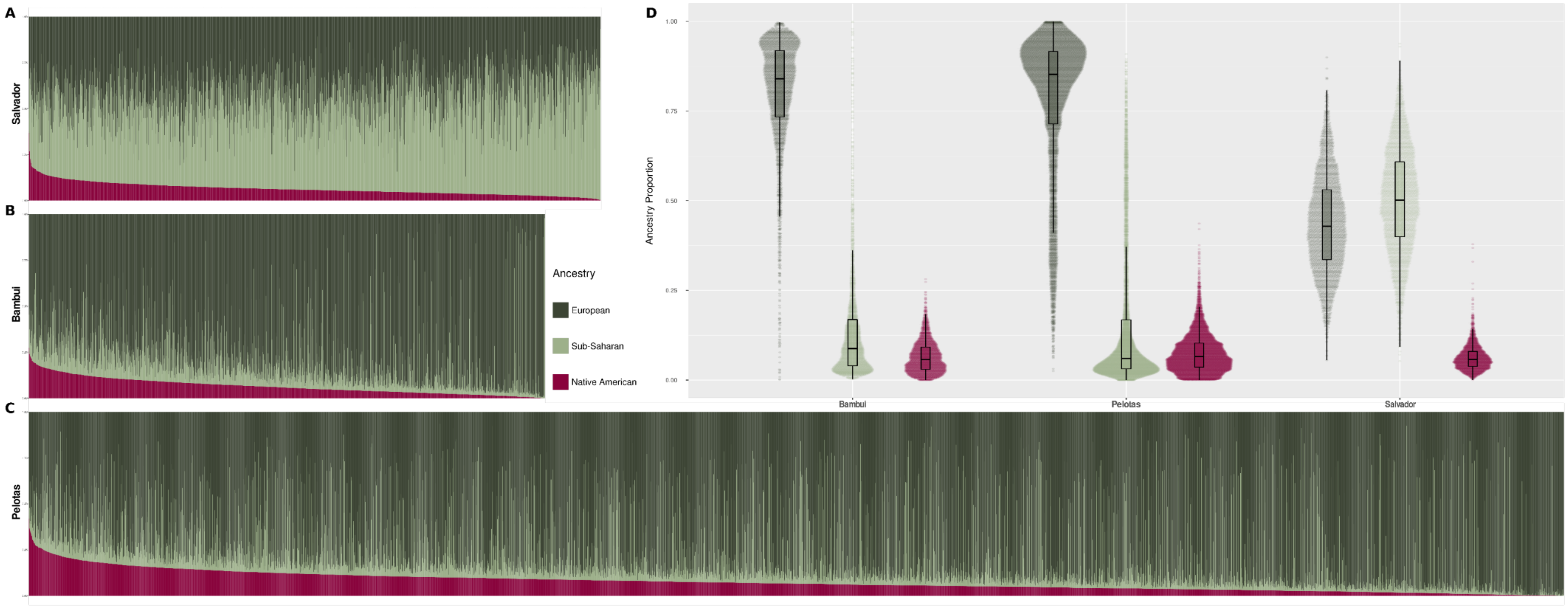

Figure S14. **Local Ancestry.** Local ancestry inference for each individual in Salvador, Bambui and Pelotas (A,B and C) sorted by each sample's proportion of Native American ancestry . The population distribution of each ancestry is shown in D.

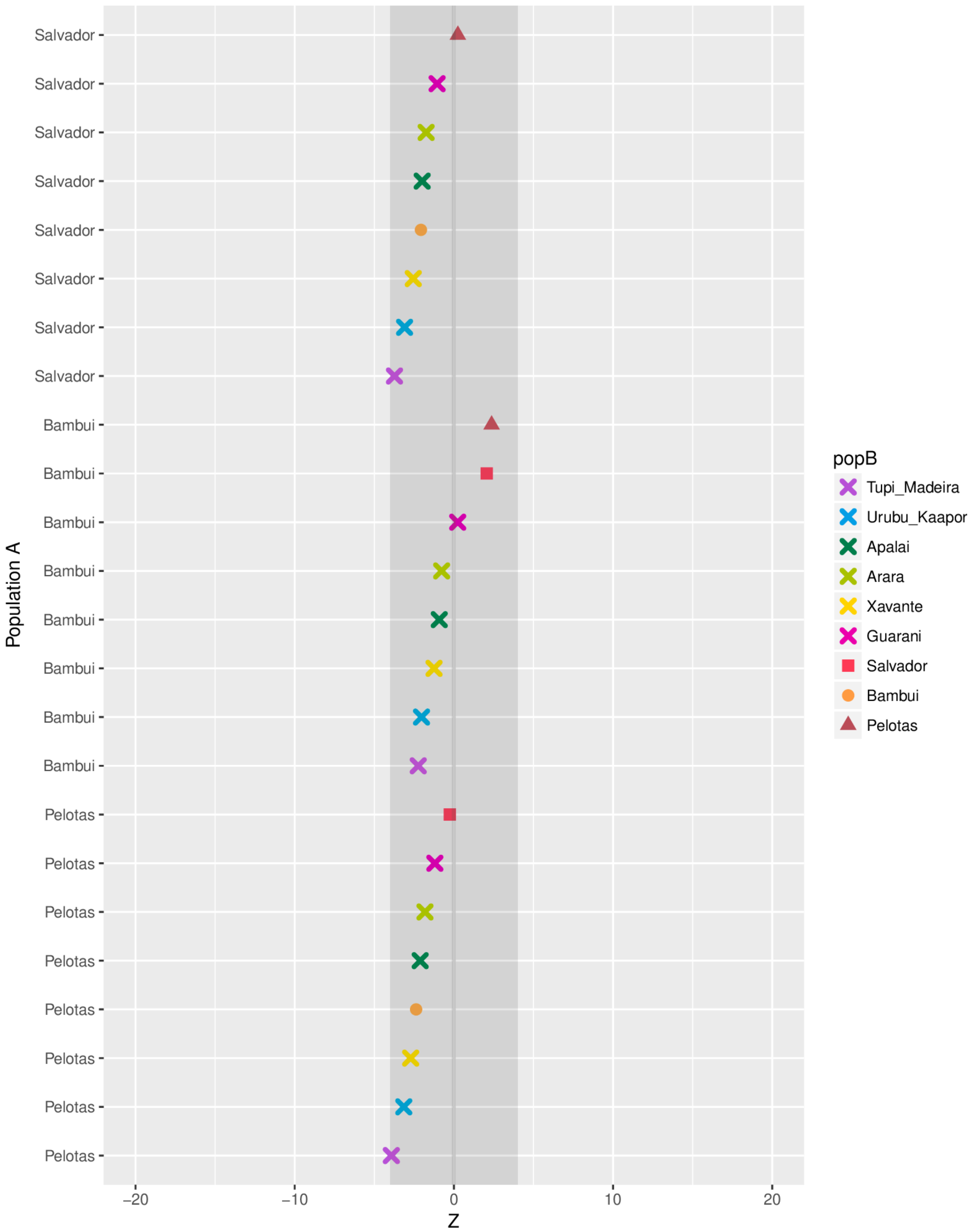

Figure S15. f4 for European and sub-Saharan admixture control. Z-score for f4(Population A, PopulationB; IBS, YRI). The darker area includes the values not significantly different from 0 (-4,4).

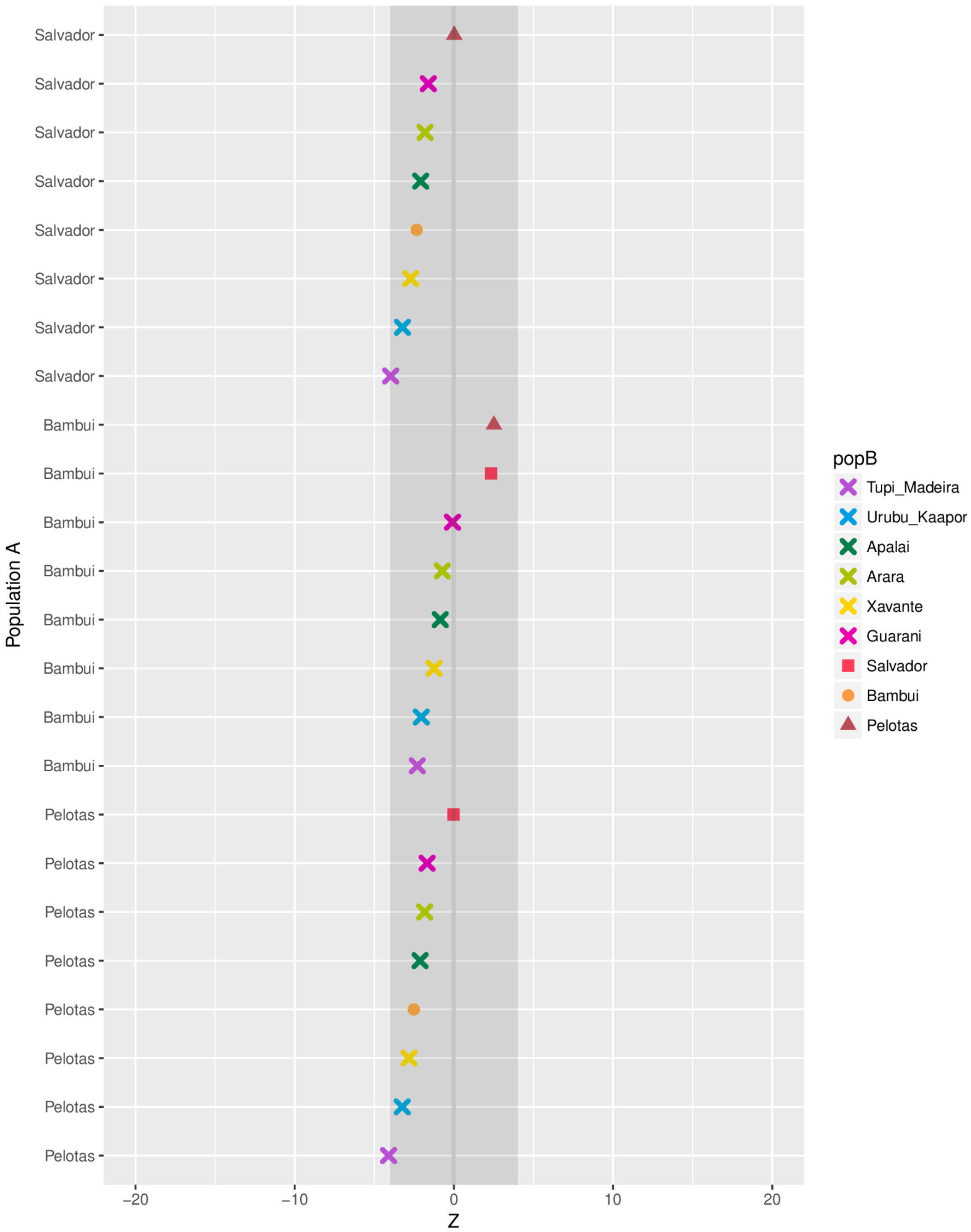

Figure S16. **f4 for European and sub-Saharan admixture control.** Z-score for f4(Population A, PopulationB; IBS, LWK). The dark area includes the values not significantly different from 0 (-4,4).

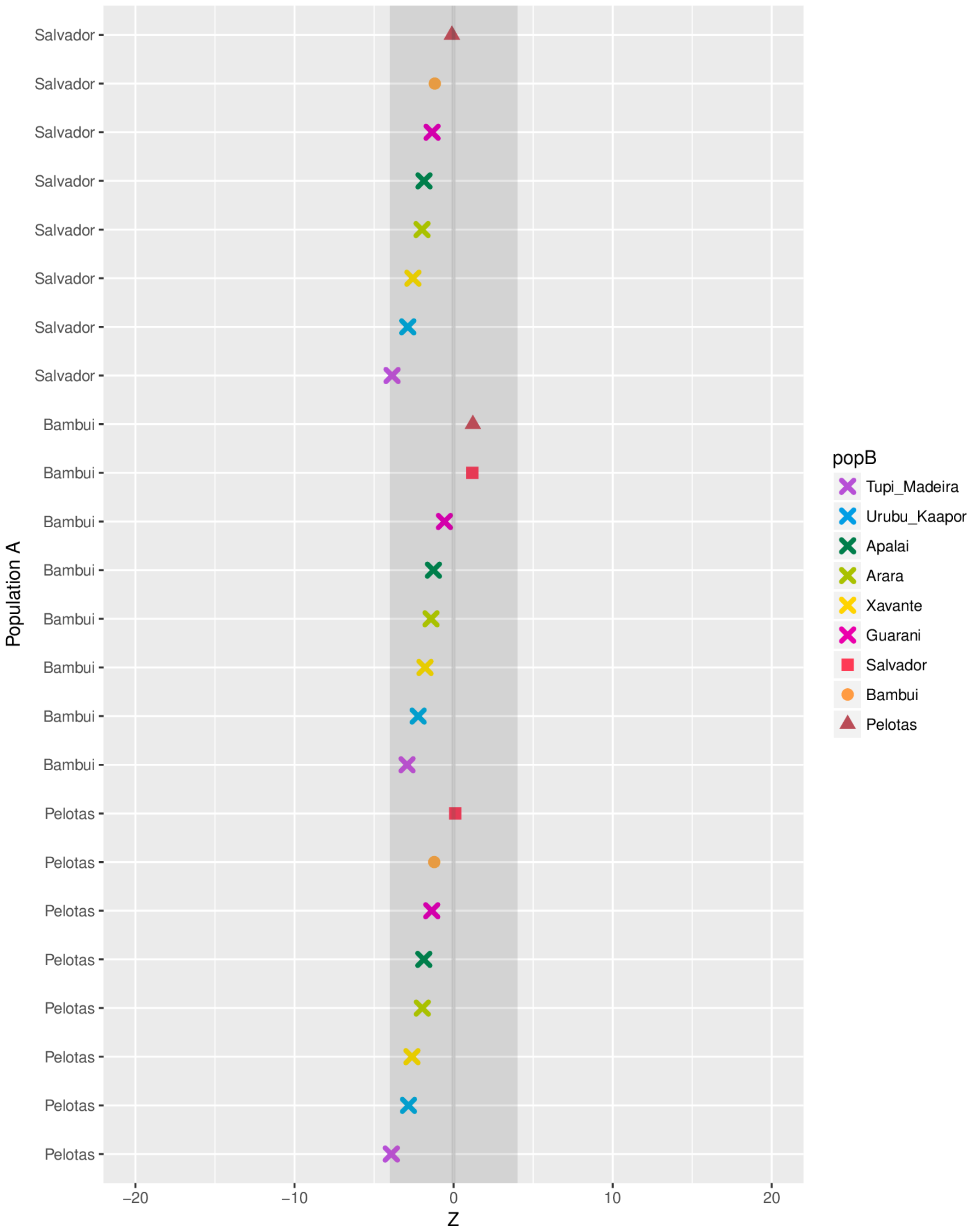

Figure S17. f4 for European and sub-Saharan admixture control. Z-score for f4(Population A, PopulationB; CEU, YRI). The dark area includes the values not significantly different from 0 (-4,4).

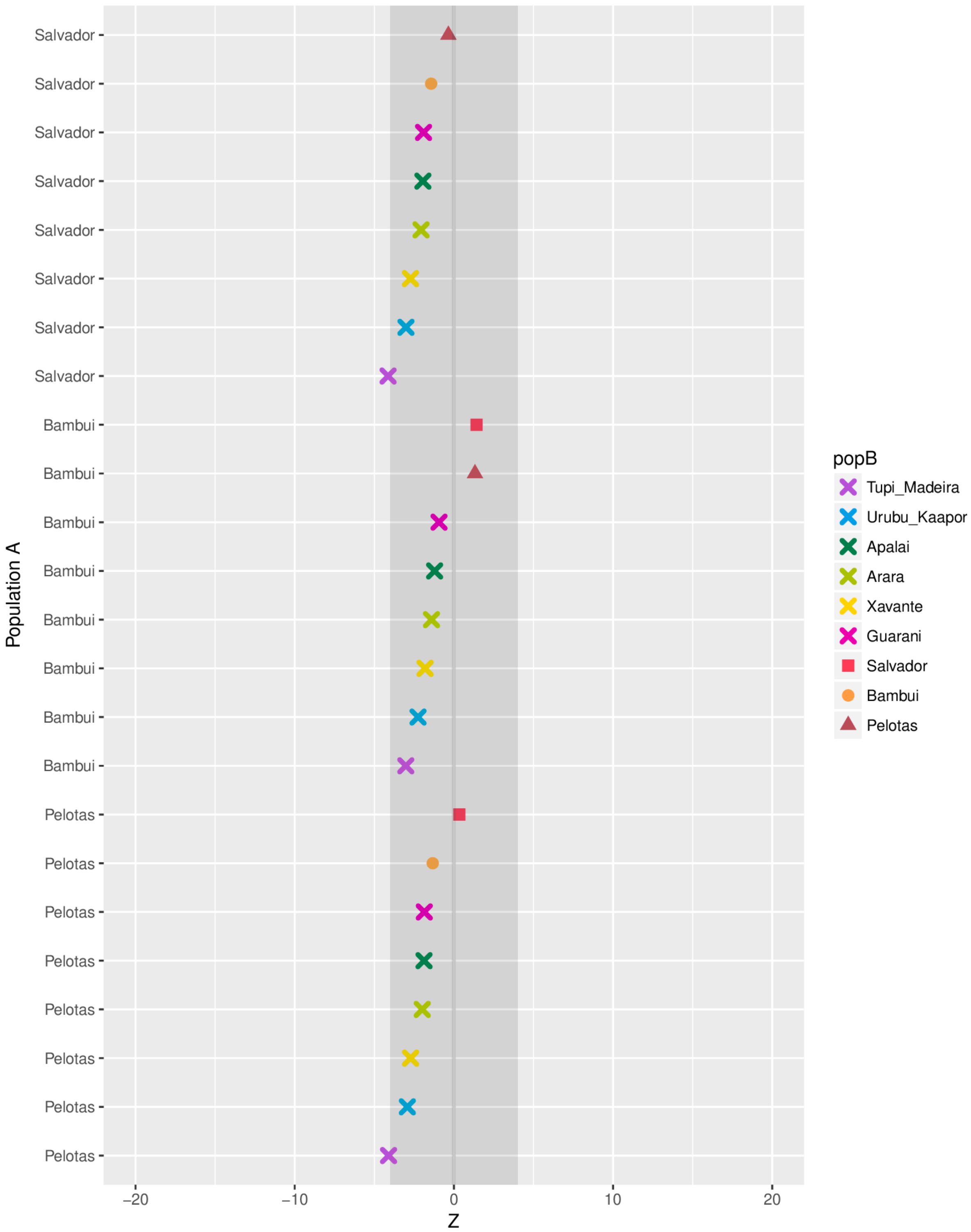

Figure S18. **f4 for European and sub-Saharan admixture control.** Z-score for f4(Population A, PopulationB; CEU, YRI). The dark area includes the values not significantly different from 0 (-4,4).

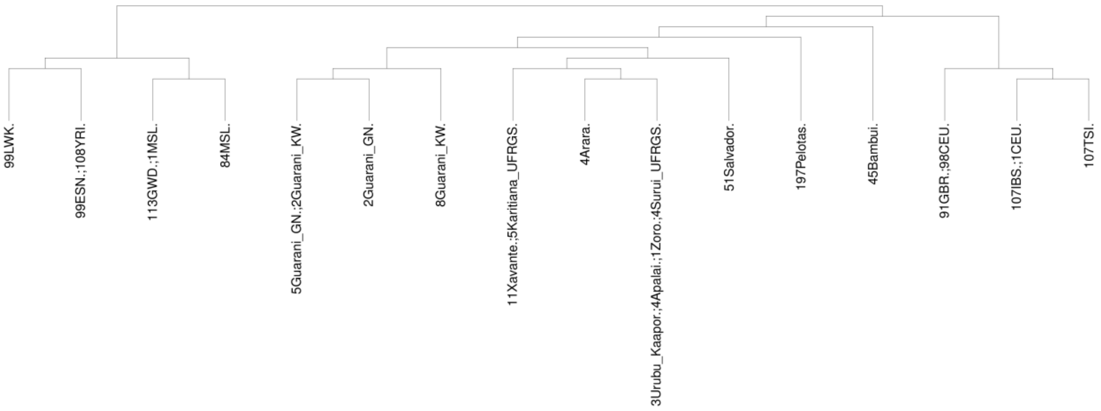

Figure S19. **Finestructure tree of Dataset B.** Seed 1. The branches are defined at height 4 of the dendrogram. Salvador, Bambui and Pelotas populations are made of the reconstructed Native American individuals

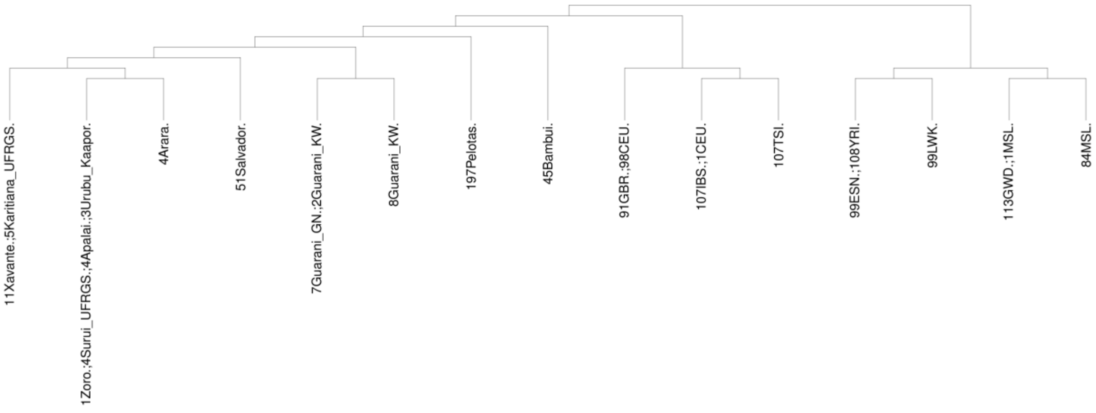

Figure S20. **Finestructure tree of Dataset B.** Seed 100. The branches are defined at height 4 of the dendrogram. Salvador, Bambui and Pelotas populations are made of the reconstructed Native American individuals.

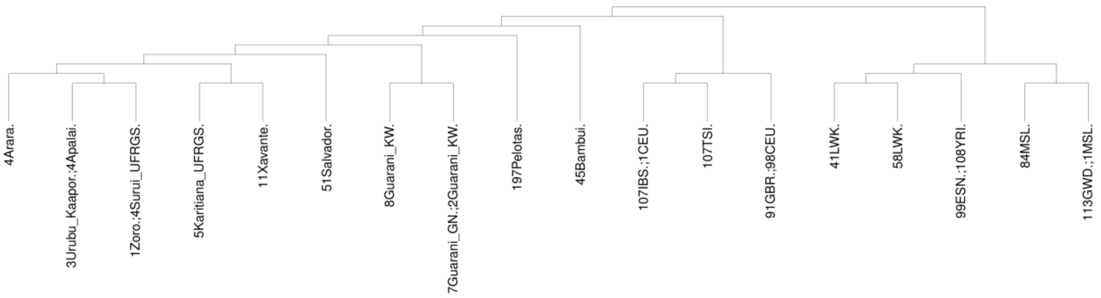

Figure S21. **Finestructure tree of Dataset B.** Seed 300. The branches are defined at height 4 of the dendrogram. Salvador, Bambui and Pelotas populations are made of the reconstructed Native American individuals

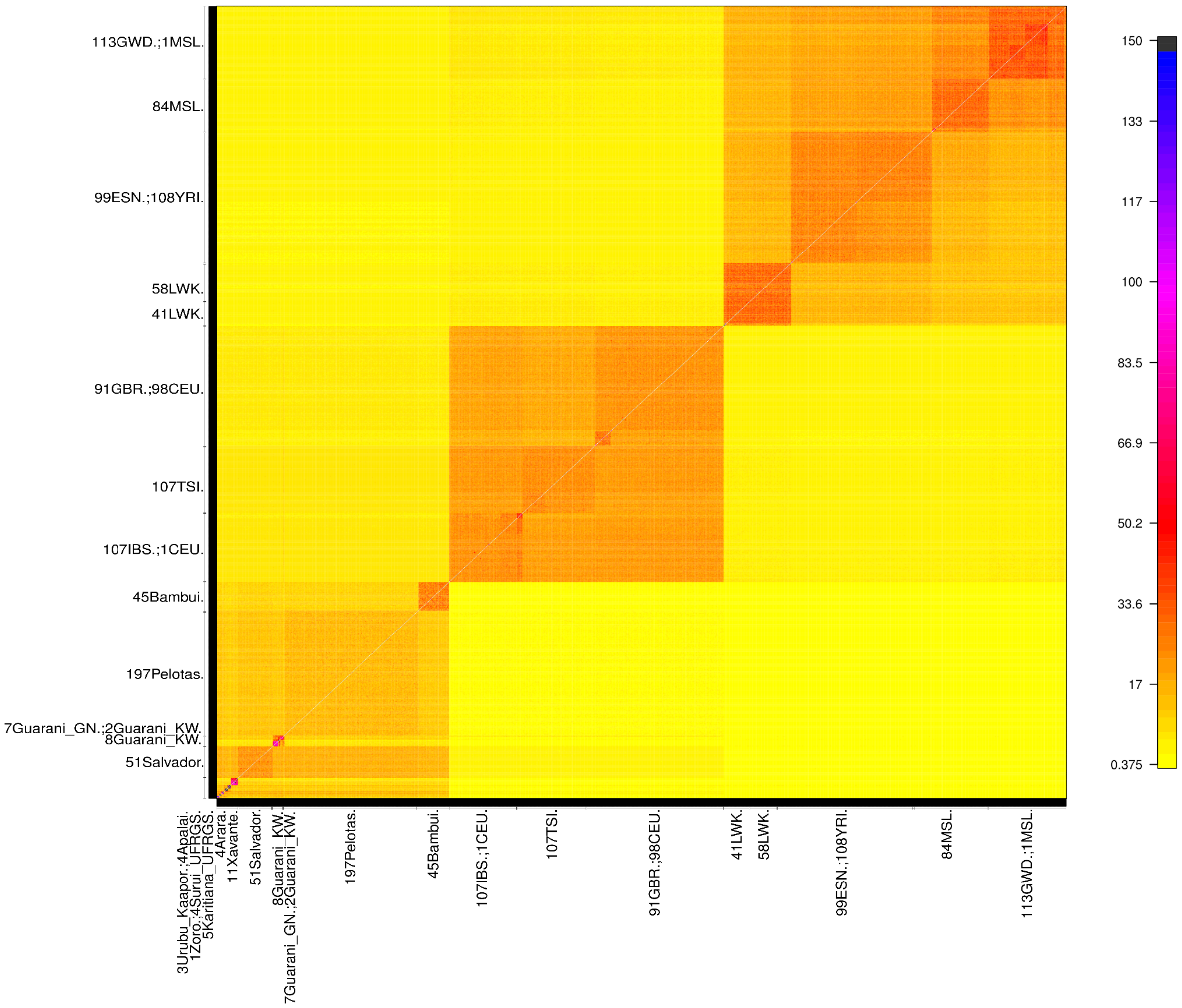

Figure S22. **Chromopainter matrix of Dataset B.** Seed 300. Total chunklengths given by donor populations, as rows, to the recipient populations, as columns.

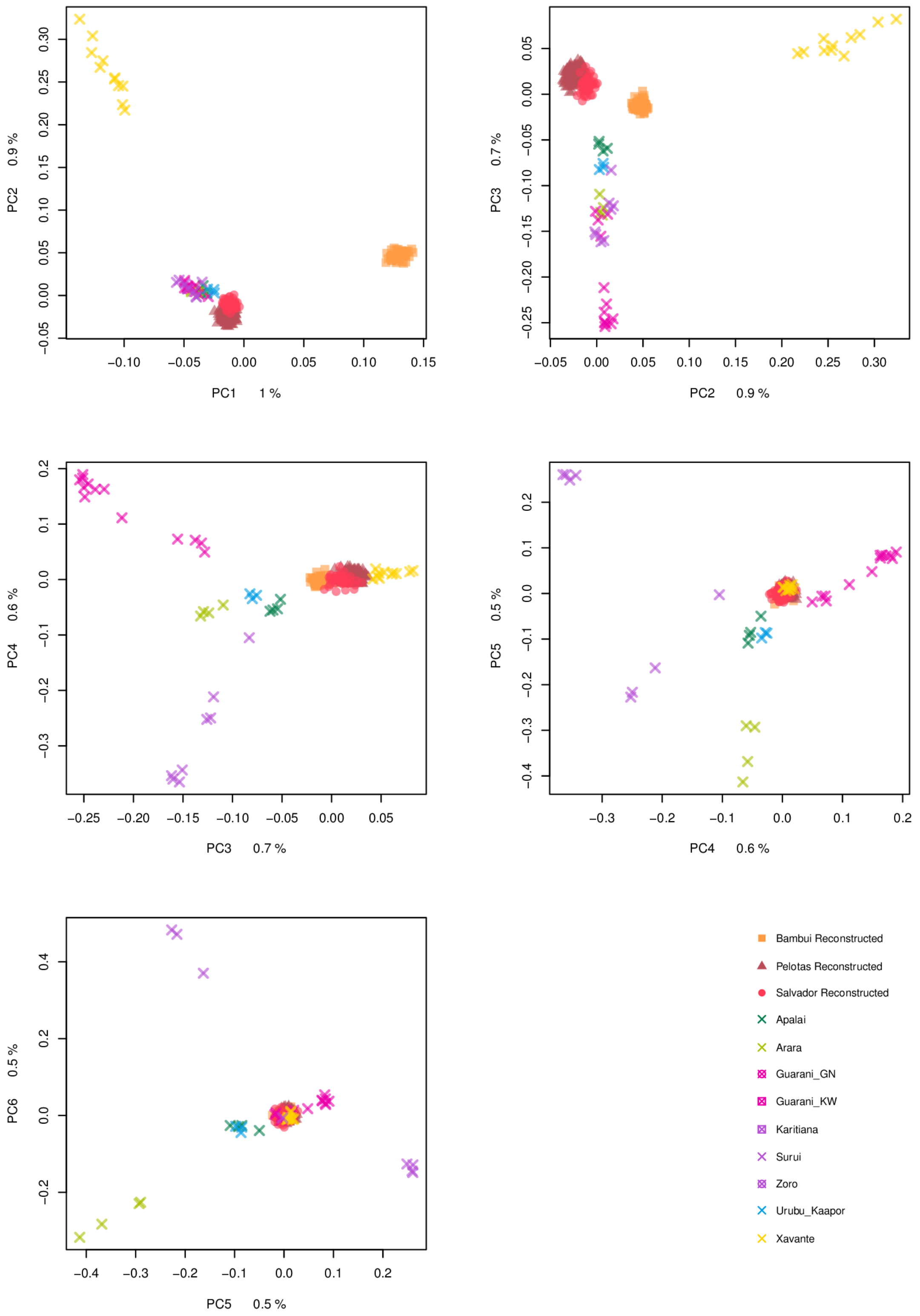

Figure S23. **Principal Component Analysis** based on the Chromopainter chunkcounts coancestry matrix of Dataset B of seed 100.

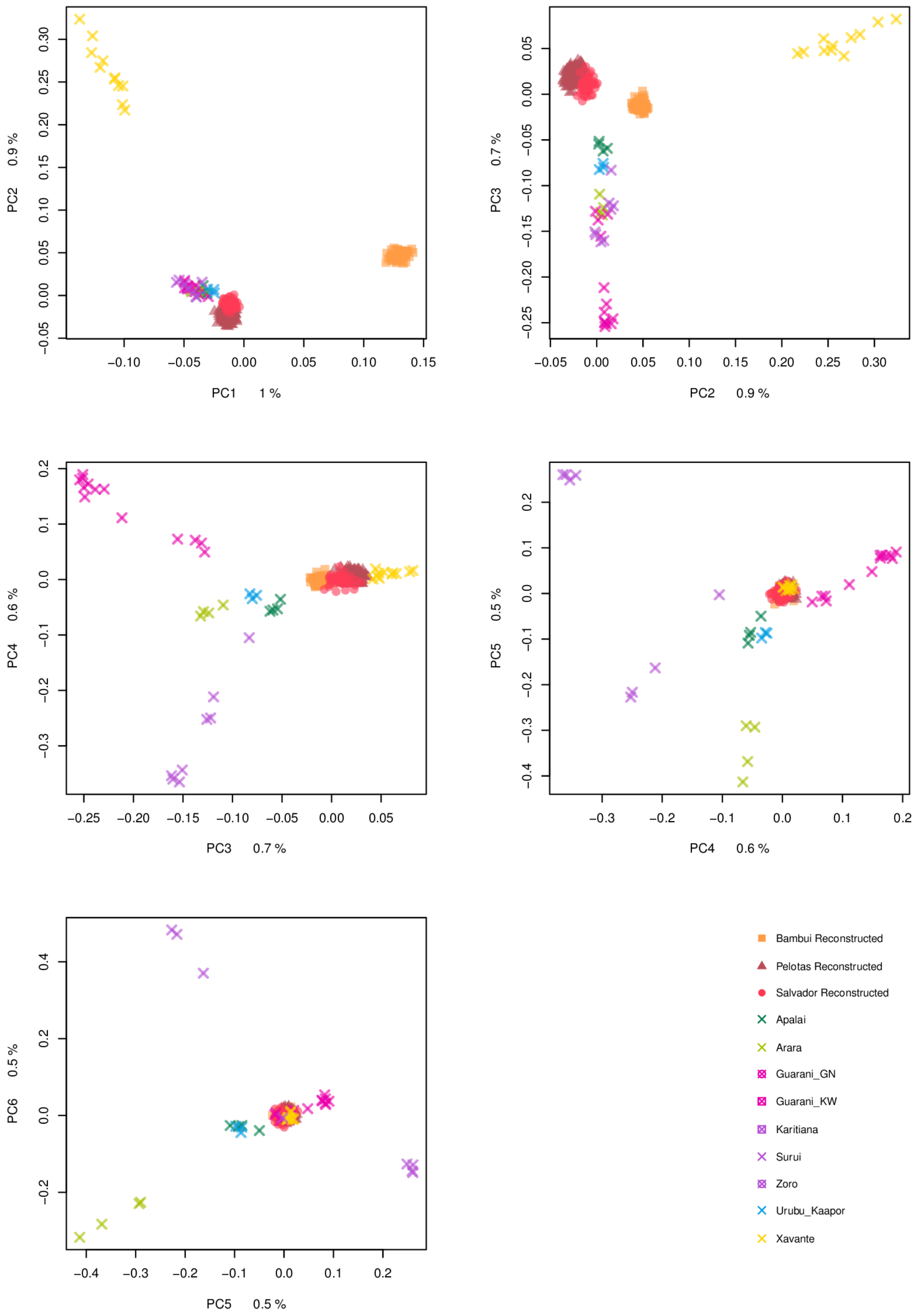

Figure S24. Principal Component Analysis of Dataset B. Principal Components 1 to 6. Colors are according to FineStructure clusters in Figure 2.

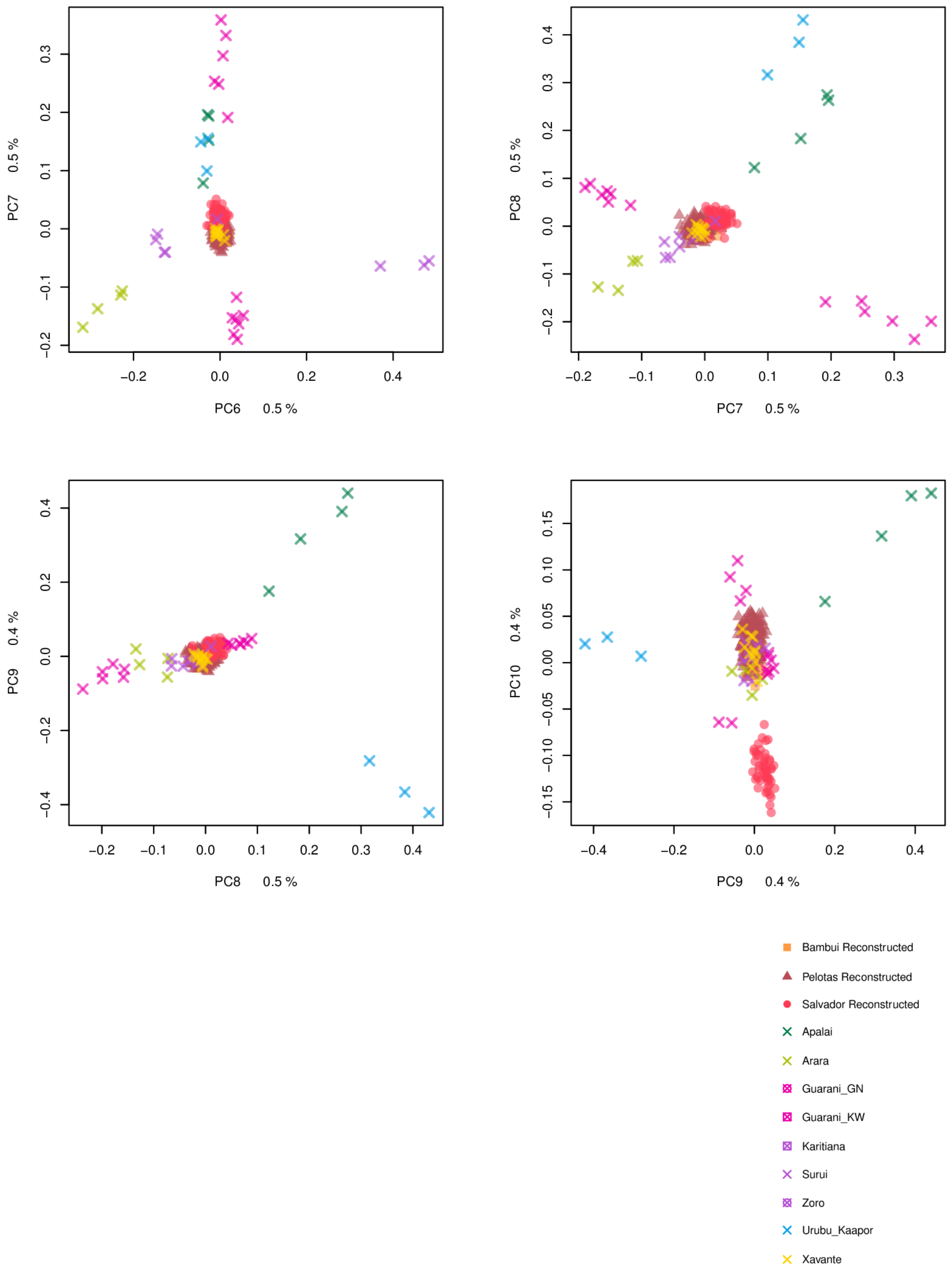

Figure S25. **Principal Component Analysis of Dataset B.** Principal Components 6 to 10. Colors are according to FineStructure clusters in Figure 2.

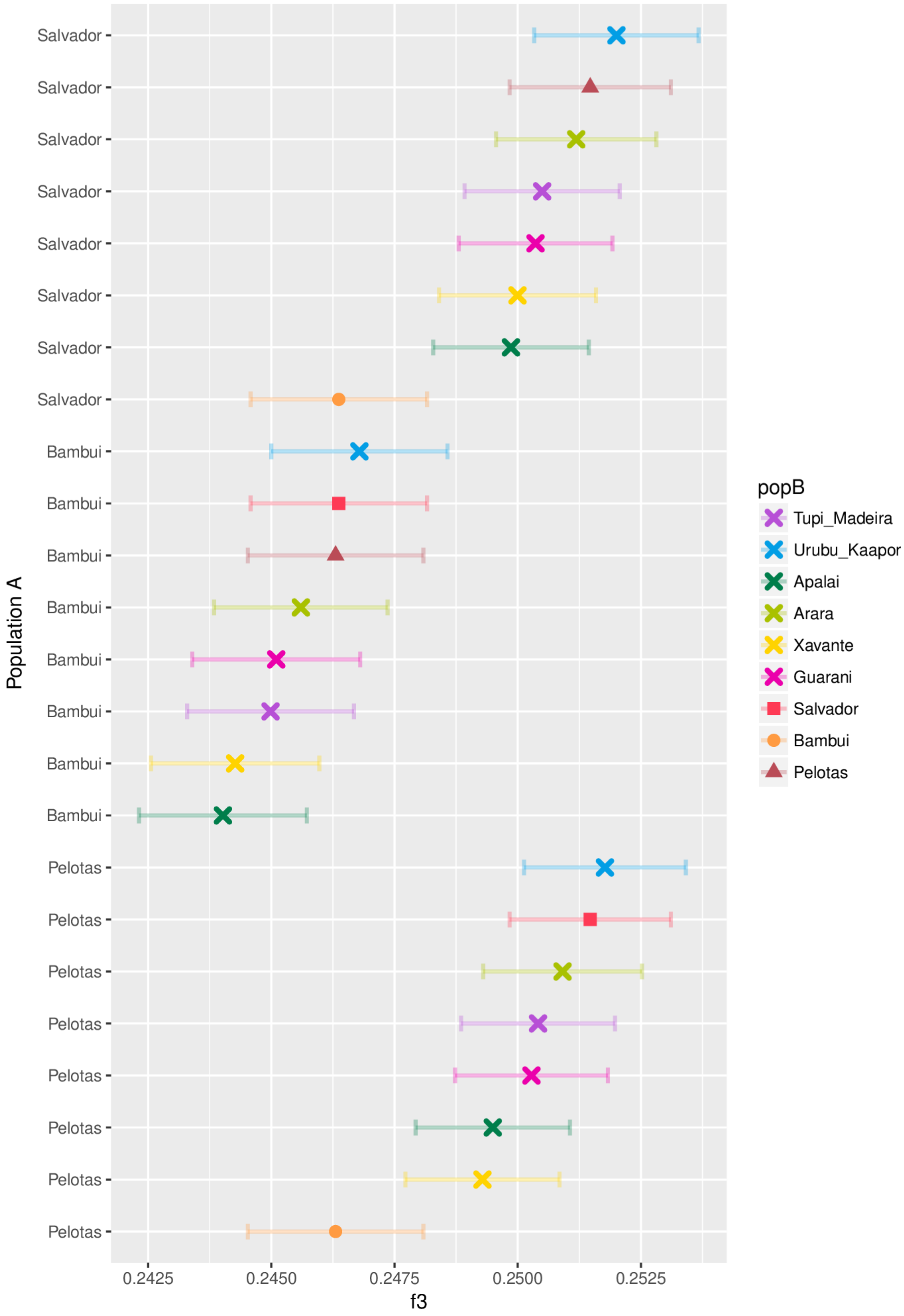

Figure S26. **f3 Outgroup analysis** in the form of f(population A, population B; YRI). Population B are the clusters defined in the FineStructure of Figure 2. Bars represent the standard error.

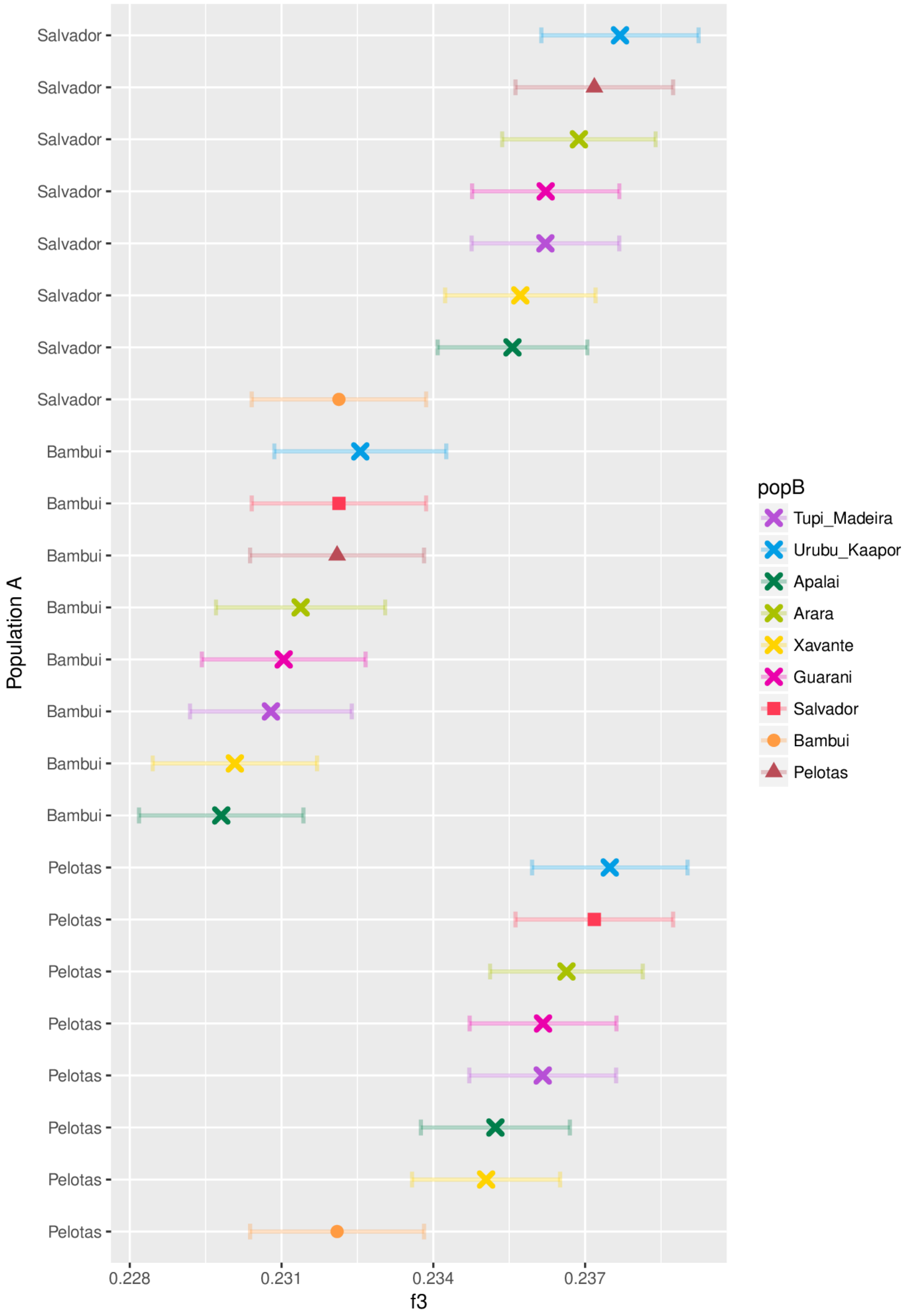

Figure S27. **f3 Outgroup analysis** in the form of f(population A, population B; LWK). Population B are the clusters defined in the FineStructure of Figure 2. Bars represent the standard error.

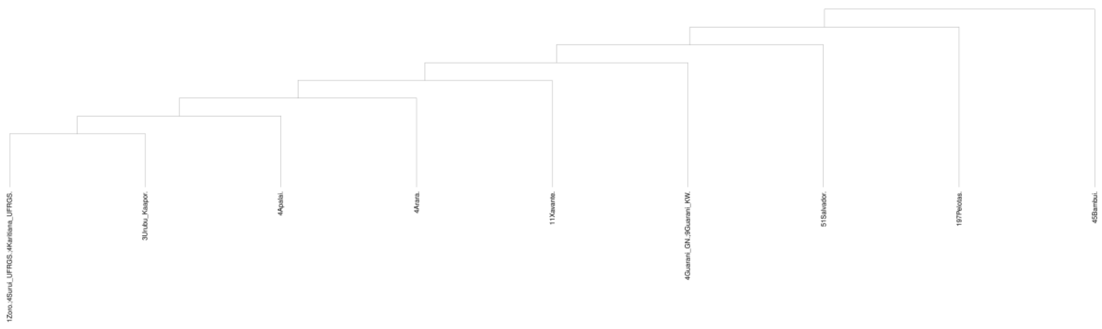

Figure S28. **Finestructure tree of Native Americans, including reconstructed populations** of Dataset B. Seed 1. The branches are defined at height 3 of the dendrogram.

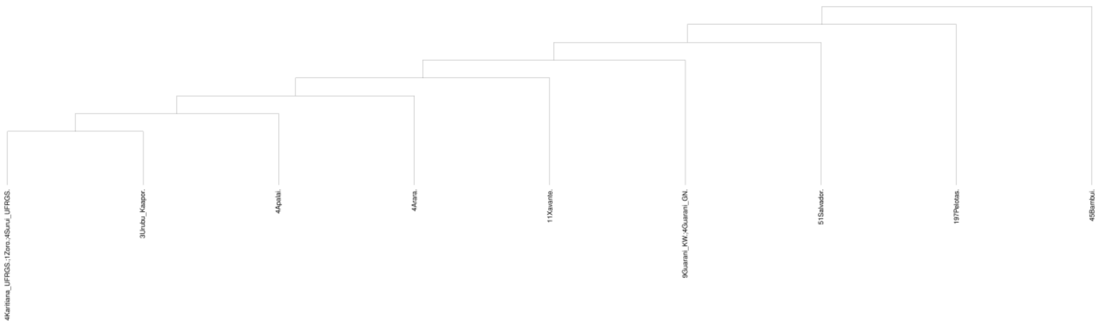

Figure S29. **Finestructure tree of Native Americans, including reconstructed populations** of Dataset B. Seed 100. The branches are defined at height 3 of the dendrogram.

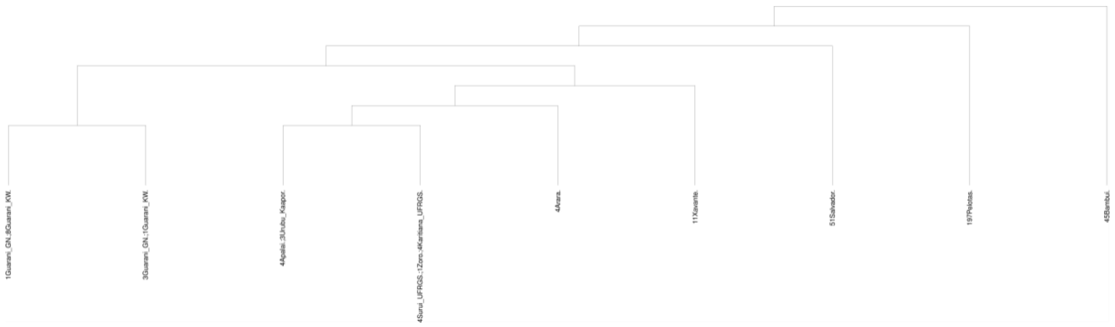

Figure S30. **Finestructure tree of Native Americans, including reconstructed populations** of Dataset B. Seed 300. The branches are defined at height 3 of the dendrogram.

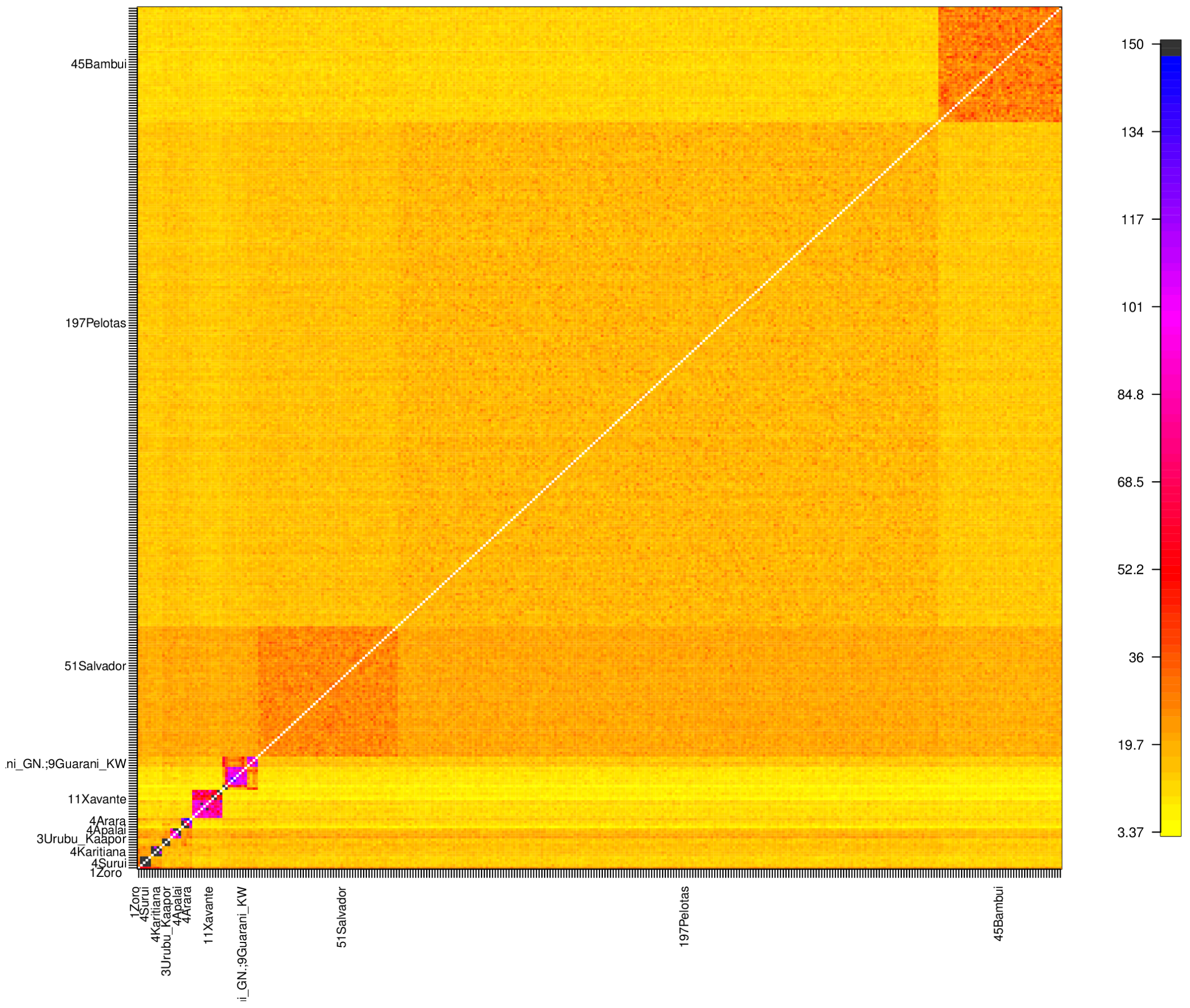

Figure S31. Chromopainter matrix of Native Americans, including reconstructed populations of Dataset B. Seed 1. Total chunklengths given by donor populations, as rows, to the recipient populations, as columns.

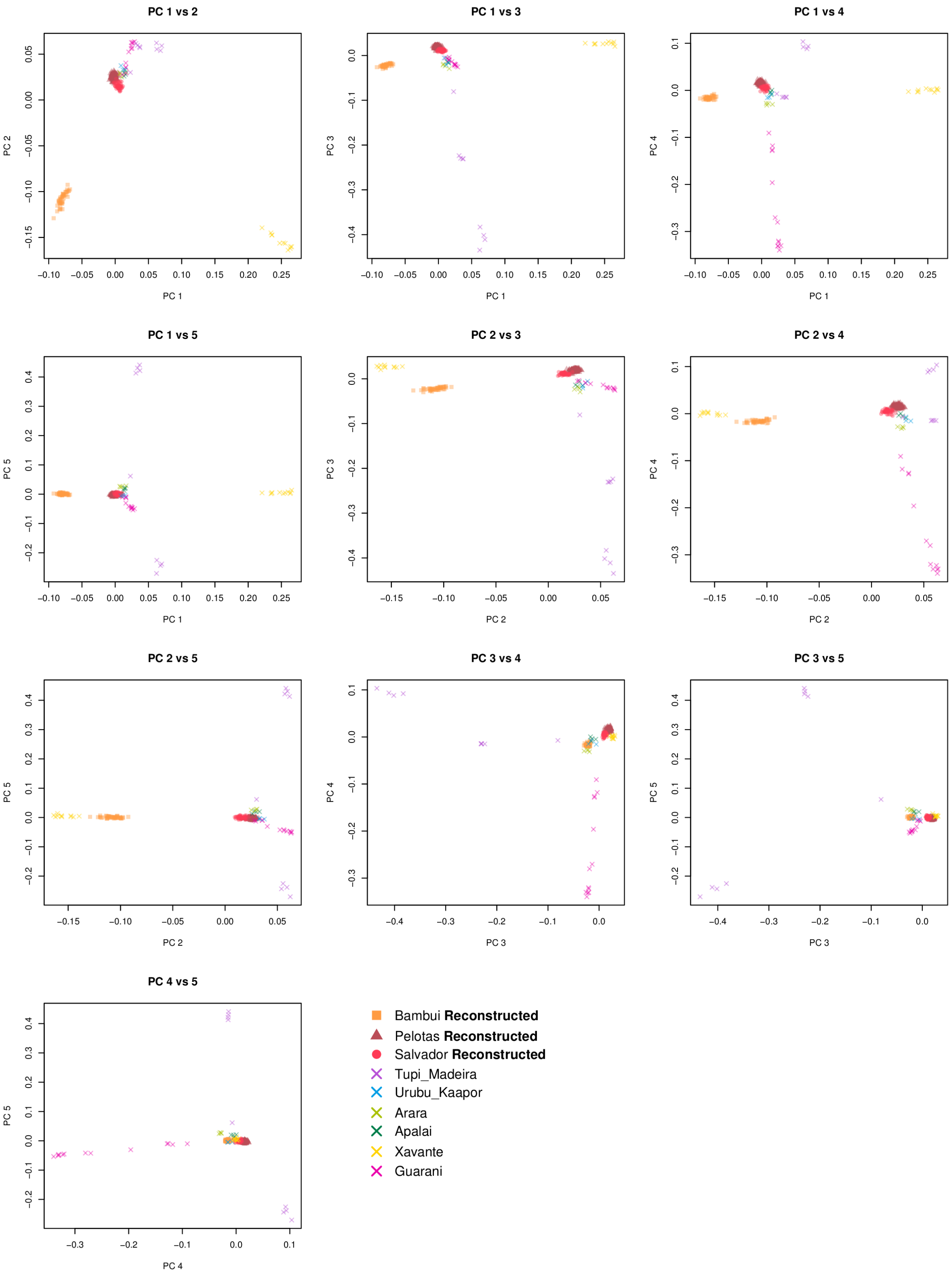

Figure S32. **Principal Component Analysis** based on the Chromopainter chunkcounts coancestry matrix of Native Americans, including reconstructed populations of Dataset B.

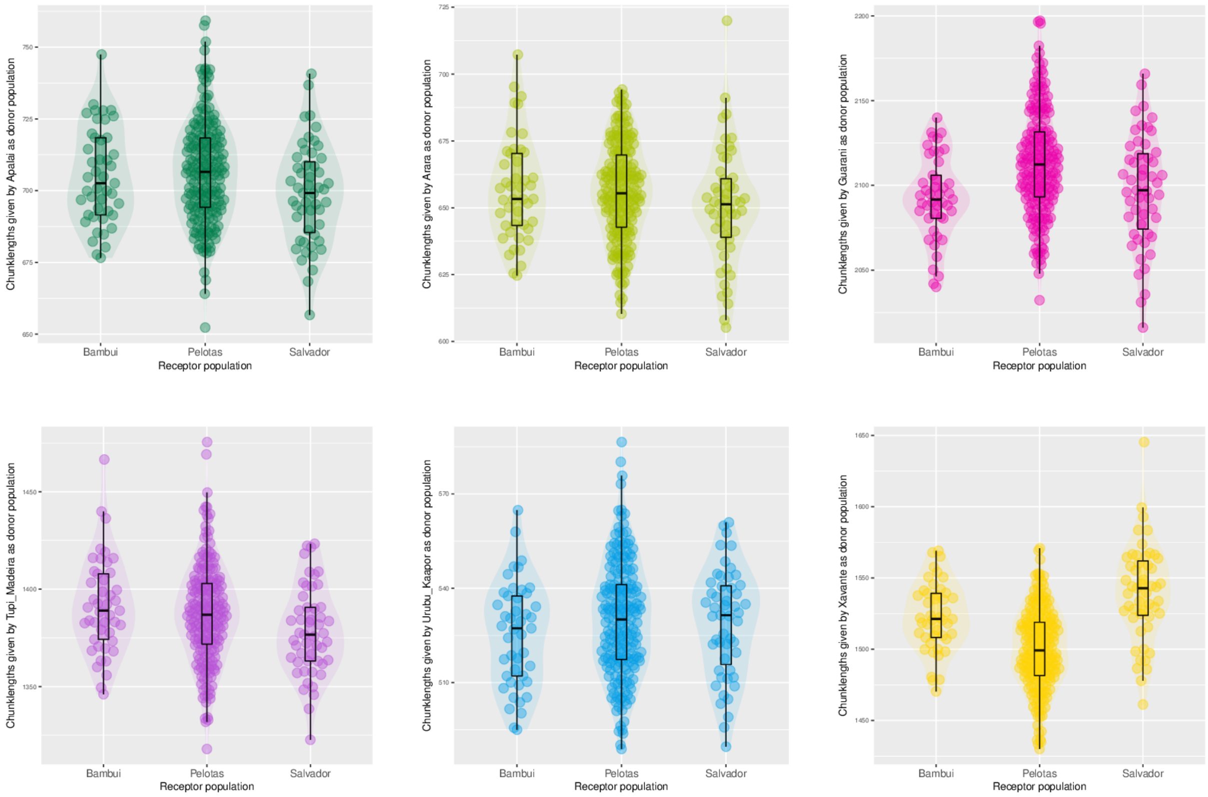

Figure S33. **Chunklengths given by donor populations** to Salvador, Bambui, and Pelotas reconstructed individuals. Significance of differences between recipient populations can be found on table S6. This figure corresponds to the complete Chromopainter analysis shown in main Figure 2.

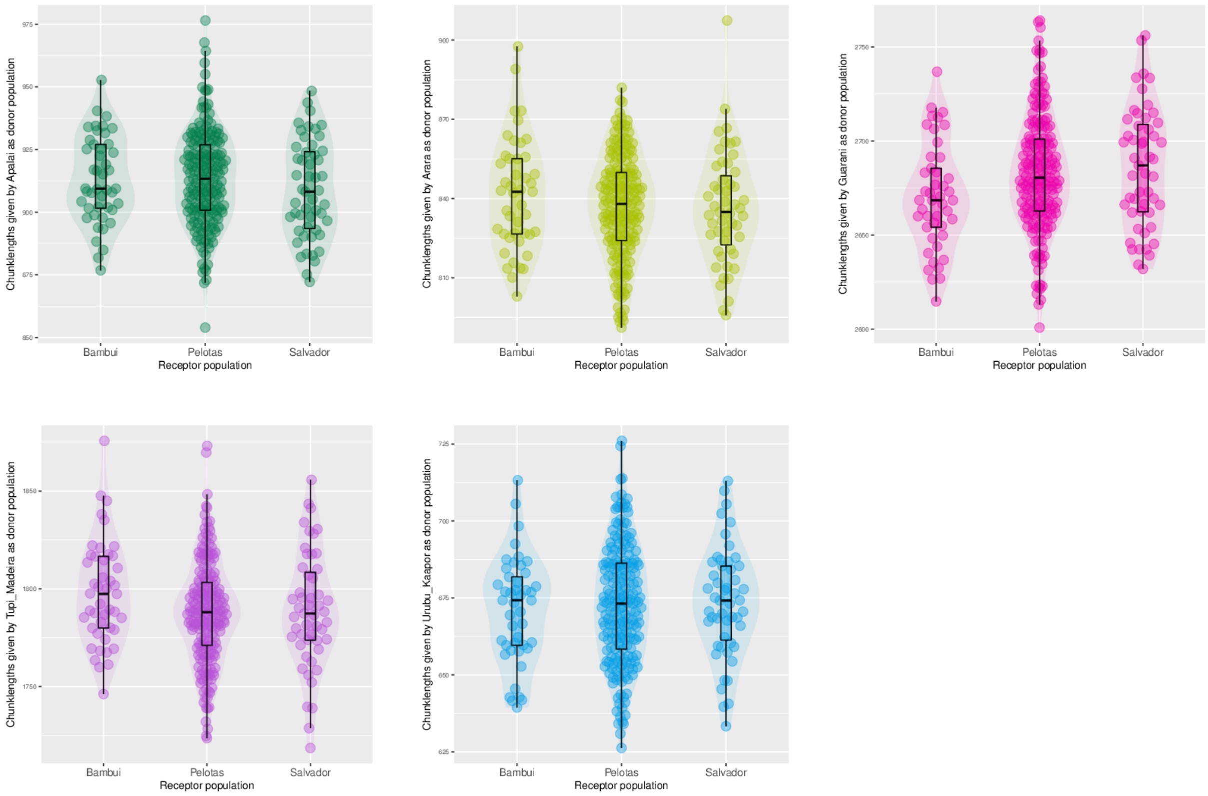

Figure S34. **Chunklengths given by donor populations without Guarani** to Salvador, Bambui, and Pelotas reconstructed individuals. Significance of differences between recipient populations can be found on table S7.

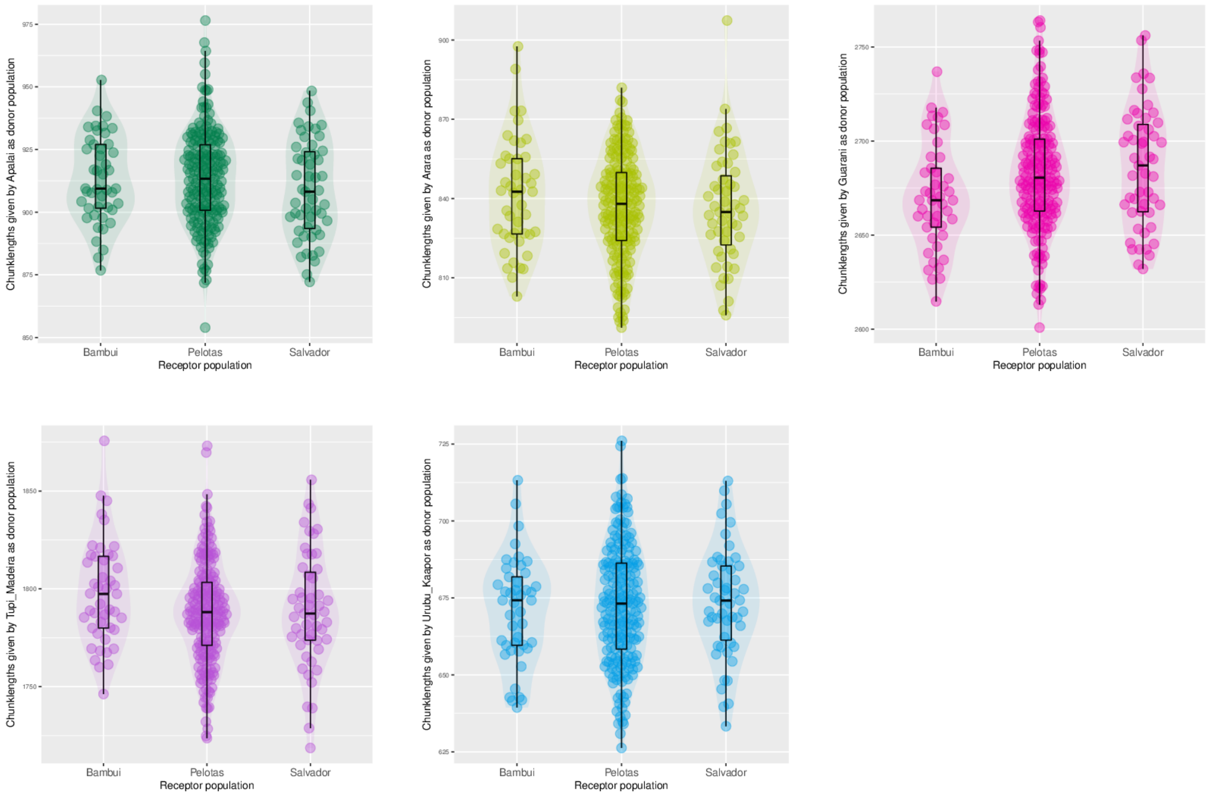

Figure S35. Chunklengths given by donor populations without Xavante to Salvador, Bambui, and Pelotas reconstructed individuals. Significance of differences between recipient populations can be found on table S8.

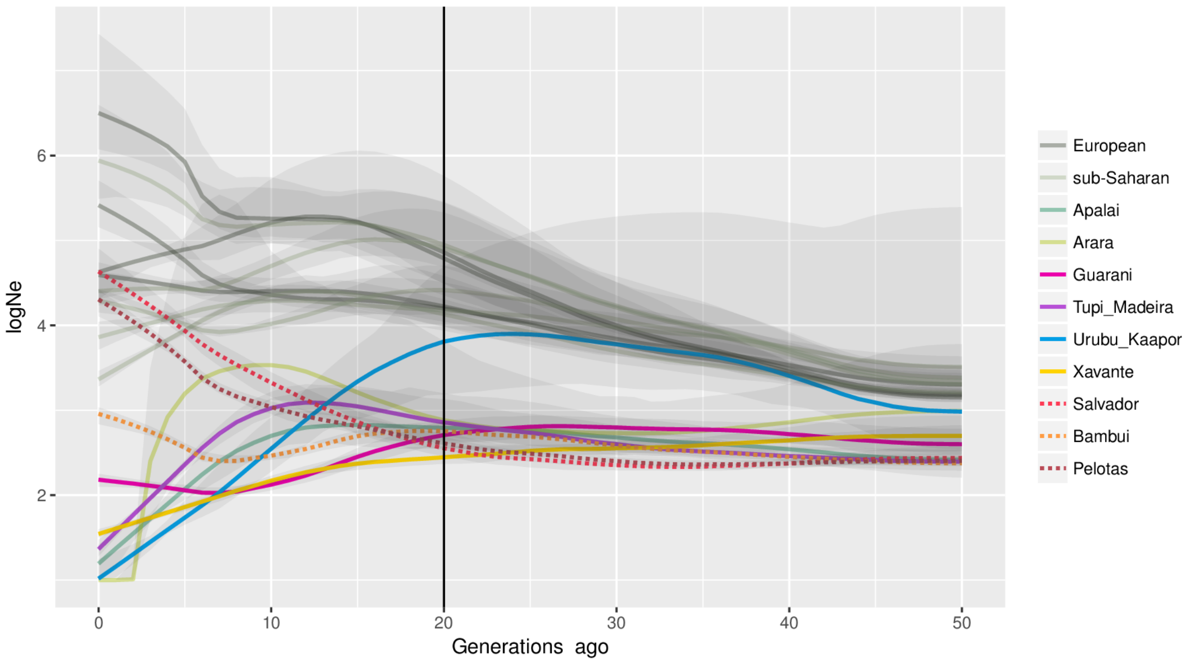

Figure S36. **Effective Population size (Ne)** expressed as log(Ne) obtained from IBD fragments with IBDseq and IBDNe from present to 50 generations ago, not filtered by confidence interval.

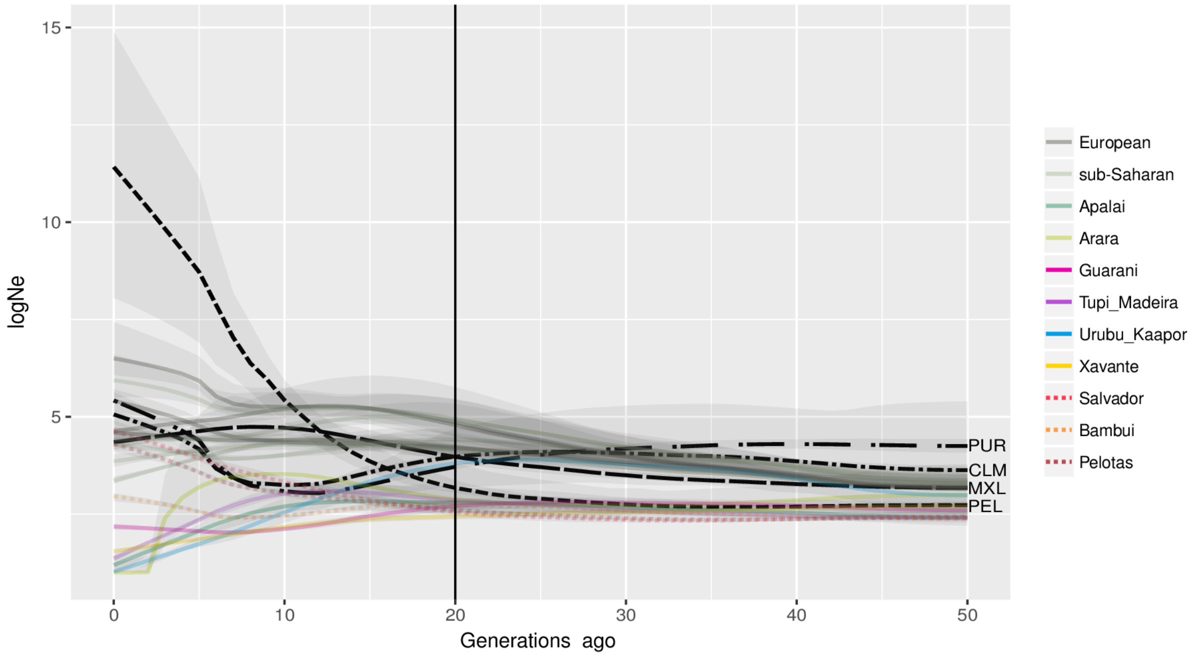

Figure S37. **Effective Population size (Ne)** expressed as log(Ne) obtained from IBD fragments with IBDseq and IBDNe from present to 50 generations ago, not filtered by confidence interval. In black and labeled next to the lines, the IBDNe of the admixed Americans of 1000genomes.

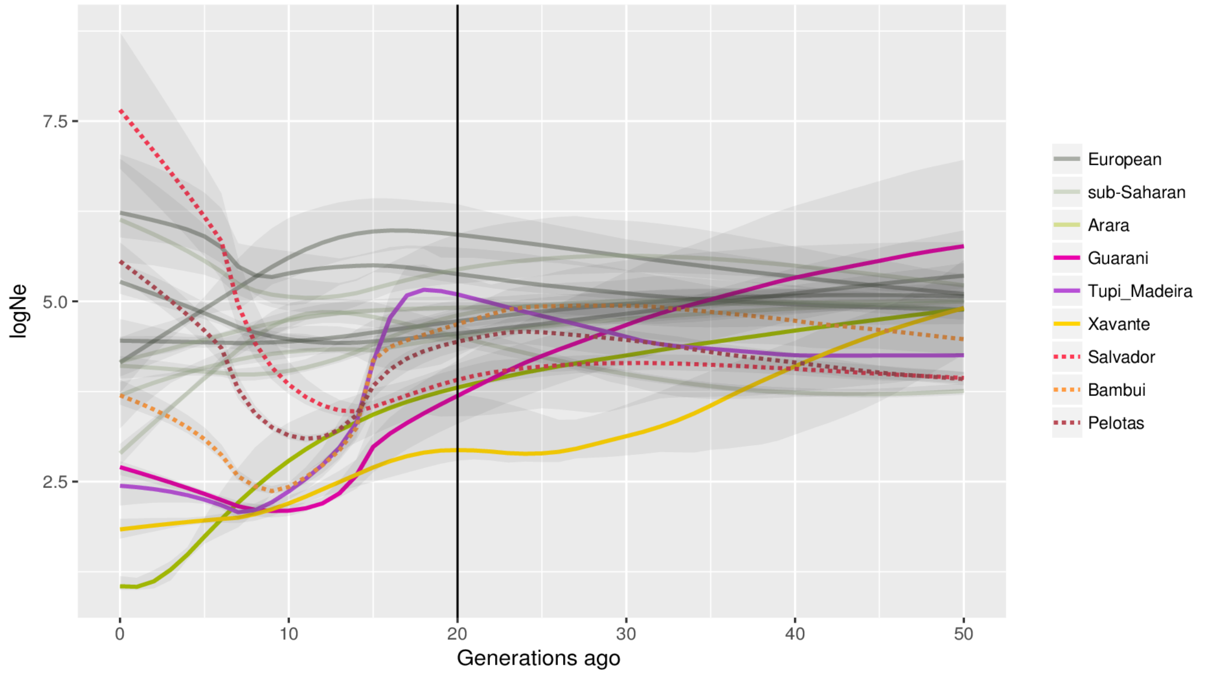

Figure S38. **Effective Population size (Ne)** expressed as log(Ne) obtained from IBD fragments with Refined IBD, merge IBD and IBDNe from present to 50 generations ago, filtered by a 95% confidence interval range of 2.5.

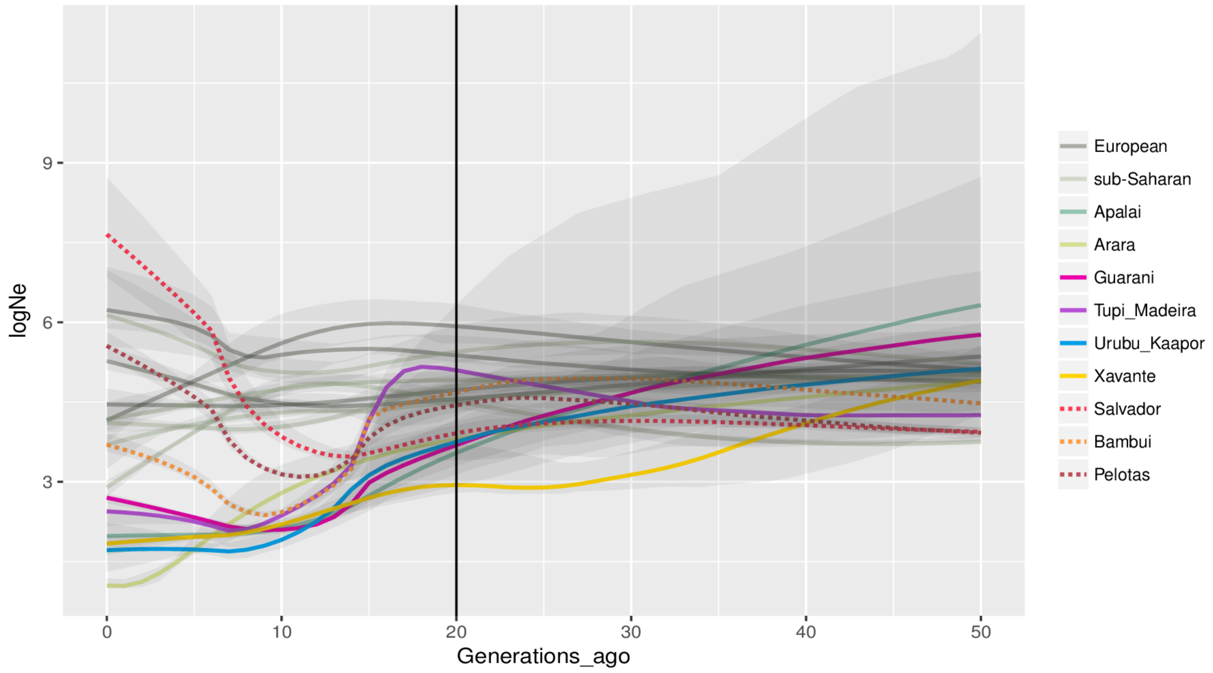

Figure S39. **Effective Population size (Ne)** expressed as log(Ne) obtained from IBD fragments with Refined IBD, merge IBD and IBDNe from present to 50 generations ago, not filtered by confidence interval.

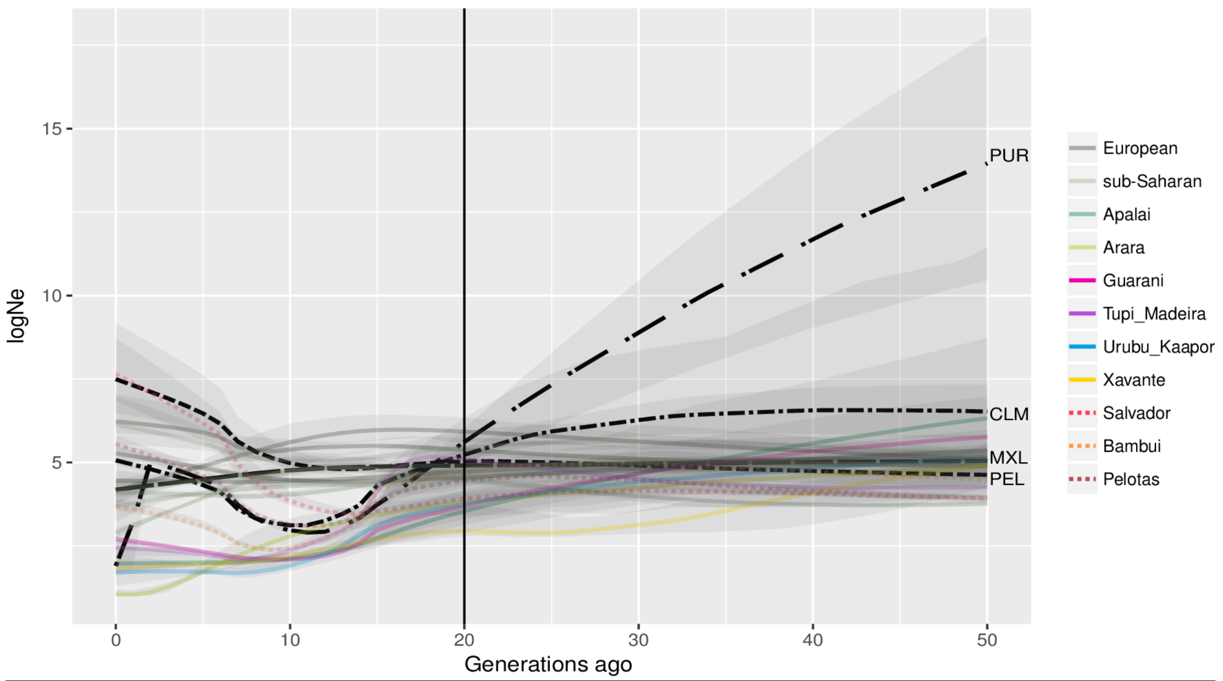

Figure S40. Effective Population size (Ne) expressed as log(Ne) obtained from IBD fragments with Refined IBD, merge IBD and IBDNe from present to 50 generations ago, not filtered by confidence interval, including admixed American populations from 1000 genomes.

Supplementary Tables

Dataset A		Dataset B	
n	Population	n	Population
4	Apalai	4	Apalai
4	Arara	4	Arara
7	Guarani_Ñandeva	7	Guarani_Ñandeva
9	Guarani_Kaiowá	9	Guarani_Kaiowá
5	Karitiana	5	Karitiana
4	Surui	4	Surui
3	Urubu_Kaapor	3	Urubu_Kaapor
11	Xavante	11	Xavante
1	Zoro	1	Zoro
1246	Salvador	51	Salvador Reconstructed
926	Bambui	45	Bambui Reconstructed
3653	Pelotas	197	Pelotas Reconstructed
99	CEU	99	CEU
99	ESN	99	ESN
91	GBR	91	GBR
107	IBS	107	IBS
107	TSI	107	TSI
113	GWD	113	GWD
99	LWK	99	LWK
85	MSL	85	MSL
108	YRI	108	YRI

Table S1. Individuals and populations analyzed. Dataset A (with current admixed Brazilians) and Dataset B (with reconstructed Native American Brazilians) used in the analyses.

Language classifications			Languages/populations
**Tupi Stock**	Tupi Guarani family	Guarani group	Guarani Kaiowá<br>Guarani Ñandevá
		Wayampi group	Urubu Kaapor
	Arikem branch		Karitiana
	Mondé branch		Surui
			Zoro
**Carib**	Central branch		Apalai
	South Amazonian branch		Arara
**Je**	Central-branch		Xavante

Table S2. Language classification of the populations sampled (Campbell and Grondona 2012)

Chromosome	SNPs
1	14727
2	15416
3	12995
4	10112
5	10130
6	10035
7	9076
8	8610
9	7754
10	9938
11	8758
12	8907
13	5967
14	6408
15	6213
16	6377
17	5034
18	5465
19	2947
20	290
21	2805
22	363
Total	168327

Table S3. **Number of SNPs by chromosome used in both DatasetA and DatasetB**

chr	Bambui	Pelotas	Salvador
1	110	425	107
2	101	449	126
3	98	428	118
4	108	461	112
5	115	450	109
6	123	443	118
7	98	412	116
8	110	454	119
9	108	442	132
10	123	447	122
11	124	433	120
12	108	461	112
13	116	464	109
14	106	463	117
15	107	433	**102**
16	118	449	110
17	106	460	119
18	114	431	122
19	116	449	120
20	142	450	117
21	93	**394**	117
22	**90**	432	106

Table S4. Reconstructed chromosomes per populations and autosome in the best iteration. Table related to figure S4. The numbers show the amount of chromosomes reconstructed above the threshold of 95% of non-missing positions. Highlighted cells show the autosome with less chromosomes reconstructed, which sets the total amount of chromosomes for the whole population.

Cluster	n	population	Cluster	n	population	Cluster	n	population	Cluster	n	population
1	8	Bambui_adm	22	13	YRI	58	5	YRI	107	3	MSL
1	32	Pelotas_adm	23	1	ESN	59	3	LWK	108	3	MSL
1	86	Salvador_adm	24	1	YRI	60	14	LWK	108	2	YRI
2	6	Bambui_adm	25	1	ESN	61	24	LWK	109	12	GWD
2	2	ESN	25	1	YRI	62	2	LWK	109	7	MSL
2	37	Pelotas_adm	26	1	YRI	63	1	LWK	110	284	Bambui_adm
2	16	Salvador_adm	27	2	ESN	64	6	LWK	110	48	Pelotas_adm
2	1	YRI	27	8	YRI	65	1	LWK	110	1	Salvador_adm
3	14	Bambui_adm	28	1	YRI	66	1	LWK	111	73	Bambui_adm
3	61	Pelotas_adm	29	2	ESN	67	1	LWK	111	2	Pelotas_adm
3	266	Salvador_adm	29	1	YRI	68	15	LWK	112	77	Bambui_adm
4	6	Bambui_adm	30	10	ESN	69	2	LWK	112	1	CEU
4	87	Pelotas_adm	30	12	YRI	70	3	LWK	112	79	Pelotas_adm
4	44	Salvador_adm	31	1	ESN	71	1	LWK	113	120	Bambui_adm
5	18	Bambui_adm	32	1	ESN	72	3	LWK	113	94	CEU
5	134	Pelotas_adm	33	2	ESN	73	3	LWK	113	89	GBR
5	38	Salvador_adm	34	1	ESN	74	1	LWK	113	107	IBS
6	4	Bambui_adm	35	5	ESN	75	1	LWK	113	2419	Pelotas_adm
6	33	Pelotas_adm	35	2	YRI	76	2	LWK	113	107	TSI
6	9	Salvador_adm	36	1	ESN	77	1	LWK	114	4	CEU
7	6	Bambui_adm	37	1	ESN	78	1	LWK	114	2	GBR
7	44	Pelotas_adm	38	7	ESN	79	1	LWK	114	18	Pelotas_adm
7	286	Salvador_adm	39	1	ESN	80	2	LWK	115	194	Bambui_adm
8	2	Pelotas_adm	40	8	ESN	81	10	LWK	115	278	Pelotas_adm
8	4	Salvador_adm	40	1	GWD	82	23	GWD	115	36	Salvador_adm
9	2	Pelotas_adm	40	19	YRI	83	1	GWD	116	4	Apalai
9	15	Salvador_adm	41	1	YRI	84	1	GWD	116	4	Arara
10	8	Bambui_adm	42	1	YRI	85	12	GWD	116	7	Guarani_GN
10	10	Pelotas_adm	43	1	YRI	86	1	GWD	116	10	Guarani_KW
10	40	Salvador_adm	45	1	YRI	87	6	GWD	116	5	Karitiana
11	24	Bambui_adm	46	1	YRI	88	10	GWD	116	4	Surui
11	94	Pelotas_adm	47	1	YRI	89	18	GWD	116	3	Urubu_Kaapor
11	239	Salvador_adm	48	1	ESN	90	11	GWD	116	11	Xavante
12	8	Bambui_adm	48	2	YRI	91	9	GWD	116	1	Zoro
12	35	Pelotas_adm	49	1	YRI	92	5	GWD			
12	26	Salvador_adm	50	1	YRI	93	17	MSL			
13	6	Bambui_adm	51	1	ESN	94	1	MSL			
13	67	Pelotas_adm	52	10	ESN	95	1	MSL			
13	11	Salvador_adm	52	1	MSL	96	1	MSL			
14	55	Bambui_adm	52	4	YRI	97	5	MSL			
14	149	Pelotas_adm	53	9	ESN	98	1	MSL			
14	128	Salvador_adm	53	1	MSL	99	1	MSL			
15	20	Pelotas_adm	53	4	YRI	100	4	MSL			
16	13	Bambui_adm	54	1	YRI	101	13	MSL			
17	19	ESN	55	1	ESN	102	1	GWD			
17	18	YRI	56	1	ESN	102	18	MSL			
18	1	ESN	56	2	YRI	103	1	GWD			
18	1	YRI	57	1	ESN	103	2	MSL			
19	1	ESN	58	2	Bambui_adm	104	1	MSL			
20	1	ESN	58	1	ESN	105	1	MSL			
21	1	YRI	58	2	Pelotas_adm	106	1	GWD			
22	6	ESN	58	1	Salvador_adm	106	4	MSL			

Table S5. **Consensus clusters for Dataset A** through the three seeds of FineStructure at height 1

Cluster	n	population
1	4	Arara
2	4	Apalai
2	3	Urubu_Kaapor
3	4	Surui
3	1	Zoro
4	5	Karitiana
5	11	Xavante
6	51	Salvador
7	8	Guarani_KW
8	7	Guarani_GN
8	2	Guarani_KW
9	197	Pelotas
10	45	Bambui
11	1	CEU
11	107	IBS
12	107	TSI
13	98	CEU
13	91	GBR
14	39	LWK
15	60	LWK
16	99	ESN
16	108	YRI
17	84	MSL
18	113	GWD
18	1	MSL

Table S6. **Consensus clusters for Dataset B** through the three seeds of FineStructure at height 4.

Donor	Bambui.Pelotas.pvalue	Bambui.Pelotas.bonferronip	Bambui.Pelotas.greater
Apalai	5.69E-01	1.00E+00	Pelotas
Arara	8.95E-01	1.00E+00	Bambui
Guarani	6.91E-05	1.24E-03	Pelotas
Tupi_Madeira	4.33E-01	1.00E+00	Bambui
Urubu_Kaapor	1.19E-01	1.00E+00	Pelotas
Xavante	2.38E-06	4.29E-05	Bambui
**Donor**	**Bambui.Salvador.pvalu**	**Bambui.Salvador.bonferronip**	**Bambui.Salvador.greater**
Apalai	1.21E-01	1.00E+00	Bambui
Arara	1.79E-01	1.00E+00	Bambui
Guarani	3.47E-01	1.00E+00	Salvador
Tupi_Madeira	1.42E-02	2.56E-01	Bambui
Urubu_Kaapor	2.58E-01	1.00E+00	Salvador
Xavante	4.60E-03	8.27E-02	Salvador
**Donor**	**Salvador.Pelotas.pvalu**	**Salvador.Pelotas.bonferronip**	**Salvador.Pelotas.greater**
Apalai	8.42E-03	1.52E-01	Pelotas
Arara	5.21E-02	9.37E-01	Pelotas
Guarani	5.41E-03	9.74E-02	Pelotas
Tupi_Madeira	1.67E-02	3.00E-01	Pelotas
Urubu_Kaapor	8.54E-01	1.00E+00	Pelotas
Xavante	1.93E-12	3.48E-11	Salvador

Table S7. **Wilcoxon test for chunklengths donor analysis** of Figure 2 and S32 comparing the amount of chunklengths given by the donor population between the recipient populations: Salvador, Bambui and Pelotas.

Donor	Bambui.Pelotas.pvalue	Bambui.Pelotas.bonferronip	Bambui.Pelotas.greater
Apalai	2.15E-01	1.00E+00	Pelotas
Arara	8.28E-02	1.00E+00	Pelotas
Tupi_Madeira	4.44E-01	1.00E+00	Pelotas
Urubu_Kaapor	4.43E-02	6.65E-01	Pelotas
Xavante	8.76E-05	1.31E-03	Bambui
**Donor**	**Bambui.Salvador.pvalue**	**Bambui.Salvador.bonferronip**	**Bambui.Salvador.greater**
Apalai	1.08E-01	1.00E+00	Bambui
Arara	5.97E-01	1.00E+00	Bambui
Tupi_Madeira	1.06E-02	1.59E-01	Bambui
Urubu_Kaapor	1.34E-01	1.00E+00	Salvador
Xavante	5.90E-04	8.85E-03	Salvador
**Donor**	**Salvador.Pelotas.pvalue**	**Salvador.Pelotas.bonferronip**	**Salvador.Pelotas.greater**
Apalai	7.99E-04	1.20E-02	Pelotas
Arara	2.07E-02	3.11E-01	Pelotas
Tupi_Madeira	1.40E-04	2.10E-03	Pelotas
Urubu_Kaapor	7.84E-01	1.00E+00	Pelotas
Xavante	2.09E-12	3.14E-11	Salvador

Table S8. **Wilcoxon test for chunklengths donor analysis without Guarani** as donor corresponding to Figure S33 comparing the amount of chunklengths given by the donor population between the recipient populations: Salvador, Bambui and Pelotas.

Donor	Bambui.Pelotas.pvalue	Bambui.Pelotas.bonferronip	**Bambui.Pelotas.greater**
Apalai	0.9811701067	1	Pelotas
Arara	0.3343844238	1	Bambui
Guarani	0.011024182	0.1653627297	Pelotas
Tupi_Madeira	0.0384624487	0.5769367311	Bambui
Urubu_Kaapor	0.6135012449	1	Pelotas
**Donor**	**Bambui.Salvador.pvalue**	**Bambui.Salvador.bonferronip**	**Bambui.Salvador.greater**
Apalai	0.2581959173	1	Bambui
Arara	0.2644300495	1	Bambui
Guarani	0.0276233007	0.4143495103	Salvador
Tupi_Madeira	0.1674964741	1	Bambui
Urubu_Kaapor	0.5277720929	1	Salvador
**Donor**	**Salvador.Pelotas.pvalue**	**Salvador.Pelotas.bonferronip**	**Salvador.Pelotas.greater**
Apalai	0.1643037706	1	Pelotas
Arara	0.6113737487	1	Pelotas
Guarani	0.5411662903	1	Salvador
Tupi_Madeira	0.8283452777	1	Salvador
Urubu_Kaapor	0.7243784048	1	Salvador

Table S9. Wilcoxon test for chunklengths donor analysis without Xavante as donor corresponding to Figure S34 comparing the amount of chunklengths given by the donor population between the recipient populations: Salvador, Bambui and Pelotas.

Recipient/Donor	Apalai	Arara	Guarani	Tupi_Madeira	Urubu_Kaapor	Xavante
Bambui	0.3365	0.0674	0.1806	0.1566	0.1040	0.1550
Pelotas	0.3731	0.0570	0.1860	0.1424	0.1009	0.1406
Salvador	0.3318	0.0581	0.1718	0.1599	0.1147	0.1636

Table S10. **Non-negative least squared** algorithm applied to the chunklengths donors matrix corresponding to Figure 2 and Figure S32.

Population	Genetic diversity	Gen. diversity (%)
Apalai	0.2209	68.9783
Arara	0.2070	64.6261
Bambui	0.2319	72.3958
Guarani	0.2258	70.4993
Pelotas	0.2357	73.5978
Salvador	0.2349	73.3562
Tupi_Madeira	0.2225	69.4845
Urubu_Kaapor	0.2061	64.3653
Xavante	0.2141	66.8559
NativeAmerican	0.2341	73.0856
Reconstructed	0.2363	73.7851
MSL	0.3069	95.8097
YRI	0.3076	96.0350
GWD	0.3074	95.9630
ESN	0.3067	95.7624
LWK	0.3106	96.9653
CEU	0.2941	91.8327
GBR	0.2937	91.7064
IBS	0.2962	92.4946
TSI	0.2958	92.3659
CLM	0.3059	95.5182
PUR	0.3109	97.0637
PEL	0.2721	84.9467
MXL	0.2963	92.5070
ALL	0.3203	100.0000

Table S11. **Genetic diversity** of each population as the average of all SNPs of the dataset and the relative value (in percentage) to the genetic diversity between all the individuals of the dataset. Native American includes all individuals of Native American populations, without the reconstructed individuals. Reconstructed include all the reconstructed individuals of Bambui, Pelotas and Salvador. Genetic Diversity has been computed per SNP position within each population through vcftools(Danecek et al. 2011) with --site-pi flag.

Population 1	Population 2	mean1	mean2	pvalue	bonferronip	sign	greater
Apalai	Arara	0.2209	0.2070	1.04E-73	1.15E-70	YES	Apalai
Apalai	Bambui	0.2209	0.2319	3.13E-144	3.46E-141	YES	Bambui
Apalai	Guarani	0.2209	0.2258	2.06E-13	2.27E-10	YES	Guarani
Apalai	Pelotas	0.2209	0.2357	0.00E+00	0.00E+00	YES	Pelotas
Apalai	Salvador	0.2209	0.2349	9.10E-243	1.00E-239	YES	Salvador
Apalai	Tupi_Madeira	0.2209	0.2225	7.45E-19	8.22E-16	YES	Tupi_Madeira
Apalai	Urubu_Kaapor	0.2209	0.2061	3.77E-190	4.17E-187	YES	Apalai
Apalai	Xavante	0.2209	0.2141	9.46E-63	1.04E-59	YES	Apalai
Apalai	NativeAmerican	0.2209	0.2341	7.30E-171	8.06E-168	YES	NativeAmerican
Apalai	Reconstructed	0.2209	0.2363	0.00E+00	0.00E+00	YES	Reconstructed
Apalai	MSL	0.2209	0.3069	0.00E+00	0.00E+00	YES	MSL
Apalai	YRI	0.2209	0.3076	0.00E+00	0.00E+00	YES	YRI
Apalai	GWD	0.2209	0.3074	0.00E+00	0.00E+00	YES	GWD
Apalai	ESN	0.2209	0.3067	0.00E+00	0.00E+00	YES	ESN
Apalai	LWK	0.2209	0.3106	0.00E+00	0.00E+00	YES	LWK
Apalai	CEU	0.2209	0.2941	0.00E+00	0.00E+00	YES	CEU
Apalai	GBR	0.2209	0.2937	0.00E+00	0.00E+00	YES	GBR
Apalai	IBS	0.2209	0.2962	0.00E+00	0.00E+00	YES	IBS
Apalai	TSI	0.2209	0.2958	0.00E+00	0.00E+00	YES	TSI
Apalai	CLM	0.2209	0.3059	0.00E+00	0.00E+00	YES	CLM
Apalai	PUR	0.2209	0.3109	0.00E+00	0.00E+00	YES	PUR
Apalai	PEL	0.2209	0.2721	0.00E+00	0.00E+00	YES	PEL
Apalai	MXL	0.2209	0.2963	0.00E+00	0.00E+00	YES	MXL
Arara	Bambui	0.2070	0.2319	0.00E+00	0.00E+00	YES	Bambui
Arara	Guarani	0.2070	0.2258	2.41E-172	2.66E-169	YES	Guarani
Arara	Pelotas	0.2070	0.2357	0.00E+00	0.00E+00	YES	Pelotas
Arara	Salvador	0.2070	0.2349	0.00E+00	0.00E+00	YES	Salvador
Arara	Tupi_Madeira	0.2070	0.2225	9.26E-32	1.02E-28	YES	Tupi_Madeira
Arara	Urubu_Kaapor	0.2070	0.2061	3.58E-36	3.95E-33	YES	Arara
Arara	Xavante	0.2070	0.2141	2.58E-06	2.84E-03	YES	Xavante
Arara	NativeAmerican	0.2070	0.2341	0.00E+00	0.00E+00	YES	NativeAmerican
Arara	Reconstructed	0.2070	0.2363	0.00E+00	0.00E+00	YES	Reconstructed
Arara	MSL	0.2070	0.3069	0.00E+00	0.00E+00	YES	MSL
Arara	YRI	0.2070	0.3076	0.00E+00	0.00E+00	YES	YRI
Arara	GWD	0.2070	0.3074	0.00E+00	0.00E+00	YES	GWD
Arara	ESN	0.2070	0.3067	0.00E+00	0.00E+00	YES	ESN
Arara	LWK	0.2070	0.3106	0.00E+00	0.00E+00	YES	LWK
Arara	CEU	0.2070	0.2941	0.00E+00	0.00E+00	YES	CEU
Arara	GBR	0.2070	0.2937	0.00E+00	0.00E+00	YES	GBR
Arara	IBS	0.2070	0.2962	0.00E+00	0.00E+00	YES	IBS
Arara	TSI	0.2070	0.2958	0.00E+00	0.00E+00	YES	TSI
Arara	CLM	0.2070	0.3059	0.00E+00	0.00E+00	YES	CLM
Arara	PUR	0.2070	0.3109	0.00E+00	0.00E+00	YES	PUR
Arara	PEL	0.2070	0.2721	0.00E+00	0.00E+00	YES	PEL
Arara	MXL	0.2070	0.2963	0.00E+00	0.00E+00	YES	MXL
Bambui	Guarani	0.2319	0.2258	2.15E-17	2.37E-14	YES	Bambui
Bambui	Pelotas	0.2319	0.2357	3.19E-20	3.53E-17	YES	Pelotas
Bambui	Salvador	0.2319	0.2349	3.12E-07	3.44E-04	YES	Salvador
Bambui	Tupi_Madeira	0.2319	0.2225	7.85E-53	8.66E-50	YES	Bambui
Bambui	Urubu_Kaapor	0.2319	0.2061	0.00E+00	0.00E+00	YES	Bambui
Bambui	Xavante	0.2319	0.2141	1.33E-159	1.47E-156	YES	Bambui
Bambui	NativeAmerican	0.2319	0.2341	1.23E-03	1.00E+00	NO	NativeAmerican
Bambui	Reconstructed	0.2319	0.2363	2.27E-41	2.50E-38	YES	Reconstructed
Bambui	MSL	0.2319	0.3069	0.00E+00	0.00E+00	YES	MSL
Bambui	YRI	0.2319	0.3076	0.00E+00	0.00E+00	YES	YRI
Bambui	GWD	0.2319	0.3074	0.00E+00	0.00E+00	YES	GWD
Bambui	ESN	0.2319	0.3067	0.00E+00	0.00E+00	YES	ESN
Bambui	LWK	0.2319	0.3106	0.00E+00	0.00E+00	YES	LWK
Bambui	CEU	0.2319	0.2941	0.00E+00	0.00E+00	YES	CEU
Bambui	GBR	0.2319	0.2937	0.00E+00	0.00E+00	YES	GBR
Bambui	IBS	0.2319	0.2962	0.00E+00	0.00E+00	YES	IBS
Bambui	TSI	0.2319	0.2958	0.00E+00	0.00E+00	YES	TSI
Bambui	CLM	0.2319	0.3059	0.00E+00	0.00E+00	YES	CLM
Bambui	PUR	0.2319	0.3109	0.00E+00	0.00E+00	YES	PUR
Bambui	PEL	0.2319	0.2721	0.00E+00	0.00E+00	YES	PEL
Bambui	MXL	0.2319	0.2963	0.00E+00	0.00E+00	YES	MXL
Guarani	Pelotas	0.2258	0.2357	9.11E-116	1.01E-112	YES	Pelotas
Guarani	Salvador	0.2258	0.2349	3.55E-44	3.92E-41	YES	Salvador
Guarani	Tupi_Madeira	0.2258	0.2225	4.51E-12	4.98E-09	YES	Guarani
Guarani	Urubu_Kaapor	0.2258	0.2061	8.15E-135	9.00E-132	YES	Guarani
Guarani	Xavante	0.2258	0.2141	4.16E-64	4.59E-61	YES	Guarani
Guarani	NativeAmerican	0.2258	0.2341	1.32E-23	1.46E-20	YES	NativeAmerican
Guarani	Reconstructed	0.2258	0.2363	6.89E-172	7.60E-169	YES	Reconstructed
Guarani	MSL	0.2258	0.3069	0.00E+00	0.00E+00	YES	MSL
Guarani	YRI	0.2258	0.3076	0.00E+00	0.00E+00	YES	YRI
Guarani	GWD	0.2258	0.3074	0.00E+00	0.00E+00	YES	GWD
Guarani	ESN	0.2258	0.3067	0.00E+00	0.00E+00	YES	ESN
Guarani	LWK	0.2258	0.3106	0.00E+00	0.00E+00	YES	LWK
Guarani	CEU	0.2258	0.2941	0.00E+00	0.00E+00	YES	CEU
Guarani	GBR	0.2258	0.2937	0.00E+00	0.00E+00	YES	GBR
Guarani	IBS	0.2258	0.2962	0.00E+00	0.00E+00	YES	IBS
Guarani	TSI	0.2258	0.2958	0.00E+00	0.00E+00	YES	TSI
Guarani	CLM	0.2258	0.3059	0.00E+00	0.00E+00	YES	CLM
Guarani	PUR	0.2258	0.3109	0.00E+00	0.00E+00	YES	PUR
Guarani	PEL	0.2258	0.2721	0.00E+00	0.00E+00	YES	PEL
Guarani	MXL	0.2258	0.2963	0.00E+00	0.00E+00	YES	MXL
Pelotas	Salvador	0.2357	0.2349	2.88E-03	1.00E+00	NO	Pelotas
Pelotas	Tupi_Madeira	0.2357	0.2225	8.49E-169	9.37E-166	YES	Pelotas
Pelotas	Urubu_Kaapor	0.2357	0.2061	0.00E+00	0.00E+00	YES	Pelotas
Pelotas	Xavante	0.2357	0.2141	0.00E+00	0.00E+00	YES	Pelotas
Pelotas	NativeAmerican	0.2357	0.2341	8.49E-13	9.37E-10	YES	Pelotas
Pelotas	Reconstructed	0.2357	0.2363	3.60E-03	1.00E+00	NO	Reconstructed
Pelotas	MSL	0.2357	0.3069	0.00E+00	0.00E+00	YES	MSL
Pelotas	YRI	0.2357	0.3076	0.00E+00	0.00E+00	YES	YRI
Pelotas	GWD	0.2357	0.3074	0.00E+00	0.00E+00	YES	GWD
Pelotas	ESN	0.2357	0.3067	0.00E+00	0.00E+00	YES	ESN
Pelotas	LWK	0.2357	0.3106	0.00E+00	0.00E+00	YES	LWK
Pelotas	CEU	0.2357	0.2941	0.00E+00	0.00E+00	YES	CEU
Pelotas	GBR	0.2357	0.2937	0.00E+00	0.00E+00	YES	GBR
Pelotas	IBS	0.2357	0.2962	0.00E+00	0.00E+00	YES	IBS
Pelotas	TSI	0.2357	0.2958	0.00E+00	0.00E+00	YES	TSI
Pelotas	CLM	0.2357	0.3059	0.00E+00	0.00E+00	YES	CLM
Pelotas	PUR	0.2357	0.3109	0.00E+00	0.00E+00	YES	PUR
Pelotas	PEL	0.2357	0.2721	0.00E+00	0.00E+00	YES	PEL
Pelotas	MXL	0.2357	0.2963	0.00E+00	0.00E+00	YES	MXL
Salvador	Tupi_Madeira	0.2349	0.2225	1.15E-105	1.27E-102	YES	Salvador
Salvador	Urubu_Kaapor	0.2349	0.2061	0.00E+00	0.00E+00	YES	Salvador
Salvador	Xavante	0.2349	0.2141	6.74E-261	7.44E-258	YES	Salvador
Salvador	NativeAmerican	0.2349	0.2341	1.54E-03	1.00E+00	NO	Salvador
Salvador	Reconstructed	0.2349	0.2363	4.63E-12	5.12E-09	YES	Reconstructed
Salvador	MSL	0.2349	0.3069	0.00E+00	0.00E+00	YES	MSL
Salvador	YRI	0.2349	0.3076	0.00E+00	0.00E+00	YES	YRI
Salvador	GWD	0.2349	0.3074	0.00E+00	0.00E+00	YES	GWD

Salvador	ESN	0.2349	0.3067	0.00E+00	0.00E+00	YES	ESN
Salvador	LWK	0.2349	0.3106	0.00E+00	0.00E+00	YES	LWK
Salvador	CEU	0.2349	0.2941	0.00E+00	0.00E+00	YES	CEU
Salvador	GBR	0.2349	0.2937	0.00E+00	0.00E+00	YES	GBR
Salvador	IBS	0.2349	0.2962	0.00E+00	0.00E+00	YES	IBS
Salvador	TSI	0.2349	0.2958	0.00E+00	0.00E+00	YES	TSI
Salvador	CLM	0.2349	0.3059	0.00E+00	0.00E+00	YES	CLM
Salvador	PUR	0.2349	0.3109	0.00E+00	0.00E+00	YES	PUR
Salvador	PEL	0.2349	0.2721	0.00E+00	0.00E+00	YES	PEL
Salvador	MXL	0.2349	0.2963	0.00E+00	0.00E+00	YES	MXL
Tupi_Madeira	Urubu_Kaapor	0.2225	0.2061	1.20E-63	1.33E-60	YES	Tupi_Madeira
Tupi_Madeira	Xavante	0.2225	0.2141	4.99E-35	5.51E-32	YES	Tupi_Madeira
Tupi_Madeira	NativeAmerican	0.2225	0.2341	8.90E-63	9.83E-60	YES	NativeAmerican
Tupi_Madeira	Reconstructed	0.2225	0.2363	2.49E-252	2.74E-249	YES	Reconstructed
Tupi_Madeira	MSL	0.2225	0.3069	0.00E+00	0.00E+00	YES	MSL
Tupi_Madeira	YRI	0.2225	0.3076	0.00E+00	0.00E+00	YES	YRI
Tupi_Madeira	GWD	0.2225	0.3074	0.00E+00	0.00E+00	YES	GWD
Tupi_Madeira	ESN	0.2225	0.3067	0.00E+00	0.00E+00	YES	ESN
Tupi_Madeira	LWK	0.2225	0.3106	0.00E+00	0.00E+00	YES	LWK
Tupi_Madeira	CEU	0.2225	0.2941	0.00E+00	0.00E+00	YES	CEU
Tupi_Madeira	GBR	0.2225	0.2937	0.00E+00	0.00E+00	YES	GBR
Tupi_Madeira	IBS	0.2225	0.2962	0.00E+00	0.00E+00	YES	IBS
Tupi_Madeira	TSI	0.2225	0.2958	0.00E+00	0.00E+00	YES	TSI
Tupi_Madeira	CLM	0.2225	0.3059	0.00E+00	0.00E+00	YES	CLM
Tupi_Madeira	PUR	0.2225	0.3109	0.00E+00	0.00E+00	YES	PUR
Tupi_Madeira	PEL	0.2225	0.2721	0.00E+00	0.00E+00	YES	PEL
Tupi_Madeira	MXL	0.2225	0.2963	0.00E+00	0.00E+00	YES	MXL
Urubu_Kaapor	Xavante	0.2061	0.2141	2.61E-23	2.88E-20	YES	Xavante
Urubu_Kaapor	NativeAmerican	0.2061	0.2341	0.00E+00	0.00E+00	YES	NativeAmerican
Urubu_Kaapor	Reconstructed	0.2061	0.2363	0.00E+00	0.00E+00	YES	Reconstructed
Urubu_Kaapor	MSL	0.2061	0.3069	0.00E+00	0.00E+00	YES	MSL
Urubu_Kaapor	YRI	0.2061	0.3076	0.00E+00	0.00E+00	YES	YRI
Urubu_Kaapor	GWD	0.2061	0.3074	0.00E+00	0.00E+00	YES	GWD
Urubu_Kaapor	ESN	0.2061	0.3067	0.00E+00	0.00E+00	YES	ESN
Urubu_Kaapor	LWK	0.2061	0.3106	0.00E+00	0.00E+00	YES	LWK
Urubu_Kaapor	CEU	0.2061	0.2941	0.00E+00	0.00E+00	YES	CEU
Urubu_Kaapor	GBR	0.2061	0.2937	0.00E+00	0.00E+00	YES	GBR
Urubu_Kaapor	IBS	0.2061	0.2962	0.00E+00	0.00E+00	YES	IBS
Urubu_Kaapor	TSI	0.2061	0.2958	0.00E+00	0.00E+00	YES	TSI
Urubu_Kaapor	CLM	0.2061	0.3059	0.00E+00	0.00E+00	YES	CLM
Urubu_Kaapor	PUR	0.2061	0.3109	0.00E+00	0.00E+00	YES	PUR
Urubu_Kaapor	PEL	0.2061	0.2721	0.00E+00	0.00E+00	YES	PEL
Urubu_Kaapor	MXL	0.2061	0.2963	0.00E+00	0.00E+00	YES	MXL
Xavante	NativeAmerican	0.2141	0.2341	1.45E-157	1.60E-154	YES	NativeAmerican
Xavante	Reconstructed	0.2141	0.2363	0.00E+00	0.00E+00	YES	Reconstructed
Xavante	MSL	0.2141	0.3069	0.00E+00	0.00E+00	YES	MSL
Xavante	YRI	0.2141	0.3076	0.00E+00	0.00E+00	YES	YRI
Xavante	GWD	0.2141	0.3074	0.00E+00	0.00E+00	YES	GWD
Xavante	ESN	0.2141	0.3067	0.00E+00	0.00E+00	YES	ESN
Xavante	LWK	0.2141	0.3106	0.00E+00	0.00E+00	YES	LWK
Xavante	CEU	0.2141	0.2941	0.00E+00	0.00E+00	YES	CEU
Xavante	GBR	0.2141	0.2937	0.00E+00	0.00E+00	YES	GBR
Xavante	IBS	0.2141	0.2962	0.00E+00	0.00E+00	YES	IBS
Xavante	TSI	0.2141	0.2958	0.00E+00	0.00E+00	YES	TSI
Xavante	CLM	0.2141	0.3059	0.00E+00	0.00E+00	YES	CLM
Xavante	PUR	0.2141	0.3109	0.00E+00	0.00E+00	YES	PUR
Xavante	PEL	0.2141	0.2721	0.00E+00	0.00E+00	YES	PEL
Xavante	MXL	0.2141	0.2963	0.00E+00	0.00E+00	YES	MXL
NativeAmerican	Reconstructed	0.2341	0.2363	1.32E-31	1.46E-28	YES	Reconstructed
NativeAmerican	MSL	0.2341	0.3069	0.00E+00	0.00E+00	YES	MSL
NativeAmerican	YRI	0.2341	0.3076	0.00E+00	0.00E+00	YES	YRI
NativeAmerican	GWD	0.2341	0.3074	0.00E+00	0.00E+00	YES	GWD
NativeAmerican	ESN	0.2341	0.3067	0.00E+00	0.00E+00	YES	ESN
NativeAmerican	LWK	0.2341	0.3106	0.00E+00	0.00E+00	YES	LWK
NativeAmerican	CEU	0.2341	0.2941	0.00E+00	0.00E+00	YES	CEU
NativeAmerican	GBR	0.2341	0.2937	0.00E+00	0.00E+00	YES	GBR
NativeAmerican	IBS	0.2341	0.2962	0.00E+00	0.00E+00	YES	IBS
NativeAmerican	TSI	0.2341	0.2958	0.00E+00	0.00E+00	YES	TSI
NativeAmerican	CLM	0.2341	0.3059	0.00E+00	0.00E+00	YES	CLM
NativeAmerican	PUR	0.2341	0.3109	0.00E+00	0.00E+00	YES	PUR
NativeAmerican	PEL	0.2341	0.2721	0.00E+00	0.00E+00	YES	PEL
NativeAmerican	MXL	0.2341	0.2963	0.00E+00	0.00E+00	YES	MXL
Reconstructed	MSL	0.2363	0.3069	0.00E+00	0.00E+00	YES	MSL
Reconstructed	YRI	0.2363	0.3076	0.00E+00	0.00E+00	YES	YRI
Reconstructed	GWD	0.2363	0.3074	0.00E+00	0.00E+00	YES	GWD
Reconstructed	ESN	0.2363	0.3067	0.00E+00	0.00E+00	YES	ESN
Reconstructed	LWK	0.2363	0.3106	0.00E+00	0.00E+00	YES	LWK
Reconstructed	CEU	0.2363	0.2941	0.00E+00	0.00E+00	YES	CEU
Reconstructed	GBR	0.2363	0.2937	0.00E+00	0.00E+00	YES	GBR
Reconstructed	IBS	0.2363	0.2962	0.00E+00	0.00E+00	YES	IBS
Reconstructed	TSI	0.2363	0.2958	0.00E+00	0.00E+00	YES	TSI
Reconstructed	CLM	0.2363	0.3059	0.00E+00	0.00E+00	YES	CLM
Reconstructed	PUR	0.2363	0.3109	0.00E+00	0.00E+00	YES	PUR
Reconstructed	PEL	0.2363	0.2721	0.00E+00	0.00E+00	YES	PEL
Reconstructed	MXL	0.2363	0.2963	0.00E+00	0.00E+00	YES	MXL
MSL	YRI	0.3069	0.3076	8.66E-01	1.00E+00	NO	YRI
MSL	GWD	0.3069	0.3074	6.92E-01	1.00E+00	NO	GWD
MSL	ESN	0.3069	0.3067	6.90E-01	1.00E+00	NO	MSL
MSL	LWK	0.3069	0.3106	3.58E-08	3.95E-05	YES	LWK
MSL	CEU	0.3069	0.2941	2.55E-66	2.81E-63	YES	MSL
MSL	GBR	0.3069	0.2937	1.18E-69	1.30E-66	YES	MSL
MSL	IBS	0.3069	0.2962	4.54E-48	5.01E-45	YES	MSL
MSL	TSI	0.3069	0.2958	4.47E-52	4.93E-49	YES	MSL
MSL	CLM	0.3069	0.3059	4.08E-01	1.00E+00	NO	MSL
MSL	PUR	0.3069	0.3109	1.25E-15	1.38E-12	YES	PUR
MSL	PEL	0.3069	0.2721	0.00E+00	0.00E+00	YES	MSL
MSL	MXL	0.3069	0.2963	8.84E-39	9.76E-36	YES	MSL
YRI	GWD	0.3076	0.3074	4.90E-01	1.00E+00	NO	YRI
YRI	ESN	0.3076	0.3067	5.85E-01	1.00E+00	NO	YRI
YRI	LWK	0.3076	0.3106	8.47E-08	9.35E-05	YES	LWK
YRI	CEU	0.3076	0.2941	4.82E-66	5.32E-63	YES	YRI
YRI	GBR	0.3076	0.2937	9.34E-69	1.03E-65	YES	YRI
YRI	IBS	0.3076	0.2962	9.92E-42	1.10E-38	YES	YRI
YRI	TSI	0.3076	0.2958	5.61E-46	6.19E-43	YES	YRI
YRI	CLM	0.3076	0.3059	2.13E-01	1.00E+00	NO	YRI
YRI	PUR	0.3076	0.3109	1.78E-16	1.97E-13	YES	PUR
YRI	PEL	0.3076	0.2721	0.00E+00	0.00E+00	YES	YRI
YRI	MXL	0.3076	0.2963	7.59E-36	8.38E-33	YES	YRI
GWD	ESN	0.3074	0.3067	5.50E-01	1.00E+00	NO	GWD
GWD	LWK	0.3074	0.3106	9.08E-08	1.00E-04	YES	LWK
GWD	CEU	0.3074	0.2941	2.09E-68	2.30E-65	YES	GWD
GWD	GBR	0.3074	0.2937	2.18E-68	2.41E-65	YES	GWD
GWD	IBS	0.3074	0.2962	6.39E-44	7.05E-41	YES	GWD
GWD	TSI	0.3074	0.2958	1.91E-48	2.11E-45	YES	GWD

GWD	CLM	0.3074	0.3059	1.05E-01	1.00E+00	NO	GWD
GWD	PUR	0.3074	0.3109	1.58E-18	1.75E-15	YES	PUR
GWD	PEL	0.3074	0.2721	0.00E+00	0.00E+00	YES	GWD
GWD	MXL	0.3074	0.2963	4.27E-34	4.71E-31	YES	GWD
ESN	LWK	0.3067	0.3106	3.76E-09	4.15E-06	YES	LWK
ESN	CEU	0.3067	0.2941	1.60E-62	1.77E-59	YES	ESN
ESN	GBR	0.3067	0.2937	1.86E-62	2.05E-59	YES	ESN
ESN	IBS	0.3067	0.2962	1.74E-43	1.92E-40	YES	ESN
ESN	TSI	0.3067	0.2958	3.54E-47	3.90E-44	YES	ESN
ESN	CLM	0.3067	0.3059	2.45E-02	1.00E+00	NO	ESN
ESN	PUR	0.3067	0.3109	2.61E-17	2.89E-14	YES	PUR
ESN	PEL	0.3067	0.2721	0.00E+00	0.00E+00	YES	ESN
ESN	MXL	0.3067	0.2963	1.34E-29	1.48E-26	YES	ESN
LWK	CEU	0.3106	0.2941	5.18E-111	5.72E-108	YES	LWK
LWK	GBR	0.3106	0.2937	2.18E-110	2.41E-107	YES	LWK
LWK	IBS	0.3106	0.2962	3.22E-86	3.56E-83	YES	LWK
LWK	TSI	0.3106	0.2958	1.40E-90	1.54E-87	YES	LWK
LWK	CLM	0.3106	0.3059	3.57E-04	3.94E-01	NO	LWK
LWK	PUR	0.3106	0.3109	8.85E-03	1.00E+00	NO	PUR
LWK	PEL	0.3106	0.2721	0.00E+00	0.00E+00	YES	LWK
LWK	MXL	0.3106	0.2963	5.09E-66	5.62E-63	YES	LWK
CEU	GBR	0.2941	0.2937	9.99E-01	1.00E+00	NO	CEU
CEU	IBS	0.2941	0.2962	6.44E-04	7.11E-01	NO	IBS
CEU	TSI	0.2941	0.2958	2.25E-02	1.00E+00	NO	TSI
CEU	CLM	0.2941	0.3059	4.66E-85	5.14E-82	YES	CLM
CEU	PUR	0.2941	0.3109	3.44E-140	3.79E-137	YES	PUR
CEU	PEL	0.2941	0.2721	2.24E-201	2.48E-198	YES	CEU
CEU	MXL	0.2941	0.2963	1.09E-11	1.20E-08	YES	MXL
GBR	IBS	0.2937	0.2962	2.66E-04	2.94E-01	NO	IBS
GBR	TSI	0.2937	0.2958	1.32E-02	1.00E+00	NO	TSI
GBR	CLM	0.2937	0.3059	3.22E-80	3.56E-77	YES	CLM
GBR	PUR	0.2937	0.3109	1.42E-141	1.57E-138	YES	PUR
GBR	PEL	0.2937	0.2721	2.67E-189	2.94E-186	YES	GBR
GBR	MXL	0.2937	0.2963	3.62E-12	3.99E-09	YES	MXL
IBS	TSI	0.2962	0.2958	2.29E-01	1.00E+00	NO	IBS
IBS	CLM	0.2962	0.3059	4.54E-59	5.01E-56	YES	CLM
IBS	PUR	0.2962	0.3109	3.54E-117	3.90E-114	YES	PUR
IBS	PEL	0.2962	0.2721	2.58E-249	2.85E-246	YES	IBS
IBS	MXL	0.2962	0.2963	9.44E-04	1.00E+00	NO	MXL
TSI	CLM	0.2958	0.3059	1.39E-64	1.54E-61	YES	CLM
TSI	PUR	0.2958	0.3109	4.01E-123	4.42E-120	YES	PUR
TSI	PEL	0.2958	0.2721	8.82E-231	9.74E-228	YES	TSI
TSI	MXL	0.2958	0.2963	1.72E-05	1.90E-02	NO	MXL
CLM	PUR	0.3059	0.3109	6.08E-11	6.71E-08	YES	PUR
CLM	PEL	0.3059	0.2721	0.00E+00	0.00E+00	YES	CLM
CLM	MXL	0.3059	0.2963	1.81E-38	1.99E-35	YES	CLM
PUR	PEL	0.3109	0.2721	0.00E+00	0.00E+00	YES	PUR
PUR	MXL	0.3109	0.2963	1.19E-86	1.32E-83	YES	PUR
PEL	MXL	0.2721	0.2963	2.14E-306	2.37E-303	YES	MXL

Table S12. **Genetic diversity.** Wilcoxon test comparing genetic diversity distributions showed in table S10 and multiple-test corrected by Bonferroni.

Population	First Contact	Date of the inflection point of the colonization process and main disruption event from their former life style
Guarani Kaiowá	16th century	17th century; Intensification of the attacks by bandeirantes against Guarani settlements and Guarani jesuitic reductions
Guarani Ñandevá	16th century	17th century; Intensification of the attacks by bandeirantes against Guarani settlements and Guarani jesuitic reductions
Urubu Kaapor	17th century	19th century; Migration from the lands between Tocantins and Xingu river to their actual location in Maranhão state after conflicts with the colonizers and other native peoples
Karitiana	18th century	19th century; Migration fleeding from colonizers from the area around Cuiabá, Mato Grosso, to the Northwest, in the border between Mato Grosso and Rondonia states, their actual location.
Surui	19th century	20th century; Arrival of rubber collectors at the beginning of the century and final settlement and colonization in the mid 20th century
Zoro	19th century	20th century; Permanent settlement of northwestern Mato Grosso state, construction of roads and telegraphic lines.
Apalai	17th century	20th century; Growth of chestnut and rubber commerce. Other ommercial relationships with colonizers before and after.
Arara	19th century	20th century; Arrival of rubber collectors and persecution
Xavante	17th century	18th century; Migration forced by the colonization pressure from eastern margin of Araguaia river to their actual location

Table S13. European contact and main events of the colonization process. Approximated dates of the first known European contact and dates of the main event that implied a point of inflection on the disruption of the former life style of the Native American populations, due to the of advance of the colonization process (Carneiro da Cunha 1998; Teixeira-Pinto 1998; Ferreira Thomaz de Almeida and Mura 2003; Kanindé Associação de Defesa Etnoambiental 2003; Storto 2005; Dal Poz 2009; Morgado 2013).

	Salvador Rec	Bambui Rec	Pelotas Rec	Salvador	Bambui	Pelotas
1st	0	0	0	0	0	0
rel. freq	0.00000	0.00000	0.00000	0.00000	0.00000	0.00000
2nd	0	0	0	5	63	43
rel. freq	0.00000	0.00000	0.00000	0.00001	0.00015	0.00001
3rd	7	340	62	157	889	871
rel. freq	0.00549	0.34343	0.00321	0.00020	0.00208	0.00013
Unrelated	1268	650	19244	775473	427323	6669464
rel. freq	0.99451	0.65657	0.99679	0.99979	0.99778	0.99986

Table S14. **KING** analysis of the degree of relatedness between individuals of the same population before and after the reconstruction. The numbers correspond to the amount of pairs of individuals in each degree of relatedness. KING(Manichaikul et al. 2010) has been used with default parameters to compute relatedness through IBD fragments for both admixed and Reconstructed populations from Bambui, Pelotas and Salvador.

References

Campbell L, Grondona V. 2012. The Indigenous Languages of South America. (Hock HH, editor.). Göttingen: Hubert & Co. GmbH & Co. KG

Carneiro da Cunha M. 1998. Historia dos indios no Brasil. São Paulo: Editora Schwarcz Ltda

Dal Poz J. 2009. Zoró. Inst. Socioambiental | Povos Indígenas no Bras.

Danecek P, Auton A, Abecasis G, Albers CA, Banks E, DePristo MA, Handsaker RE, Lunter G, Marth GT, Sherry ST, et al. 2011. The variant call format and VCFtools. Bioinformatics 27:2156–2158.

Ferreira Thomaz de Almeida R, Mura F. 2003. Guarani Kaiowá. Inst. Socioambiental | Povos Indígenas no Bras.

Kanindé Associação de Defesa Etnoambiental. 2003. Surui. Inst. Socioambiental | Povos Indígenas no Bras.

Manichaikul A, Mychaleckyj JC, Rich SS, Daly K, Sale M, Chen W-M. 2010. Robust relationship inference in genome-wide association studies. Bioinformatics 26:2867–2873.

Morgado P. 2013. Aparai. Inst. Socioambiental | Povos Indígenas no Bras.

Storto L. 2005. Karitiana. Inst. Socioambiental | Povos Indígenas no Bras.

Teixeira-Pinto M. 1998. Arara. Inst. Socioambiental | Povos Indígenas no Bras.
